# Intra-articular injection of bone marrow aspirate concentrate (mesenchymal stem cells) in KL grade III and IV knee osteoarthritis: 4 year results of 37 knees

**DOI:** 10.1038/s41598-024-51410-2

**Published:** 2024-02-01

**Authors:** Christof Pabinger, Harald Lothaller, Georg Stefan Kobinia

**Affiliations:** 1 IRM - Institute for Regenerative Medicine, Plüddemanngasse 45, 8010 Graz, Austria; 2 Medical University of Innsbruck, Christof Probst Platz 1, 6020 Innsbruck, Austria; 3 University of Music and Performing Arts, Leonhardstraße 15, 8010 Graz, Austria

**Keywords:** Osteoarthritis, Stem cells

## Abstract

Cell based therapies are increasingly used and results of bone marrow aspirate concentrate (BMAC) show encouraging short- to middle term results, superior to hyaluronic acid and platelet rich plasma (PRP). Most studies describe patients with mild to moderate arthritis and results of patients with KL III and IV osteoarthritis of the knee are limited to short term evaluations. Hence, the aim of this prospective study was to investigate the mid-term outcome of BMAC injections in patients with severe osteoarthritis of the knee. The BMAC was retrieved from the iliac crest as previously published with the “reorientation technique” from the iliac crest in supine position in analgosedation and injected into the patients’ osteoarthritic knees. Patients were followed-up for 4 years. WOMAC, IKDC, SF 36 and walking distance were measured in a total of 37 participants. There was an improvement of IKDC and WOMAC from the first year onwards and a significant improvement beginning from year 2 up to the mid-term follow-up: IKDC increased significantly from 56 ± 12 (range 34–81) to 73 ± 13 (range 45–100), *p* < 0.001. WOMAC decreased significantly from 40 ± 23 (range 6–96) to 18 ± 18 (range 0–67), *p* < 0.001. 35 of 37 knees improved regarding IKDC and WOMAC score from the first to the last follow-up. Not a single protheses had to be implanted. Elaborate statistical analysis was done to exclude covariates and confounders (age, time, BMI,…). In summary, this is the first study on BMAC injections into 37 osteoarthritic knees with a 4-year follow up showing significant improvements in IKDC and WOMAC scores, and with a 95% success rate and significant improvement in walking distance.

*Clinical relevance *Describes the 4-year outcome of BMAC injections for knees with severe osteoarthritis.

## Introduction

Osteoarthritis (OA) is the most prevalent chronic joint disease and a leading cause of disability and source of high societal cost in older adults^1^. Globally, prevalent cases of OA increased by 113%, from 248 million in 1990 to 528 million in 2019, and OA of the knee contributed the most to the overall burden^2^. Due to epidemiologic circumstances (age, obesity, sports activity, birth rates, gender,…), the number of knee arthroplasties will therefore increase exponentially worldwide, which will result in challenging health care expenditures and an unmet demand for orthopedic surgeons in most OECD countries in the future^3^. Furthermore a possible revision of a knee implant remains an additional burden^4–6^.

Therefore increasing use of biologic agents has been seen in the treatment of knee arthritis^7^. Patients with knee osteoarthritis who receive platelet-rich plasma (PRP) or bone-marrow aspirate concentrate (BMAC) injections (also described as “mesenchymal stem cell therapy”) have better outcomes than patients who receive hyaluronic acid^8–10^. However, the effect remains not fully understood in vivo, because PRP administration did not modulate significant changes of cytokine concentrations either in synovial fluid or plasma, whatever the time points analyzed, but was able to improve clinical outcome significantly^11,12^.

Recently the administration of so called “mesenchymal stem cells” has lead to a hype: A total of 22 (22%) of the 98 mesenchymal stem cell clinical trials currently registered on EudraCT involve skeletal applications (bone, tendon repair, osteoarthritis)^13^. However, in most studies, bone marrow aspirate (BMA) or bone marrow aspirate concentrate (BMAC) is used.

Feasibility and safety of injections of BMA or BMAC for knee arthritis were confirmed, indications of clinical efficacy were identified and significant improvement in function (in comparison to hyaluronic acid and/or PRP) was demonstrated in multiple randomized controlled trials since the year 2013^8,9,14–19^.

Initially it was assumed, that bone marrow derived cells would deteriorate with increasing age^20^, but recently a 70-fold greater study demonstrated, that the number of stem cells remains more or less unchanged in adult people^21^. However, there are differences regarding the number of cells between the different anatomic sites^20,22^: Iliac crest (anterior or posterior are equal) outperforms tibia: The different harvesting techniques have been described in detail by Olivier^23^, Hernigou^24^ and Pabinger^22^.

All cited studies above concern only patients with arthritis grade I-III according to Kellgren-Lawrence^25^. To our knowledge there is only one systematic review concerning grade IV arthritis of the knee joint and there was no evidence^26^.

The aim of the current study is, to prospectively evaluate the clinical outcome of patients with severe osteoarthritis of the knee (Kellgren Lawrence Grade III and IV) after injection of bone marrow aspirate concentrate (BMAC) intraarticulary. This is the first study with a follow up of 4 years, as compared to previous studies, referring only to a follow up of 6–12 or 24 month, respectively. Furthermore this is the the first study reporting long term results in patients with severe osteoarthritis undergoing BMAC injections.

## Material and methods

### Study design and patient selection

The current prospective multi-center cohort study was approved by the Ethics Committee of the Medical University of Graz (Ethikkkommission der Medizinische Universität Graz, Auenbruggerplatz 2, 8036 Graz, Austria /EU, 31-152 ex 18/19). All methods were performed in accordance with the relevant guidelines and regulations and informed consent was obtained from all participants and/or their legal guardians in accordance with the Declaration of Helsinki. The following centers have been involved in the clinical trial: Institute for regenerative Medicine (Graz, Asutria) and Private Clinic Leech (Graz, Austria). Patients were enrolled between 2019 and 2022. Treatment indications included symptomatic knee OA. Moreover, the following inclusion criteria were used:

- Patients aged between 40 and 90 years
- Radiographic severity grade III and IV according to the Kellgren-Lawrence classification
- Failure after at least 12 months of conservative treatment (patients not responsive to physiotherapy, NSAIDs, intraarticular injective treatment or scheduled for knee arthroplasty)
- Patients physically and mentally able to comply with the study requirements and with scheduled follow-up.

The following exclusion criteria were considered:

- Patients lacking understanding capacity
- Previous knee trauma within 24 months
- Intra-articular injection in the past 12 months
- Knee surgery in the last 24 months
- Untreated knee instability
- Malalignment of the leg-axis > 10°
- History of malignant neoplasia or rheumatic disease
- Presence of metabolic disorders including diabetes, thyroid metabolic diseases, history of alcohol or drug abuse; body mass index < 19 or > 30, Vitamin D deficiency of < 30 ng/ml
- Persistant history of bone marrow edema bigger than 2cm in diameter over 12 months.

Thirty-seven knees of 29 patients were prospectively and consecutively enrolled according to the inclusion/exclusion criteria. Among them 11 women and 18 men with a mean age of 58 ± 16 years. 13 knees in 10 patients had already undergone previous surgery in the index knee. Further demographics see Table 1.

**Table 1:** Patient demographics (37 knees).

Knees (male / female)	25/12
Patients (male / female)	18/11
Age, years (mean ± SD)	58 ± 18
BMI, kg/cm^2^ (mean ± SD)	25 (18–35)
Side (left / right)	21/16
Symptoms duration, months (range)	58 (24–96)
Kellgren Lawrence	Grade III: 15 (5*)
	Grade IV: 22 (8*)

*Previous surgery.

### Procedure

All patients were treated by a single surgeon with established experience in cartilage regenerative procedures. The procedure was performed with the patient in supine position under sedoanalgesia. The ipsilateral iliac crest and knee were scrubbed surgically and draped. In the ventral third of the crista iliaca a 2 mm stich incision was made with previously published reorientation technique^22^ and 6 × 10 ml of bone marrow aspirate (BMA) was retrieved with an Arthex Angel needle. The BMA was coated with citrate anticoagulant and then concentrated using the Arthex Angel centrifuge (https://www.arthrex.com/de/orthobiologie/arthrex-angel-system). We used a cell concentration factor of 7% for this study in all patients, as recommended by the manufacture and 15 min of centrifugation. We obtained 5ml bone marrow aspiration concentrate (BMAC) out of the 60ml BMA for the injection. The BMAC was then injected intra-articularly using a lateral suprapatellar approach into the respective knee. In patients with bilateral OA the procedure was repeated on the other side in the same manner.

Postoperatively, patients were discharged on the same day. Pain control was prescribed as needed with a NSAID on the same day. Cryotherapy was prescribed for 3 days. Full weight bearing was allowed immediately. Cessation of sports was recommended for 1–2 weeks. No thromboembolic prophylaxis was given. No specific physiotherapy was prescribed.

### Patients’ evaluation

All patients were clinically evaluated on the postoperative day and in week six, thereafter in year 1, 2, 3, 4 and 5. To evaluate treatment safety, all complications and adverse effects were assessed by the physician at the follow-ups or by the patient between the follow-ups. Serious events were defined as any event that resulted in death or life-threatening, required hospitalization or intervention or lead to permanent impairment or damage. Minor adverse events were defined as significant pain, swelling, hematoma or symptoms limiting daily life activities.

*Primary outcome parameters* were the International Knee Documentation Committee score (IKDC) and the Western Ontario and McMaster Universities Osteoarthritis Index (WOMAC).

*Secondary parameters *were two items from the SF-36 (SF 36 Q1: “how would you rate your current state of health?” and SF 36 Q2: “How would you rate your current state of health as compared to last year?”), walking distance, age, sex, weight, height, BMI, days postoperatively and the question “would you undergo this surgery again?”.

All parameters were evaluated preoperatively and postoperatively at any point of time.

The treatment was defined to have failed if the patient needed a new surgical procedure regarding the respective knee (e.g. total knee replacement) or an injection procedure (steroid, hyaluronic acid, PRP, …) due to persisting or worsening of symptoms.

### Statistical analysis

All continuous data were expressed in terms of the mean and the standard deviation of the mean, the categorical data were expressed as frequencies and percentages.

As main analyses, paired t-tests were performed to compare the preoperative against the last postoperative follow-up measure of IKDC, WOMAC, SF36 and walking distance over time.

Additional analyses of variance and Person`s or Spearman correlations as well as Mann–Whitney-U-tests were performed using primary and secondary outcomes (IKDS, WOMAC, SF 36, walking distance, age, sex, height, weight, BMI). In particular, ANOVAs with repeated measured, considering sex as independent variable, were done to control for group differences and interactions. Other demographic parameters did not correlate substantially with the primary outcomes to be considered as independent variables or covariates consequently.

Analyses were done using IBM SPSS Statistics 28. The significance level was set at five percent.

It shall be noted that preconditions for the parametric analyses of differences of the means were performed when needed. The Kolmogorov–Smirnov-test showed a normal distribution of data at any point of time (preoperative and all postoperative follow-ups) and any outcome. The Levene’s test showed homogeneous variances when comparing unpaired groups.

The tests for the primary outcomes were repeated unpaired because of additional data for more than one postoperative point of time for some patients. Results were similar to the paired analyses. Furthermore, analyses comparing the years (1–4 years after) could be done with this approach.

Patients with knee osteoarthritis grade III and IV were independently assessed in detail: For paired comparisons between pre- and post-values, an ANOVA with repeated measures with grade as additional factor was calculated.

## Results

### Primary outcome parameters

**Figure 1:**
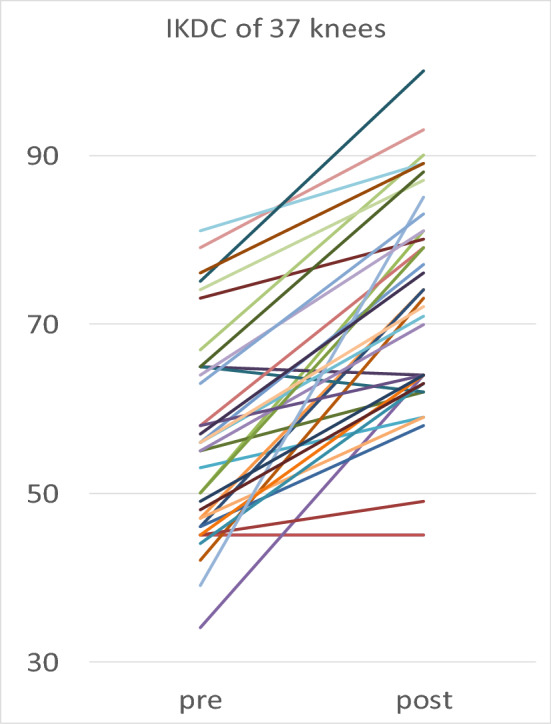
Pre- and last postoperative results show improvement in 35 of 37 knees (95% of all cases) in both scores.

**Figure 2:**
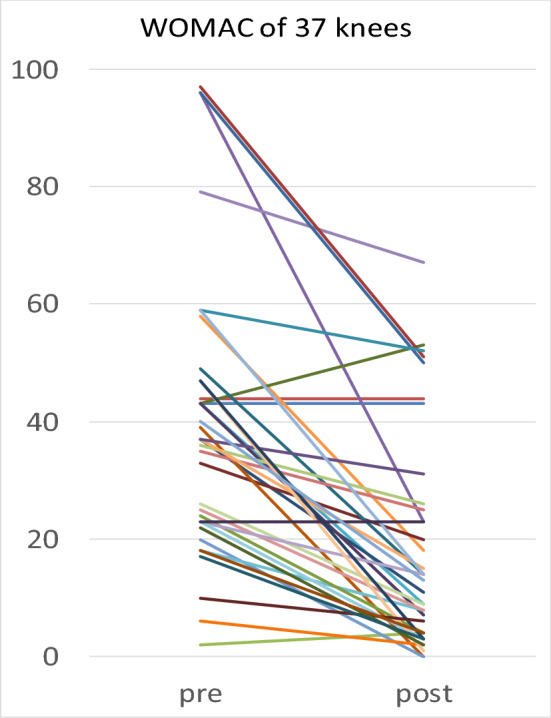
Pre- and last postoperative results show improvement in 35 of 37 knees (95% of all cases) in both scores.

All knees except one patient (2 knees) were found to have an increase in both primary parameters, which can be reflected as a 95% rate of measurable success. One patient (2 knees) worsened slightly, but he was still able to walk 3 h (15 km) postoperatively, see Figs. 1 and 2 and Table 2.

**Table 2:** Paired analyses for 37 KL III and IV OA knees.

	n	pre	post	*p*
IKDC				
Grade III*	15	61 ± 14	78 ± 12	< 0.001
Grade IV*	22	53 ± 9	68 ± 13	< 0.001
All	37	56 ± 12	72 ± 13	< 0.001
WOMAC				
Grade III*	15	41 ± 30	13 ± 16	< 0.001
Grade IV*	22	38 ± 15	22 ± 19	< 0.001
All	37	40 ± 23	19 ± 18	< 0.001

*Kellgren Lawrence arthritis.

**Figure 3:**
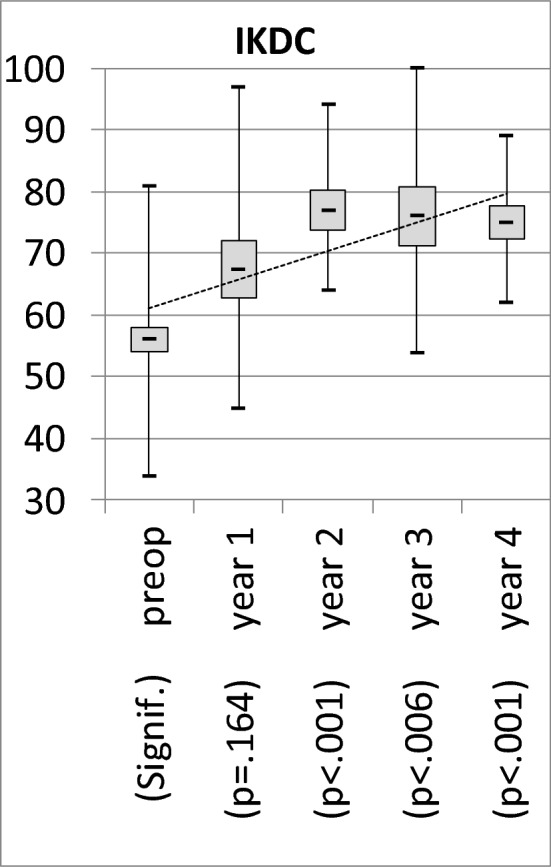
Mean, Min, Max and 95%-CI Intervals of the primary outcome parameters showed significant and relevant improvement of injection of bone marrow aspirate concentrate into the respective knee from year 2 on. Pre- and postoperative results show significant improvement up to year 4 in both scores.

**Figure 4:**
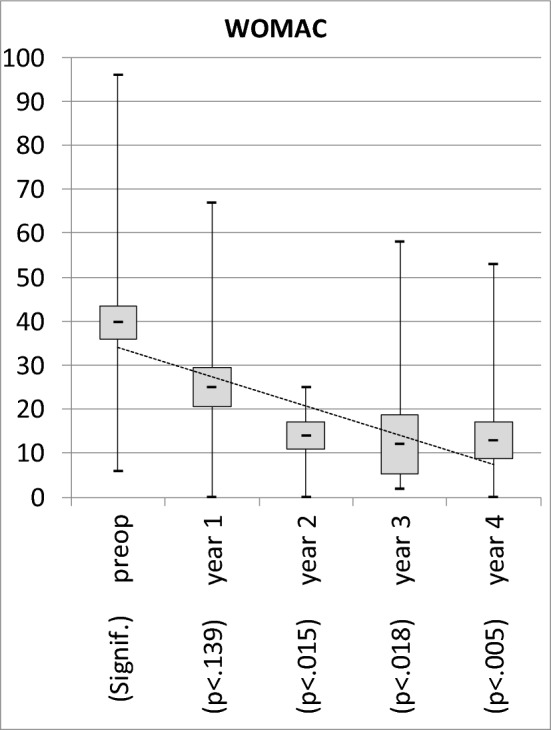
Mean, Min, Max and 95%-CI Intervals of the primary outcome parameters showed significant and relevant improvement of injection of bone marrow aspirate concentrate into the respective knee from year 2 on. Pre- and postoperative results show significant improvement up to year 4 in both scores.

There was a significant and relevant improvement of IKDC and WOMAC over time beginning from year 2 on: IKDC increased significantly from 56 ± 12 (range 34–81) to 73 ± 13 (range 45–100), *p* < 0.001. WOMAC decreased significantly from 40 ± 23 (range 6–96) to 18 ± 18 (range 0–67), *p* < 0.001. 35 of 37 knees improved regarding IKDC and WOMAC score from the first to the last follow-up. Detailed results see Figs. 3 and 4.

Days postoperatively was analyzed as covariate analyses and did not alter results. There was a positive correlation between preoperative and postoperative primary parameters: Patients with previously higher IKDC showed a higher IKDC postoperatively as well (r = 0.641, *p* < 0.001) and patients with previously higher WOMAC showed a higher WOMAC postoperatively as well (r = 652, *p* < 0.001). Covariate analysis confirmed, that the improvement from preoperatively to postoperatively in IKDC and WOMAC was statistically significant (*p* < 0.001) in both genders (See Figs. 1 and 2).

Preoperatively women had a significantly lower IKDC as compared to men (51 ± 12 versus 59 ± 11, *p* < 0.029), whereas postoperatively there was no gender-difference (72 ± 11 versus 72 ± 14, *p* = 0.97). Regarding WOMAC women did not show a significantly lower score as compared to man, neither preoperatively (45 ± 30 versus 36 ± 16, *p* < 0.072) nor postoperatively (17 ± 16 versus 19 ± 19, *p* = 0.074).

Based on the Questions from the WOMAC and the IKDC, the selected items changed from pre- to postoperatively as follows, see Table 3:

**Table 3:** Specific outcome parameters of 37 KL III and IV OA knees after BMAC injection (“mesenchymal stem cell therapy”).

Pain: (0 = no pain, 4 = extreme pain)	pre-	Postoperatively
Pain while walking:	2.0	0.9
Pain during the night:	1.4	0.5
Pain during sitting:	1.2	0.4
Stiffness: (0 = no stiffness, 4 = extreme stiffness)		
Morning stiffness:	2.2	1.3
After sitting:	1.9	1.0
Physical activity (0 = no limitation, 4 = extreme limitation)		
Walking down stairs:	2.6	1.3
Walking up stairs:	1.9	0.8
Get in/out of the car:	1.7	0.9
Severy household work:	2.6	1.1
Light household work:	1.6	0.6
Activity:	2.2	2.8
2: Walking, light household acticities		
3: moderate activities like Running, jogging, moderate household activities		
Pain: (0 = always, 10 = never)	3.4	6.4
Functionality:	5.3	7.5
0 = unable to perform daily activities, 10 = no limitations		

The patients with knee OA grade III and IV were assessed separately as follows in Table 2, no interaction was found:

### Secondary outcome parameters

All patients except one (2 knees) would undergo the surgery again, which can be reflected as a 95% rate of subjective success.

Walking distance improved significantly from pre- to postoperatively (*p* < 0.001; from 3.4 (± 1.2) to 4.1 (± 1), *p* = 0.04; ordinal data: “1: home, 2: < 15min, 3: < 30min, 4: > 30min, 5: indefinite”). Walking distance correlated with SF 36 question 2 (r = 0.380, *p* = 0.020), BMI (r = − 0.373, *p* = 0.023) and IKDC (r = 0.679, *p* < 0.001) and time (r = 0.404, *p* < 0.001) preoperatively and with time (i.e. years after; r = 0.354, *p* = 0.012) and IKDC (r = 0.571, *p* < 0.001) postoperatively, with only a tendency remaining for BMI (r = − 0.265, *p* = 0.066).

SF 36 questions 1 correlated significantly and strong (r = 0.632, *p* < 0.001) preoperatively and postoperatively, but question 2 did not (r = 0.272, *p* = 0.103). For both SF 36 questions, there were no differences over time (*p* = 0.564 and *p* = 0.664). Also no differences between sexes and not interactions were found in additional analyses.

SF 36 question 1 was not correlated to any other parameter neither pre- nor postoperatively. Preoperatively, SF 36 Q2 was correlated with IKDC (r = 0.541, *p* < 0.001) and walking ability (r = 0.380, *p* = 0.020). Postoperatively, no significant correlations with any other parameter were found but only a tendency with IKDC remains (r = 0.277, *p* = 0.054).

The results of the eight patients with bilateral injections did not differ with respect to the 21 patients with monolateral knee OA and injection.

All other secondary parameters (age, sex, weight, height, days postoperatively) did not correlate significantly with each other or with the primary or secondary parameters and no relevant covariates were found.

### Failures, complications and adverse effects

On the first postoperative day all patients were free of pain regarding the iliac crest. A third of all patients reported mild soreness in the respective knee for 1 day to 1 week, but all were able to work the next day and to travel home on the same day.

In one patient the bone marrow aspirate clotted during the centrifugation and could not be injected and the procedure (BMA-aspiration and BMAC injection) was repeated in the following month. In a second patient rheumatoid arthritis was diagnosed in the fourth postoperative year. In a third patient the nervus cutaneous femoris lateralis was numb for 8 h postoperatively due to the long-lasting effect of the local anaesthesia (ropivacaine) from the BMA puncture on the crista iliaca, but no muscular deficit or alteration in range of motion was noted. In 5% of all knees (2 knees, one patient) there was no positive effect.

## Discussion

To our knowledge this is the first study worldwide with a long term follow (> 4 years) of patients who suffer from severe degenerative osteoarthritis of the knee (grade III and grade IV) and underwent an injection of bone marrow aspirate concentrate (BMAC, also described as “MSCs, mesenchymal stem cells” or “stem cell therapy”^13,27^).

Up to now only articles on BMAC injections were published with a short term follow up (6–12-24 months), regardless of the respective joint:^8,9,15–17,19,28–32^.

In 2013 one of the first studies worldwide on the application of autologous pluripotent (stem) cells described the injection of autologous expanded bone marrow MSCs in 12 patients with knee arthritis, followed for a year^17^. However, as a matter of fact, a major limitation in the whole present literature is its paucity of detailed information, what the authors actually did in the respective studies: Of the 1580 studies identified, considerable deficiencies in the reporting of key variables, including the details of stem cell processing, possible culture conditions, and the characteristics of cell populations delivered, were noted. Overall, studies reported only 52% of variables that may critically influence outcome^33,34^.

In 2018 a review article of 1832 papers dealing with [“bone marrow aspirate” and “cartilage”] or [“mesenchymal stem cells” and “cartilage”] demonstrated promising results in the clinical application for repair of chondral defects and early clinical data suggests BMAC may help stimulate a more robust hyaline cartilage repair tissue response. Therefore, the current study was started^18^.

In 2021 a review on BMA or BMAC in osteoarthritis confirmed an remarkably increased publication trend over time. Safety was again confirmed by all studies, with a low number of adverse events, and an overall improvement in pain and function was documented in most of the studies, however, no long term results available yet^35^. This is well in line with our findings, where no mild or severe adverse effects were noted.

The outcome of BMAC in knee OA was already compared to the current gold standard therapies in the last years: Undoubted PRP and BMAC are superior to hylauronic acid (HA), as confirmed in a large review in the year 2023^8,36^. Regarding BMAC alone, a significant improvement in WOMAC after BMAC injection plus a reduction of VAS values as compared to PRP was found at six months^15^. In another study, the BMAC group significantly improved in VAS, KOOS, and WOMAC scores between baseline and 12 months; in contrast, the PRP group (n = 13 knees) witnessed nonsignificant improvement in all scores^37^. Dulic showed, that OA patients treated with BMAC alone showed a significantly better VAS postoperatively, as compared to PRP or HA alone^9^.

However, these positive publications are in contrast to a review of 8 studies with a mean follow-up of 13 months, reporting the outcome of BMAC injections in OA knees: BMAC has not demonstrated clinical superiority in this paper in relation to other biologic therapies commonly used in the treatment of OA, including platelet-rich plasma and microfragmented adipose tissue, or in relation to placebo^38^. Fortunately in 34 of 36 (94.4%) patient-reported outcomes assessed across these 8 studies in the respective paper, BMAC demonstrated a significant improvement, and BMAC injection is effective in improving pain and patient-reported outcomes in patients with knee OA at short- to midterm follow-up. On a closer look, the follow-up of 13months in the respective studies was probably too short to notice a superior effect, which was demonstrated in the patients of the current study, where improvement was significant from the year 2 on and later, but not significant in the first year.

We found no effect of BMI and patient’s age on outcome in our data of 37 knees. This was confirmed in a study of 111 participants by Rasovic, who reported, that participants' age and BMI did not influence the clinical outcome, but there was an influence of OA severity, especially among older patients. His study shows that BMAC therapy is effective and he states, that younger patients with milder OA changes could be better candidates for long-lasting and more efficient BMAC therapy^39^. Kim assessed the outcome of BMAC injection in patients with grade I to grade IV degenerative arthritis of the knee and confirmed this findings, that a BMAC injection significantly improved both knee pain and functions in the patients with degenerative arthritis of knee. Also, the injection would be more effective in early to moderate phases^40^.

It can be assumed, that the possibly inferior effect in elderly patients with severe arthritis as reported by the authors above is rather a result of a poor donor site (osteoarthritis) than age alone, since we published earlier, that the number of pluripotent cells in the bone marrow remains constant in adults, up to eighty years^21^. From former studies we already assessed, that we injected 140 ± 98 CD34 cells /µl with a viability of 85 ± 8% and 12 ± 4 Leucocytes / nl with our methodology in this study^22^. However, the accurate number of mesenchymal stem cells cannot be calculated, since it can only be measured indirectly: In this context it should be emphasized, that the use of the term ‘pure MSCs’ should be avoided since mesenchymal cells are not separated from the other cells of the graft’s microenvironment, and consequently the procedures performed are straightforwardly addressed at concentrating tissues with minimal manipulation, originating bone marrow aspirate concentrates (BMAC)^26^.

In order to reduce the risk of manipulation and infection, to simplify the technique and to avoid the costly and time-consuming procedure of centrifugation in order to generate BMAC from BMA, multiple attempts have been made, to use BMA alone, which was then harvested with an improved technique: The use of a special reorientation technique and the use of a needle with multiple lateral holes (Magellan® or MarrowCellution®) seem to be two different options to improve the cell yield, as described^22^. This new technique can lead to a 50% reduction in the visual analog scale score for pain at 64 ± 26 weeks post-procedure, even in patients with severe arthritis of the knee, using this pure bone marrow aspiration technique^41^. Varady used BMA harvested with a specific cannula instead of BMAC and found significant improvements in early pain and function scores for knee OA^42^. Knee OA patients treated with this technique exhibited significant reductions in VAS pain scores and significant improvements in WOMAC scores that exceeded the minimal clinically important difference thresholds^43^. Vigano published a single-step technique with BMA obtained with a centrifuge-free process, employing a dedicated aspiration device and showed the effectiveness of the study device to harvest pure bone marrow with minimal peripheral blood contamination. The relevant content of MSCs resulted in the ability to counteract the catabolic cascade through a paracrine action. The clinical outcomes in patients affected by unicompartmental knee OA were encouraging in terms of pain reduction and functional improvement up to mid-term evaluation^44^.

Another attempt, to improve clinical results is, to inject the BMAC at the subchondral site instead of intraarticularily. Hernigou was the first, to publish impressive 12 year results in patients with corticoid induced Grad IV arthritis: During the same anesthesia, one knee received TKA; for the other knee, a bone marrow graft was delivered to the subchondral bone of the femur and tibia^45^. At the most recent follow-up (average of 12 years), six (out of 30) TKA knees needed subsequent surgery versus only one with cell therapy in patients and patients liked the BMAC knee more. A similar improvement of a subchondral injection of BMAC was described by Vad, who published 14 month results with an improvement of the mean WOMAC score from 58.2 points pre-procedure to 35.3 points post-procedure (*p* < 0.01) and a mean NRS-Pain score drop from 8.6 points to 2.8 (*p* < 0.01), respectively^46^.

Kon combined the intraarticular and the subchondral injection of BMAC and showed safety and positive clinical and radiological outcome up to 24 months in the treatment of symptomatic knee OA, with durable clinical results, a low failure rate, and a significant reduction of bone marrow edema^32^. The role of a bone marrow edema in arthritis is not yet fully understood and it might be a negative predictor of outcome^31^. We can confirm this hypothesis, since we had unfavourable results leading to total knee arthroplasty in two patients outside of this study, where we injected BMA in severe osteoarthritis knees with a long lasting Vitamin D deficiency or a history of persistent bone marrow edema in the respective knee one year prior to the operation. It is worth to conduct further studies with MR imaging preoperatively to identify the influence of bone marrow edema. Lychagin is currently working on this, he published already a significant reduction of pain based on the VAS score and a significant improvement in the patients' WOMAC score and in the overall KOOS score of patients who underwent a subchondral (= intraosseous) injection of PRP and suffered from knee arthritis and  bone marrow edema^31^. However, injection into the edema may improve the outcome.

But BMAC can also be compared to other cell-based therapies: Pintore compared BMAC and ADSC (adipose-derived stromal cells) in patients with knee OA: Both treatment groups demonstrated significant improvement pre-procedure to post-procedure in knee KOOS scores, knee OKS scores, and VAS pain scores. Patients with K-L grade II showed better functional and clinical outcomes than patients with K-L grades III and IV^47^. Mautner compared BMAC and ADSC and his data demonstrate significant improvement in pain and function with both injections in patients with symptomatic knee OA without a significant difference in improvement when comparing the two autologous tissue sources^48^. A meta-analysis was already performed and the ADSC injection had a significantly greater effect on pain reduction than did the BMAC injection, but the clinical effect of BMAC versus ADSC knee injection in patients with knee OA regarding WOMAC was equivalent^49^.

Park compared BMAC and Human Umbilical Cord Blood-Derived Mesenchymal Stem Cells (hUCB-MSC): Improved clinical outcomes were found in both BMAC and hUCB-MSC groups; however, no significant difference was observed. On second-look arthroscopy, the hUCB-MSC group showed better International Cartilage Repair Society Cartilage Repair Assessment grade compared with the BMAC group in varus knee OA patients^50^.

Reading about all these different techniques, it becomes increasingly complicated to pick the right patient for the right therapy. Therefore recently Smith developed a CDR (clinical decision rule (CDR) ) to identify people with knee osteoarthritis who are likely or unlikely to benefit from bone marrow aspirate concentrate (BMAC) injection: Multiple logistic regression analysis was used to determine which combination of risk factors predicted BMAC responsiveness. A simple CDR containing three variables predicted responsiveness to a single intraarticular knee BMAC injection with high accuracy. Those with lower initial pain levels (< 7/10), or high pain levels with previous surgery, could be predicted to benefit from a single BMAC injection^51^.

The downside of all these cellular therapies is, that they fall under restrictive legislative constraints, making it almost impossible to offer these therapies to patients in Germany, Austria or Switzerland, except you have a respective license as a hospital, as we do. Some of the cited applications can be viewed as Advanced Therapy Medicinal Products (ATMPs): The use of those requires the knowledge of diverse regulations and an extensive (year long) communication with the national/international authorities, up to the hospital exemption^52^.

We do not see total knee arthroplasty (TKA) as an alternative to BMAC injection, since the patients differ, but the results have to be compared. Therefore we offer BMAC injections to the following three groups of patients:

- To old to undergo TKA (e.g. due to cardic, pulmonary issues)
- Too young to undergo TKA (biological age versus demographic age)
- Ideal age for TKA, but does not like to have a big operation right now due to personal circumstances (takes care of ill family member, long holiday journey planned,..) TKA will be planned some years later.

Since we do not know, how long the positive effects of BMAC will last, we always have to bear in mind, that TKA is a well proven long lasting option with a low rate of failure: The pooled revision rate per 100 observed component years for TKA of all registry based studies worldwide was 0.6 and can be compared to 1.7 regarding unicondylar knee arthroplasty (UKA). This equals a 6% revision rate for TKA and a 17% revisions rate for UKA at 10 year follow up^3,5,6,53,54^. Since these data include all manufacturers, it can be seen in the Australian registries, that specific modern prostheses (my knee^®^ by MEDACTA or ATTUNE^®^ bei DePuy Synthes,…) perform slightly better. These data of knee replacement surgery have to be compared to a failure rate of 5% in the patients of the current study, which equals a failure rate of 1.35  per 100 observed patient years, which is therefore between TKA and UKA (2 failed / (4 years x 37 patients) x 100).

Another even lesss invasive therapy is Cognitive behaviour therapy (CBT): It showed sustainable effects through health-led cognitive behavioral-based group therapy and has a positive effect on pain, functional disability and psychological outcomes for knee osteoarthritis patients^55^. A recent meta-analysis suggests the durability of CBT-associated treatment benefits, supporting its role as a potential promising alternative or complementary intervention for patients with knee/hip osteoarthritis, especially against pain and insomnia^56^. The effect of CBT is also influenced by age, education and sociocultural environment: Patients with high mean pain catastrophizing scores may particularly benefit from CBT^57^.

A limitation of the current study is, that the CDR^51^. was not available when we started the study. We furthermore made no extensive pre- and postoperative MRI evaluation, since approximately half of the patients were indicated on plain radiographs and medical history only—according to our inclusion and exclusion criteria. We also made no double-blind assessment against placebo, since this would not have passed the ethics commission and we also did not use a control group (PRP or ADSC or HA), since most patients had already undergone therapy with PRP or HA. We furthermore did not assess all SF 36 questions and made no extensive psycho-social anamnesis (to exclude bias and confounders), but the unchanged SF36 values pre- and postoperatively indicate a stable social environment of our patients.

A strength of the current study, evaluating the positive 4-year effects of BMAC injections in OA- knees, is the long follow up and the sound and solid statistics (see supplementary files). The late onset (year 2–4) of the beneficial effects might be explained rather by the role of the cells (cytokines, exosomes and cartilage generation) than by a placebo effect (Supplementary Information 1), which was reported only in short term studies, see above.

In summary, this is the first study on BMAC injections (“mesenchymal stem cell therapy”) into 37 KL III and IV OA knees with a long term (four year) follow up and a relevant and significant improvement in terms of IKDC (from 56 ± 12 to 73 ± 13, *p* < 0.001) and WOMAC (40 ± 23 to 18 ± 18, *p* < 0.001), with a 95% success rate and significant improvement in walking distance.

### Supplementary Information

Supplementary Information 1.

Supplementary Information 2.

Supplementary Information 3.

Supplementary Information 4.

Supplementary Information 5.

Supplementary Information 6.

Supplementary Information 7.

Supplementary Information 8.

Supplementary Information 9.

## Data Availability

The datasets used and/or analysed during the current study available from the corresponding author on reasonable request.
