# Supplementary material for: Intra-articular injection of bone marrow aspirate concentrate (mesenchymal stem cells) in KL grade III and IV knee osteoarthritis: 4 year results of 37 knees
Source: Sci Rep. 2024 Feb 1;14:2665. doi: 10.1038/s41598-024-51410-2 (PMC10834500; doi:10.1038/s41598-024-51410-2)
Supplement: Supplementary file 1 — Supplementary Information 1. [file 41598_2024_51410_MOESM1_ESM.docx]

**Deskriptive Statistik**

| **Deskriptive Statistik** | | | | | | |
| --- | --- | --- | --- | --- | --- | --- |
|  | N | Minimum | Maximum | Mittelwert | | Standardabweichung |
|  | Statistik | Statistik | Statistik | Statistik | Standardfehler | Statistik |
| t0_BMI | 37 | 18,3655 | 34,7209 | 24,725069 | ,7125741 | 4,3344189 |
| t1_BMI | 37 | 18,3655 | 34,7209 | 24,566901 | ,7149121 | 4,3486404 |
| Gültige Werte (Listenweise) | 37 |  |  |  |  |  |

**Deskriptive Statistik**

**t0_Geschlecht = 0**

| **Deskriptive Statistik** | | | | | | |
| --- | --- | --- | --- | --- | --- | --- |
|  | N | Minimum | Maximum | Mittelwert | | Standardabweichung |
|  | Statistik | Statistik | Statistik | Statistik | Standardfehler | Statistik |
| t0_BMI | 14 | 18,3655 | 34,7209 | 21,783820 | 1,2382373 | 4,6330598 |
| t1_BMI | 14 | 18,3655 | 34,7209 | 21,633157 | 1,2494026 | 4,6748365 |
| Gültige Werte (Listenweise) | 14 |  |  |  |  |  |

**t0_Geschlecht = 1**

| **Deskriptive Statistik** | | | | | | |
| --- | --- | --- | --- | --- | --- | --- |
|  | N | Minimum | Maximum | Mittelwert | | Standardabweichung |
|  | Statistik | Statistik | Statistik | Statistik | Standardfehler | Statistik |
| t0_BMI | 23 | 21,7993 | 34,3490 | 26,515395 | ,6325433 | 3,0335709 |
| t1_BMI | 23 | 21,4619 | 34,3490 | 26,352658 | ,6331422 | 3,0364431 |
| Gültige Werte (Listenweise) | 23 |  |  |  |  |  |

**Deskriptive Statistik**

| **Deskriptive Statistik** | | | | | | |
| --- | --- | --- | --- | --- | --- | --- |
|  | N | Minimum | Maximum | Mittelwert | | Standardabweichung |
|  | Statistik | Statistik | Statistik | Statistik | Standardfehler | Statistik |
| BMI | 86 | 18,3655 | 34,7209 | 24,682172 | ,4499972 | 4,1731025 |
| Gültige Werte (Listenweise) | 86 |  |  |  |  |  |

**Deskriptive Statistik**

**Geschlecht = 0**

| **Deskriptive Statistik** | | | | | | |
| --- | --- | --- | --- | --- | --- | --- |
|  | N | Minimum | Maximum | Mittelwert | | Standardabweichung |
|  | Statistik | Statistik | Statistik | Statistik | Standardfehler | Statistik |
| BMI | 32 | 18,3655 | 34,7209 | 21,754841 | ,7648776 | 4,3268013 |
| Gültige Werte (Listenweise) | 32 |  |  |  |  |  |

**Geschlecht = 1**

| **Deskriptive Statistik** | | | | | | |
| --- | --- | --- | --- | --- | --- | --- |
|  | N | Minimum | Maximum | Mittelwert | | Standardabweichung |
|  | Statistik | Statistik | Statistik | Statistik | Standardfehler | Statistik |
| BMI | 54 | 21,4619 | 34,3490 | 26,416887 | ,4022925 | 2,9562341 |
| Gültige Werte (Listenweise) | 54 |  |  |  |  |  |

**Deskriptive Statistik**

**Vorher_Nachher = 0**

| **Deskriptive Statistik** | | | | | | |
| --- | --- | --- | --- | --- | --- | --- |
|  | N | Minimum | Maximum | Mittelwert | | Standardabweichung |
|  | Statistik | Statistik | Statistik | Statistik | Standardfehler | Statistik |
| BMI | 37 | 18,3655 | 34,7209 | 24,725069 | ,7125741 | 4,3344189 |
| Gültige Werte (Listenweise) | 37 |  |  |  |  |  |

**Vorher_Nachher = 1**

| **Deskriptive Statistik** | | | | | | |
| --- | --- | --- | --- | --- | --- | --- |
|  | N | Minimum | Maximum | Mittelwert | | Standardabweichung |
|  | Statistik | Statistik | Statistik | Statistik | Standardfehler | Statistik |
| BMI | 49 | 18,3655 | 34,7209 | 24,649781 | ,5845948 | 4,0921635 |
| Gültige Werte (Listenweise) | 49 |  |  |  |  |  |

**Deskriptive Statistik**

**ZEITPUNKT = 0**

| **Deskriptive Statistik** | | | | | | |
| --- | --- | --- | --- | --- | --- | --- |
|  | N | Minimum | Maximum | Mittelwert | | Standardabweichung |
|  | Statistik | Statistik | Statistik | Statistik | Standardfehler | Statistik |
| BMI | 37 | 18,3655 | 34,7209 | 24,725069 | ,7125741 | 4,3344189 |
| Gültige Werte (Listenweise) | 37 |  |  |  |  |  |

**ZEITPUNKT = 1**

| **Deskriptive Statistik** | | | | | | |
| --- | --- | --- | --- | --- | --- | --- |
|  | N | Minimum | Maximum | Mittelwert | | Standardabweichung |
|  | Statistik | Statistik | Statistik | Statistik | Standardfehler | Statistik |
| BMI | 13 | 18,3655 | 34,3490 | 24,096959 | 1,2255616 | 4,4188251 |
| Gültige Werte (Listenweise) | 13 |  |  |  |  |  |

**ZEITPUNKT = 2**

| **Deskriptive Statistik** | | | | | | |
| --- | --- | --- | --- | --- | --- | --- |
|  | N | Minimum | Maximum | Mittelwert | | Standardabweichung |
|  | Statistik | Statistik | Statistik | Statistik | Standardfehler | Statistik |
| BMI | 13 | 19,0311 | 34,7209 | 25,164585 | 1,1971029 | 4,3162159 |
| Gültige Werte (Listenweise) | 13 |  |  |  |  |  |

**ZEITPUNKT = 3**

| **Deskriptive Statistik** | | | | | | |
| --- | --- | --- | --- | --- | --- | --- |
|  | N | Minimum | Maximum | Mittelwert | | Standardabweichung |
|  | Statistik | Statistik | Statistik | Statistik | Standardfehler | Statistik |
| BMI | 10 | 18,7783 | 32,2831 | 26,076036 | 1,2338776 | 3,9018635 |
| Gültige Werte (Listenweise) | 10 |  |  |  |  |  |

**ZEITPUNKT = 4**

| **Deskriptive Statistik** | | | | | | |
| --- | --- | --- | --- | --- | --- | --- |
|  | N | Minimum | Maximum | Mittelwert | | Standardabweichung |
|  | Statistik | Statistik | Statistik | Statistik | Standardfehler | Statistik |
| BMI | 13 | 18,4240 | 29,5858 | 23,590677 | 1,0333119 | 3,7256589 |
| Gültige Werte (Listenweise) | 13 |  |  |  |  |  |

**Deskriptive Statistik**

**Geschlecht = 0, Vorher_Nachher = 0**

| **Deskriptive Statistik** | | | | | | |
| --- | --- | --- | --- | --- | --- | --- |
|  | N | Minimum | Maximum | Mittelwert | | Standardabweichung |
|  | Statistik | Statistik | Statistik | Statistik | Standardfehler | Statistik |
| BMI | 14 | 18,3655 | 34,7209 | 21,783820 | 1,2382373 | 4,6330598 |
| Gültige Werte (Listenweise) | 14 |  |  |  |  |  |

**Geschlecht = 0, Vorher_Nachher = 1**

| **Deskriptive Statistik** | | | | | | |
| --- | --- | --- | --- | --- | --- | --- |
|  | N | Minimum | Maximum | Mittelwert | | Standardabweichung |
|  | Statistik | Statistik | Statistik | Statistik | Standardfehler | Statistik |
| BMI | 18 | 18,3655 | 34,7209 | 21,732302 | ,9922715 | 4,2098514 |
| Gültige Werte (Listenweise) | 18 |  |  |  |  |  |

**Geschlecht = 1, Vorher_Nachher = 0**

| **Deskriptive Statistik** | | | | | | |
| --- | --- | --- | --- | --- | --- | --- |
|  | N | Minimum | Maximum | Mittelwert | | Standardabweichung |
|  | Statistik | Statistik | Statistik | Statistik | Standardfehler | Statistik |
| BMI | 23 | 21,7993 | 34,3490 | 26,515395 | ,6325433 | 3,0335709 |
| Gültige Werte (Listenweise) | 23 |  |  |  |  |  |

**Geschlecht = 1, Vorher_Nachher = 1**

| **Deskriptive Statistik** | | | | | | |
| --- | --- | --- | --- | --- | --- | --- |
|  | N | Minimum | Maximum | Mittelwert | | Standardabweichung |
|  | Statistik | Statistik | Statistik | Statistik | Standardfehler | Statistik |
| BMI | 31 | 21,4619 | 34,3490 | 26,343800 | ,5290883 | 2,9458391 |
| Gültige Werte (Listenweise) | 31 |  |  |  |  |  |

**Deskriptive Statistik**

**Geschlecht = 0, ZEITPUNKT = 0**

| **Deskriptive Statistik** | | | | | | |
| --- | --- | --- | --- | --- | --- | --- |
|  | N | Minimum | Maximum | Mittelwert | | Standardabweichung |
|  | Statistik | Statistik | Statistik | Statistik | Standardfehler | Statistik |
| BMI | 14 | 18,3655 | 34,7209 | 21,783820 | 1,2382373 | 4,6330598 |
| Gültige Werte (Listenweise) | 14 |  |  |  |  |  |

**Geschlecht = 0, ZEITPUNKT = 1**

| **Deskriptive Statistik** | | | | | | |
| --- | --- | --- | --- | --- | --- | --- |
|  | N | Minimum | Maximum | Mittelwert | | Standardabweichung |
|  | Statistik | Statistik | Statistik | Statistik | Standardfehler | Statistik |
| BMI | 4 | 18,3655 | 19,9573 | 19,161379 | ,4595164 | ,9190328 |
| Gültige Werte (Listenweise) | 4 |  |  |  |  |  |

**Geschlecht = 0, ZEITPUNKT = 2**

| **Deskriptive Statistik** | | | | | | |
| --- | --- | --- | --- | --- | --- | --- |
|  | N | Minimum | Maximum | Mittelwert | | Standardabweichung |
|  | Statistik | Statistik | Statistik | Statistik | Standardfehler | Statistik |
| BMI | 6 | 19,0311 | 34,7209 | 24,454056 | 2,4405573 | 5,9781200 |
| Gültige Werte (Listenweise) | 6 |  |  |  |  |  |

**Geschlecht = 0, ZEITPUNKT = 3**

| **Deskriptive Statistik** | | | | | | |
| --- | --- | --- | --- | --- | --- | --- |
|  | N | Minimum | Maximum | Mittelwert | | Standardabweichung |
|  | Statistik | Statistik | Statistik | Statistik | Standardfehler | Statistik |
| BMI | 2 | 18,7783 | 23,1111 | 20,944728 | 2,1663832 | 3,0637285 |
| Gültige Werte (Listenweise) | 2 |  |  |  |  |  |

**Geschlecht = 0, ZEITPUNKT = 4**

| **Deskriptive Statistik** | | | | | | |
| --- | --- | --- | --- | --- | --- | --- |
|  | N | Minimum | Maximum | Mittelwert | | Standardabweichung |
|  | Statistik | Statistik | Statistik | Statistik | Standardfehler | Statistik |
| BMI | 6 | 18,4240 | 24,2382 | 20,987022 | 1,0834988 | 2,6540193 |
| Gültige Werte (Listenweise) | 6 |  |  |  |  |  |

**Geschlecht = 1, ZEITPUNKT = 0**

| **Deskriptive Statistik** | | | | | | |
| --- | --- | --- | --- | --- | --- | --- |
|  | N | Minimum | Maximum | Mittelwert | | Standardabweichung |
|  | Statistik | Statistik | Statistik | Statistik | Standardfehler | Statistik |
| BMI | 23 | 21,7993 | 34,3490 | 26,515395 | ,6325433 | 3,0335709 |
| Gültige Werte (Listenweise) | 23 |  |  |  |  |  |

**Geschlecht = 1, ZEITPUNKT = 1**

| **Deskriptive Statistik** | | | | | | |
| --- | --- | --- | --- | --- | --- | --- |
|  | N | Minimum | Maximum | Mittelwert | | Standardabweichung |
|  | Statistik | Statistik | Statistik | Statistik | Standardfehler | Statistik |
| BMI | 9 | 23,5102 | 34,3490 | 26,290551 | 1,1244252 | 3,3732756 |
| Gültige Werte (Listenweise) | 9 |  |  |  |  |  |

**Geschlecht = 1, ZEITPUNKT = 2**

| **Deskriptive Statistik** | | | | | | |
| --- | --- | --- | --- | --- | --- | --- |
|  | N | Minimum | Maximum | Mittelwert | | Standardabweichung |
|  | Statistik | Statistik | Statistik | Statistik | Standardfehler | Statistik |
| BMI | 7 | 21,7993 | 28,2933 | 25,773611 | ,9666037 | 2,5573930 |
| Gültige Werte (Listenweise) | 7 |  |  |  |  |  |

**Geschlecht = 1, ZEITPUNKT = 3**

| **Deskriptive Statistik** | | | | | | |
| --- | --- | --- | --- | --- | --- | --- |
|  | N | Minimum | Maximum | Mittelwert | | Standardabweichung |
|  | Statistik | Statistik | Statistik | Statistik | Standardfehler | Statistik |
| BMI | 8 | 22,7244 | 32,2831 | 27,358863 | 1,0505833 | 2,9714982 |
| Gültige Werte (Listenweise) | 8 |  |  |  |  |  |

**Geschlecht = 1, ZEITPUNKT = 4**

| **Deskriptive Statistik** | | | | | | |
| --- | --- | --- | --- | --- | --- | --- |
|  | N | Minimum | Maximum | Mittelwert | | Standardabweichung |
|  | Statistik | Statistik | Statistik | Statistik | Standardfehler | Statistik |
| BMI | 7 | 21,4619 | 29,5858 | 25,822382 | 1,1527366 | 3,0498544 |
| Gültige Werte (Listenweise) | 7 |  |  |  |  |  |
