# Supplementary material for: Intra-articular injection of bone marrow aspirate concentrate (mesenchymal stem cells) in KL grade III and IV knee osteoarthritis: 4 year results of 37 knees
Source: Sci Rep. 2024 Feb 1;14:2665. doi: 10.1038/s41598-024-51410-2 (PMC10834500; doi:10.1038/s41598-024-51410-2)
Supplement: Supplementary file 2 — Supplementary Information 2. [file 41598_2024_51410_MOESM2_ESM.docx]

95%iges Konfidenzintervall: Immer MITTELWERT – STANDARDFEHLER für Untergrenze und MITTELWERT + STANDARDFEHLER für Obergrenze

**IKDC – paired: Gesamt**

| **Deskriptive Statistik** | | | | | | |
| --- | --- | --- | --- | --- | --- | --- |
|  | N | Minimum | Maximum | Mittelwert | | Standardabweichung |
|  | Statistik | Statistik | Statistik | Statistik | Standardfehler | Statistik |
| t0_IKDC | 37 | 34,0 | 81,0 | 56,038 | 1,9667 | 11,9627 |
| t1_IKDC | 37 | 45,0 | 100,0 | 71,892 | 2,1851 | 13,2911 |
| Gültige Werte (Listenweise) | 37 |  |  |  |  |  |

- zB Konfidenzintervall für t0 ist 54,071 bis 58,005 um den Mittelwert von 56,04

**IKDC – paired: Getrennt nach Geschlecht**

**t0_Geschlecht = 0**

| **Deskriptive Statistik** | | | | | | |
| --- | --- | --- | --- | --- | --- | --- |
|  | N | Minimum | Maximum | Mittelwert | | Standardabweichung |
|  | Statistik | Statistik | Statistik | Statistik | Standardfehler | Statistik |
| t0_IKDC | 14 | 34,0 | 81,0 | 50,600 | 3,1314 | 11,7167 |
| t1_IKDC | 14 | 59,0 | 89,0 | 71,786 | 2,8865 | 10,8001 |
| Gültige Werte (Listenweise) | 14 |  |  |  |  |  |

**t0_Geschlecht = 1**

| **Deskriptive Statistik** | | | | | | |
| --- | --- | --- | --- | --- | --- | --- |
|  | N | Minimum | Maximum | Mittelwert | | Standardabweichung |
|  | Statistik | Statistik | Statistik | Statistik | Standardfehler | Statistik |
| t0_IKDC | 23 | 45,0 | 79,0 | 59,348 | 2,3105 | 11,0807 |
| t1_IKDC | 23 | 45,0 | 100,0 | 71,957 | 3,0937 | 14,8369 |
| Gültige Werte (Listenweise) | 23 |  |  |  |  |  |

**IKDC – unpaired: Gesamt**

| **Deskriptive Statistik** | | | | | | |
| --- | --- | --- | --- | --- | --- | --- |
|  | N | Minimum | Maximum | Mittelwert | | Standardabweichung |
|  | Statistik | Statistik | Statistik | Statistik | Standardfehler | Statistik |
| IKDC | 86 | 34,0 | 100,0 | 66,144 | 1,6789 | 15,5696 |
| Gültige Werte (Listenweise) | 86 |  |  |  |  |  |

**IKDC – unpaired: Getrennt nach Geschlecht**

**Geschlecht = 0**

| **Deskriptive Statistik** | | | | | | |
| --- | --- | --- | --- | --- | --- | --- |
|  | N | Minimum | Maximum | Mittelwert | | Standardabweichung |
|  | Statistik | Statistik | Statistik | Statistik | Standardfehler | Statistik |
| IKDC | 32 | 34,0 | 94,0 | 63,700 | 2,9573 | 16,7290 |
| Gültige Werte (Listenweise) | 32 |  |  |  |  |  |

**Geschlecht = 1**

| **Deskriptive Statistik** | | | | | | |
| --- | --- | --- | --- | --- | --- | --- |
|  | N | Minimum | Maximum | Mittelwert | | Standardabweichung |
|  | Statistik | Statistik | Statistik | Statistik | Standardfehler | Statistik |
| IKDC | 54 | 45,0 | 100,0 | 67,593 | 2,0154 | 14,8101 |
| Gültige Werte (Listenweise) | 54 |  |  |  |  |  |

**IKDC – unpaired: Getrennt nach Vorher_Nachher**

**Vorher_Nachher = 0**

| **Deskriptive Statistik** | | | | | | |
| --- | --- | --- | --- | --- | --- | --- |
|  | N | Minimum | Maximum | Mittelwert | | Standardabweichung |
|  | Statistik | Statistik | Statistik | Statistik | Standardfehler | Statistik |
| IKDC | 37 | 34,0 | 81,0 | 56,038 | 1,9667 | 11,9627 |
| Gültige Werte (Listenweise) | 37 |  |  |  |  |  |

**Vorher_Nachher = 1**

| **Deskriptive Statistik** | | | | | | |
| --- | --- | --- | --- | --- | --- | --- |
|  | N | Minimum | Maximum | Mittelwert | | Standardabweichung |
|  | Statistik | Statistik | Statistik | Statistik | Standardfehler | Statistik |
| IKDC | 49 | 45,0 | 100,0 | 73,776 | 1,9365 | 13,5558 |
| Gültige Werte (Listenweise) | 49 |  |  |  |  |  |

**IKDC – unpaired: Getrennt nach Zeitpunkt**

**ZEITPUNKT = 0**

| **Deskriptive Statistik** | | | | | | |
| --- | --- | --- | --- | --- | --- | --- |
|  | N | Minimum | Maximum | Mittelwert | | Standardabweichung |
|  | Statistik | Statistik | Statistik | Statistik | Standardfehler | Statistik |
| IKDC | 37 | 34,0 | 81,0 | 56,038 | 1,9667 | 11,9627 |
| Gültige Werte (Listenweise) | 37 |  |  |  |  |  |

**ZEITPUNKT = 1**

| **Deskriptive Statistik** | | | | | | |
| --- | --- | --- | --- | --- | --- | --- |
|  | N | Minimum | Maximum | Mittelwert | | Standardabweichung |
|  | Statistik | Statistik | Statistik | Statistik | Standardfehler | Statistik |
| IKDC | 13 | 45,0 | 97,0 | 67,385 | 4,5384 | 16,3633 |
| Gültige Werte (Listenweise) | 13 |  |  |  |  |  |

**ZEITPUNKT = 2**

| **Deskriptive Statistik** | | | | | | |
| --- | --- | --- | --- | --- | --- | --- |
|  | N | Minimum | Maximum | Mittelwert | | Standardabweichung |
|  | Statistik | Statistik | Statistik | Statistik | Standardfehler | Statistik |
| IKDC | 13 | 64,0 | 94,0 | 77,000 | 3,1785 | 11,4601 |
| Gültige Werte (Listenweise) | 13 |  |  |  |  |  |

**ZEITPUNKT = 3**

| **Deskriptive Statistik** | | | | | | |
| --- | --- | --- | --- | --- | --- | --- |
|  | N | Minimum | Maximum | Mittelwert | | Standardabweichung |
|  | Statistik | Statistik | Statistik | Statistik | Standardfehler | Statistik |
| IKDC | 10 | 54,0 | 100,0 | 76,300 | 4,8832 | 15,4420 |
| Gültige Werte (Listenweise) | 10 |  |  |  |  |  |

**ZEITPUNKT = 4**

| **Deskriptive Statistik** | | | | | | |
| --- | --- | --- | --- | --- | --- | --- |
|  | N | Minimum | Maximum | Mittelwert | | Standardabweichung |
|  | Statistik | Statistik | Statistik | Statistik | Standardfehler | Statistik |
| IKDC | 13 | 62,0 | 89,0 | 75,000 | 2,6985 | 9,7297 |
| Gültige Werte (Listenweise) | 13 |  |  |  |  |  |

**IKDC – unpaired: Getrennt nach Geschlecht und Vorher_Nachher**

**Geschlecht = 0, Vorher_Nachher = 0**

| **Deskriptive Statistik** | | | | | | |
| --- | --- | --- | --- | --- | --- | --- |
|  | N | Minimum | Maximum | Mittelwert | | Standardabweichung |
|  | Statistik | Statistik | Statistik | Statistik | Standardfehler | Statistik |
| IKDC | 14 | 34,0 | 81,0 | 50,600 | 3,1314 | 11,7167 |
| Gültige Werte (Listenweise) | 14 |  |  |  |  |  |

**Geschlecht = 0, Vorher_Nachher = 1**

| **Deskriptive Statistik** | | | | | | |
| --- | --- | --- | --- | --- | --- | --- |
|  | N | Minimum | Maximum | Mittelwert | | Standardabweichung |
|  | Statistik | Statistik | Statistik | Statistik | Standardfehler | Statistik |
| IKDC | 18 | 57,0 | 94,0 | 73,889 | 2,9260 | 12,4139 |
| Gültige Werte (Listenweise) | 18 |  |  |  |  |  |

**Geschlecht = 1, Vorher_Nachher = 0**

| **Deskriptive Statistik** | | | | | | |
| --- | --- | --- | --- | --- | --- | --- |
|  | N | Minimum | Maximum | Mittelwert | | Standardabweichung |
|  | Statistik | Statistik | Statistik | Statistik | Standardfehler | Statistik |
| IKDC | 23 | 45,0 | 79,0 | 59,348 | 2,3105 | 11,0807 |
| Gültige Werte (Listenweise) | 23 |  |  |  |  |  |

**Geschlecht = 1, Vorher_Nachher = 1**

| **Deskriptive Statistik** | | | | | | |
| --- | --- | --- | --- | --- | --- | --- |
|  | N | Minimum | Maximum | Mittelwert | | Standardabweichung |
|  | Statistik | Statistik | Statistik | Statistik | Standardfehler | Statistik |
| IKDC | 31 | 45,0 | 100,0 | 73,710 | 2,5821 | 14,3764 |
| Gültige Werte (Listenweise) | 31 |  |  |  |  |  |

**IKDC – unpaired: Getrennt nach Geschlecht und Zeitpunkt**

**Geschlecht = 0, ZEITPUNKT = 0**

| **Deskriptive Statistik** | | | | | | |
| --- | --- | --- | --- | --- | --- | --- |
|  | N | Minimum | Maximum | Mittelwert | | Standardabweichung |
|  | Statistik | Statistik | Statistik | Statistik | Standardfehler | Statistik |
| IKDC | 14 | 34,0 | 81,0 | 50,600 | 3,1314 | 11,7167 |
| Gültige Werte (Listenweise) | 14 |  |  |  |  |  |

**Geschlecht = 0, ZEITPUNKT = 1**

| **Deskriptive Statistik** | | | | | | |
| --- | --- | --- | --- | --- | --- | --- |
|  | N | Minimum | Maximum | Mittelwert | | Standardabweichung |
|  | Statistik | Statistik | Statistik | Statistik | Standardfehler | Statistik |
| IKDC | 4 | 59,0 | 63,0 | 61,000 | 1,1547 | 2,3094 |
| Gültige Werte (Listenweise) | 4 |  |  |  |  |  |

**Geschlecht = 0, ZEITPUNKT = 2**

| **Deskriptive Statistik** | | | | | | |
| --- | --- | --- | --- | --- | --- | --- |
|  | N | Minimum | Maximum | Mittelwert | | Standardabweichung |
|  | Statistik | Statistik | Statistik | Statistik | Standardfehler | Statistik |
| IKDC | 6 | 64,0 | 94,0 | 77,667 | 4,8351 | 11,8434 |
| Gültige Werte (Listenweise) | 6 |  |  |  |  |  |

**Geschlecht = 0, ZEITPUNKT = 3**

| **Deskriptive Statistik** | | | | | | |
| --- | --- | --- | --- | --- | --- | --- |
|  | N | Minimum | Maximum | Mittelwert | | Standardabweichung |
|  | Statistik | Statistik | Statistik | Statistik | Standardfehler | Statistik |
| IKDC | 2 | 57,0 | 85,0 | 71,000 | 14,0000 | 19,7990 |
| Gültige Werte (Listenweise) | 2 |  |  |  |  |  |

**Geschlecht = 0, ZEITPUNKT = 4**

| **Deskriptive Statistik** | | | | | | |
| --- | --- | --- | --- | --- | --- | --- |
|  | N | Minimum | Maximum | Mittelwert | | Standardabweichung |
|  | Statistik | Statistik | Statistik | Statistik | Standardfehler | Statistik |
| IKDC | 6 | 62,0 | 89,0 | 79,667 | 4,2557 | 10,4243 |
| Gültige Werte (Listenweise) | 6 |  |  |  |  |  |

**Geschlecht = 1, ZEITPUNKT = 0**

| **Deskriptive Statistik** | | | | | | |
| --- | --- | --- | --- | --- | --- | --- |
|  | N | Minimum | Maximum | Mittelwert | | Standardabweichung |
|  | Statistik | Statistik | Statistik | Statistik | Standardfehler | Statistik |
| IKDC | 23 | 45,0 | 79,0 | 59,348 | 2,3105 | 11,0807 |
| Gültige Werte (Listenweise) | 23 |  |  |  |  |  |

**Geschlecht = 1, ZEITPUNKT = 1**

| **Deskriptive Statistik** | | | | | | |
| --- | --- | --- | --- | --- | --- | --- |
|  | N | Minimum | Maximum | Mittelwert | | Standardabweichung |
|  | Statistik | Statistik | Statistik | Statistik | Standardfehler | Statistik |
| IKDC | 9 | 45,0 | 97,0 | 70,222 | 6,4135 | 19,2404 |
| Gültige Werte (Listenweise) | 9 |  |  |  |  |  |

**Geschlecht = 1, ZEITPUNKT = 2**

| **Deskriptive Statistik** | | | | | | |
| --- | --- | --- | --- | --- | --- | --- |
|  | N | Minimum | Maximum | Mittelwert | | Standardabweichung |
|  | Statistik | Statistik | Statistik | Statistik | Standardfehler | Statistik |
| IKDC | 7 | 64,0 | 93,0 | 76,429 | 4,5505 | 12,0396 |
| Gültige Werte (Listenweise) | 7 |  |  |  |  |  |

**Geschlecht = 1, ZEITPUNKT = 3**

| **Deskriptive Statistik** | | | | | | |
| --- | --- | --- | --- | --- | --- | --- |
|  | N | Minimum | Maximum | Mittelwert | | Standardabweichung |
|  | Statistik | Statistik | Statistik | Statistik | Standardfehler | Statistik |
| IKDC | 8 | 54,0 | 100,0 | 77,625 | 5,4835 | 15,5098 |
| Gültige Werte (Listenweise) | 8 |  |  |  |  |  |

**Geschlecht = 1, ZEITPUNKT = 4**

| **Deskriptive Statistik** | | | | | | |
| --- | --- | --- | --- | --- | --- | --- |
|  | N | Minimum | Maximum | Mittelwert | | Standardabweichung |
|  | Statistik | Statistik | Statistik | Statistik | Standardfehler | Statistik |
| IKDC | 7 | 62,0 | 80,0 | 71,000 | 2,8868 | 7,6376 |
| Gültige Werte (Listenweise) | 7 |  |  |  |  |  |

**Univariate Varianzanalyse: Vergleich zwischen Zeitpunkten und Geschlechter (unpaired)**

| **Zwischensubjektfaktoren** | | |
| --- | --- | --- |
|  | | N |
| ZEITPUNKT | 0 | 37 |
|  | 1 | 13 |
|  | 2 | 13 |
|  | 3 | 10 |
|  | 4 | 13 |
| Geschlecht | 0 | 32 |
|  | 1 | 54 |

| **Deskriptive Statistiken** | | | | |
| --- | --- | --- | --- | --- |
| Abhängige Variable: IKDC | | | | |
| ZEITPUNKT | Geschlecht | Mittelwert | Standardabweichung | N |
| 0 | 0 | 50,600 | 11,7167 | 14 |
|  | 1 | 59,348 | 11,0807 | 23 |
|  | Gesamt | 56,038 | 11,9627 | 37 |
| 1 | 0 | 61,000 | 2,3094 | 4 |
|  | 1 | 70,222 | 19,2404 | 9 |
|  | Gesamt | 67,385 | 16,3633 | 13 |
| 2 | 0 | 77,667 | 11,8434 | 6 |
|  | 1 | 76,429 | 12,0396 | 7 |
|  | Gesamt | 77,000 | 11,4601 | 13 |
| 3 | 0 | 71,000 | 19,7990 | 2 |
|  | 1 | 77,625 | 15,5098 | 8 |
|  | Gesamt | 76,300 | 15,4420 | 10 |
| 4 | 0 | 79,667 | 10,4243 | 6 |
|  | 1 | 71,000 | 7,6376 | 7 |
|  | Gesamt | 75,000 | 9,7297 | 13 |
| Gesamt | 0 | 63,700 | 16,7290 | 32 |
|  | 1 | 67,593 | 14,8101 | 54 |
|  | Gesamt | 66,144 | 15,5696 | 86 |

| **Tests der Zwischensubjekteffekte** | | | | | |
| --- | --- | --- | --- | --- | --- |
| Abhängige Variable: IKDC | | | | | |
| Quelle | Quadratsumme vom Typ III | df | Mittel der Quadrate | F | Sig. |
| Korrigiertes Modell | 8601,423 | 9 | 955,714 | 6,051 | ,000 |
| Konstanter Term | 280459,868 | 1 | 280459,868 | 1775,703 | ,000 |
| ZEITPUNKT | 7313,493 | 4 | 1828,373 | 11,576 | ,000 |
| Geschlecht | 125,463 | 1 | 125,463 | ,794 | ,376 |
| ZEITPUNKT * Geschlecht | 889,492 | 4 | 222,373 | 1,408 | ,239 |
| Fehler | 12003,669 | 76 | 157,943 |  |  |
| Gesamt | 396859,680 | 86 |  |  |  |
| Korrigierte Gesamtvariation | 20605,092 | 85 |  |  |  |

- Signifikanter Unteschied zwischen Zeitpunkten
- Keiner zwischen Geschlechtern und keine Wechselwirkung

**Geschätzte Randmittel: Details zum Unterschied zwischen Zeitpunkten**

| **Paarweise Vergleiche** | | | | | | |
| --- | --- | --- | --- | --- | --- | --- |
| Abhängige Variable: IKDC | | | | | | |
| (I)ZEITPUNKT | (J)ZEITPUNKT | Mittlere Differenz (I-J) | Standardfehler | Sig. | 95% Konfidenzintervall für die Differenz | |
|  |  |  |  |  | Untergrenze | Obergrenze |
| 0 | 1 | -10,637 | 4,335 | ,164 | -23,172 | 1,898 |
|  | 2 | -22,074 | 4,094 | ,000 | -33,910 | -10,237 |
|  | 3 | -19,339 | 5,405 | ,006 | -34,967 | -3,711 |
|  | 4 | -20,359 | 4,094 | ,000 | -32,196 | -8,523 |
| 1 | 0 | 10,637 | 4,335 | ,164 | -1,898 | 23,172 |
|  | 2 | -11,437 | 5,146 | ,292 | -26,315 | 3,442 |
|  | 3 | -8,701 | 6,240 | 1,000 | -26,743 | 9,340 |
|  | 4 | -9,722 | 5,146 | ,627 | -24,601 | 5,156 |
| 2 | 0 | 22,074 | 4,094 | ,000 | 10,237 | 33,910 |
|  | 1 | 11,437 | 5,146 | ,292 | -3,442 | 26,315 |
|  | 3 | 2,735 | 6,075 | 1,000 | -14,828 | 20,298 |
|  | 4 | 1,714 | 4,944 | 1,000 | -12,580 | 16,009 |
| 3 | 0 | 19,339 | 5,405 | ,006 | 3,711 | 34,967 |
|  | 1 | 8,701 | 6,240 | 1,000 | -9,340 | 26,743 |
|  | 2 | -2,735 | 6,075 | 1,000 | -20,298 | 14,828 |
|  | 4 | -1,021 | 6,075 | 1,000 | -18,584 | 16,543 |
| 4 | 0 | 20,359 | 4,094 | ,000 | 8,523 | 32,196 |
|  | 1 | 9,722 | 5,146 | ,627 | -5,156 | 24,601 |
|  | 2 | -1,714 | 4,944 | 1,000 | -16,009 | 12,580 |
|  | 3 | 1,021 | 6,075 | 1,000 | -16,543 | 18,584 |

- Zeitpunkt 0 unterscheidet sich nicht von 1, aber von 2 bis 4.
- Alle anderen Zeitpunkte unterscheiden sich nicht.
