# Supplementary material for: Intra-articular injection of bone marrow aspirate concentrate (mesenchymal stem cells) in KL grade III and IV knee osteoarthritis: 4 year results of 37 knees
Source: Sci Rep. 2024 Feb 1;14:2665. doi: 10.1038/s41598-024-51410-2 (PMC10834500; doi:10.1038/s41598-024-51410-2)
Supplement: Supplementary file 3 — Supplementary Information 3. [file 41598_2024_51410_MOESM3_ESM.docx]

**Paired T-Test**

| **Statistik bei gepaarten Stichproben** | | | | | |
| --- | --- | --- | --- | --- | --- |
|  | | Mittelwert | N | Standardabweichung | Standardfehler des Mittelwertes |
| Paaren 1 | t0_IKDC | 56,038 | 37 | 11,9627 | 1,9667 |
|  | t1_IKDC | 71,892 | 37 | 13,2911 | 2,1851 |

| **Korrelationen bei gepaarten Stichproben** | | | | |
| --- | --- | --- | --- | --- |
|  | | N | Korrelation | Signifikanz |
| Paaren 1 | t0_IKDC & t1_IKDC | 37 | ,641 | ,000 |

P<.001

Wer vorher niedriger ist ist nachher niedriger, wer vorher höher ist, ist nachher höher.

| **Test bei gepaarten Stichproben** | | | | | | | | | |
| --- | --- | --- | --- | --- | --- | --- | --- | --- | --- |
|  | | Gepaarte Differenzen | | | | | T | df | Sig. (2-seitig) |
|  |  | Mittelwert | Standardabweichung | Standardfehler des Mittelwertes | 95% Konfidenzintervall der Differenz | |  |  |  |
|  |  |  |  |  | Untere | Obere |  |  |  |
| Paaren 1 | t0_IKDC - t1_IKDC | -15,8541 | 10,7671 | 1,7701 | -19,4440 | -12,2641 | -8,957 | 36 | ,000 |

Signifigant besser nachehr

**Allgemeines Lineares Modell**

| **Innersubjektfaktoren** | |
| --- | --- |
| Maß: MASS_1 | |
| Faktor1 | Abhängige Variable |
| 1 | t0_IKDC |
| 2 | t1_IKDC |

| **Deskriptive Statistiken** | | | | |
| --- | --- | --- | --- | --- |
|  | t1_Geschlecht | Mittelwert | Standardabweichung | N |
| t0_IKDC | 0 | 50,600 | 11,7167 | 14 |
|  | 1 | 59,348 | 11,0807 | 23 |
|  | Gesamt | 56,038 | 11,9627 | 37 |
| t1_IKDC | 0 | 71,786 | 10,8001 | 14 |
|  | 1 | 71,957 | 14,8369 | 23 |
|  | Gesamt | 71,892 | 13,2911 | 37 |

Geschlecht 0 = weiblich, 1=männlihc ??

Frauen starten shclechter

| **Tests der Innersubjektkontraste** | | | | | | |
| --- | --- | --- | --- | --- | --- | --- |
| Maß: MASS_1 | | | | | | |
| Quelle | Faktor1 | Quadratsumme vom Typ III | df | Mittel der Quadrate | F | Sig. |
| Faktor1 | Linear | 4969,514 | 1 | 4969,514 | 98,454 | ,000 |
| Faktor1 * t1_Geschlecht | Linear | 320,108 | 1 | 320,108 | 6,342 | ,017 |
| Fehler(Faktor1) | Linear | 1766,638 | 35 | 50,475 |  |  |

- Hier mit signifikanter Wechselwirkung, Details siehe unten

| **Tests der Zwischensubjekteffekte** | | | | | |
| --- | --- | --- | --- | --- | --- |
| Maß: MASS_1  Transformierte Variable: Mittel | | | | | |
| Quelle | Quadratsumme vom Typ III | df | Mittel der Quadrate | F | Sig. |
| Konstanter Term | 280047,088 | 1 | 280047,088 | 1079,651 | ,000 |
| t1_Geschlecht | 346,115 | 1 | 346,115 | 1,334 | ,256 |
| Fehler | 9078,533 | 35 | 259,387 |  |  |

**Geschätzte Randmittel**

**1. t1_Geschlecht * Faktor1**

| **Paarweise Vergleiche** | | | | | | | |
| --- | --- | --- | --- | --- | --- | --- | --- |
| Maß: MASS_1 | | | | | | | |
| Faktor1 | (I)t1_Geschlecht | (J)t1_Geschlecht | Mittlere Differenz (I-J) | Standardfehler | Sig. | 95% Konfidenzintervall für die Differenz | |
|  |  |  |  |  |  | Untergrenze | Obergrenze |
| 1 | 0 | 1 | -8,748 | 3,838 | ,029 | -16,539 | -,957 |
|  | 1 | 0 | 8,748 | 3,838 | ,029 | ,957 | 16,539 |
| 2 | 0 | 1 | -,171 | 4,569 | ,970 | -9,447 | 9,105 |
|  | 1 | 0 | ,171 | 4,569 | ,970 | -9,105 | 9,447 |

- Bei Vorher signifikanter Geschlechtsunterschied mit p=,029; bei nachher ganz klar nicht (p=,970)
- Vorher: Frauen vorher signifikant schlechter als Männer (50 versus 59, p<0.29), nachher nicht mehr. (72, p<.970). Beide verbessern sich unabhängig.
- Das ist die Ursache der Wechselwirkung, dass eben Geschlechtsunterschied abhängig von Zeitpunkt

Vorher / nachher

Kein genereller geschlechtsunterschied

**2. t1_Geschlecht * Faktor1**

| **Paarweise Vergleiche** | | | | | | | |
| --- | --- | --- | --- | --- | --- | --- | --- |
| Maß: MASS_1 | | | | | | | |
| t1_Geschlecht | (I)Faktor1 | (J)Faktor1 | Mittlere Differenz (I-J) | Standardfehler | Sig. | 95% Konfidenzintervall für die Differenz | |
|  |  |  |  |  |  | Untergrenze | Obergrenze |
| 0 | 1 | 2 | -21,186 | 2,685 | ,000 | -26,637 | -15,734 |
|  | 2 | 1 | 21,186 | 2,685 | ,000 | 15,734 | 26,637 |
| 1 | 1 | 2 | -12,609 | 2,095 | ,000 | -16,862 | -8,356 |
|  | 2 | 1 | 12,609 | 2,095 | ,000 | 8,356 | 16,862 |

- In beiden Geschlechtern gibt es signifikanten vorher-Nachher-Unterschied, das ist das eigentlich interessante und daher ist die WW wohl wenig interessant…

Frauen und männer immer besser v/np<.001

**Allgemeines Lineares Modell: Nun mit Kovariate TagepostOP; Ergebnisse wie oben…**

Bestätigt in der Varianzanalyse (Akllgemeines lineares Modell) mit Zeitpunkt und Geschlecht.

| **Innersubjektfaktoren** | |
| --- | --- |
| Maß: MASS_1 | |
| Faktor1 | Abhängige Variable |
| 1 | t0_IKDC |
| 2 | t1_IKDC |

| **Deskriptive Statistiken** | | | | |
| --- | --- | --- | --- | --- |
|  | t1_Geschlecht | Mittelwert | Standardabweichung | N |
| t0_IKDC | 0 | 50,600 | 11,7167 | 14 |
|  | 1 | 59,348 | 11,0807 | 23 |
|  | Gesamt | 56,038 | 11,9627 | 37 |
| t1_IKDC | 0 | 71,786 | 10,8001 | 14 |
|  | 1 | 71,957 | 14,8369 | 23 |
|  | Gesamt | 71,892 | 13,2911 | 37 |

| **Tests der Innersubjektkontraste** | | | | | | |
| --- | --- | --- | --- | --- | --- | --- |
| Maß: MASS_1 | | | | | | |
| Quelle | Faktor1 | Quadratsumme vom Typ III | df | Mittel der Quadrate | F | Sig. |
| Faktor1 | Linear | 1376,278 | 1 | 1376,278 | 26,492 | ,000 |
| Faktor1 * t1_Tagepostop | Linear | ,306 | 1 | ,306 | ,006 | ,939 |
| Faktor1 * t1_Geschlecht | Linear | 319,801 | 1 | 319,801 | 6,156 | ,018 |
| Fehler(Faktor1) | Linear | 1766,332 | 34 | 51,951 |  |  |

| **Tests der Zwischensubjekteffekte** | | | | | |
| --- | --- | --- | --- | --- | --- |
| Maß: MASS_1  Transformierte Variable: Mittel | | | | | |
| Quelle | Quadratsumme vom Typ III | df | Mittel der Quadrate | F | Sig. |
| Konstanter Term | 63561,928 | 1 | 63561,928 | 275,975 | ,000 |
| t1_Tagepostop | 1247,726 | 1 | 1247,726 | 5,417 | ,026 |
| t1_Geschlecht | 363,137 | 1 | 363,137 | 1,577 | ,218 |
| Fehler | 7830,807 | 34 | 230,318 |  |  |

Nicht darauf eingehen, inhaltlich kein Mehrwert.

**Geschätzte Randmittel**

**1. t1_Geschlecht * Faktor1**

| **Paarweise Vergleiche** | | | | | | | |
| --- | --- | --- | --- | --- | --- | --- | --- |
| Maß: MASS_1 | | | | | | | |
| Faktor1 | (I)t1_Geschlecht | (J)t1_Geschlecht | Mittlere Differenz (I-J) | Standardfehler | Sig. | 95% Konfidenzintervall für die Differenz | |
|  |  |  |  |  |  | Untergrenze | Obergrenze |
| 1 | 0 | 1 | -8,855 | 3,622 | ,020 | -16,216 | -1,494 |
|  | 1 | 0 | 8,855 | 3,622 | ,020 | 1,494 | 16,216 |
| 2 | 0 | 1 | -,281 | 4,395 | ,949 | -9,214 | 8,651 |
|  | 1 | 0 | ,281 | 4,395 | ,949 | -8,651 | 9,214 |

**2. t1_Geschlecht * Faktor1**

| **Paarweise Vergleiche** | | | | | | | |
| --- | --- | --- | --- | --- | --- | --- | --- |
| Maß: MASS_1 | | | | | | | |
| t1_Geschlecht | (I)Faktor1 | (J)Faktor1 | Mittlere Differenz (I-J) | Standardfehler | Sig. | 95% Konfidenzintervall für die Differenz | |
|  |  |  |  |  |  | Untergrenze | Obergrenze |
| 0 | 1 | 2 | -21,184 | 2,724 | ,000 | -26,720 | -15,647 |
|  | 2 | 1 | 21,184 | 2,724 | ,000 | 15,647 | 26,720 |
| 1 | 1 | 2 | -12,610 | 2,126 | ,000 | -16,930 | -8,290 |
|  | 2 | 1 | 12,610 | 2,126 | ,000 | 8,290 | 16,930 |

Kovariate...zur Kontrolle.

Berücksichtigung von tagen ändert nichts am Ergebnis.
