# Supplementary material for: Intra-articular injection of bone marrow aspirate concentrate (mesenchymal stem cells) in KL grade III and IV knee osteoarthritis: 4 year results of 37 knees
Source: Sci Rep. 2024 Feb 1;14:2665. doi: 10.1038/s41598-024-51410-2 (PMC10834500; doi:10.1038/s41598-024-51410-2)
Supplement: Supplementary file 4 — Supplementary Information 4. [file 41598_2024_51410_MOESM4_ESM.docx]

**Korrelationen**

| **Korrelationen** | | | | | |
| --- | --- | --- | --- | --- | --- |
|  | | IKDC | Größe in cm | Gewicht in kg | BMI |
| IKDC | Korrelation nach Pearson | 1 | -,056 | -,149 | -,157 |
|  | Signifikanz (2-seitig) |  | ,611 | ,172 | ,149 |
|  | N | 86 | 86 | 86 | 86 |
| Größe in cm | Korrelation nach Pearson | -,056 | 1 | ,830 | ,500 |
|  | Signifikanz (2-seitig) | ,611 |  | ,000 | ,000 |
|  | N | 86 | 86 | 86 | 86 |
| Gewicht in kg | Korrelation nach Pearson | -,149 | ,830 | 1 | ,894 |
|  | Signifikanz (2-seitig) | ,172 | ,000 |  | ,000 |
|  | N | 86 | 86 | 86 | 86 |
| BMI | Korrelation nach Pearson | -,157 | ,500 | ,894 | 1 |
|  | Signifikanz (2-seitig) | ,149 | ,000 | ,000 |  |
|  | N | 86 | 86 | 86 | 86 |

- Da hier (und im Folgenden auch getrennt nach Vorher-Nachher) keine potenzielle Kovariate signifikant, am Ende auch keine Analyse mit Kovariaten…

**Nichtparametrische Korrelationen**

| **Korrelationen** | | | | |
| --- | --- | --- | --- | --- |
|  | | | IKDC | ZEITPUNKT |
| Spearman-Rho | IKDC | Korrelationskoeffizient | 1,000 | ,562 |
|  |  | Sig. (2-seitig) | . | ,000 |
|  |  | N | 86 | 86 |
|  | ZEITPUNKT | Korrelationskoeffizient | ,562 | 1,000 |
|  |  | Sig. (2-seitig) | ,000 | . |
|  |  | N | 86 | 86 |

**Korrelationen**

**Vorher_Nachher = 0**

| **Korrelationen** | | | | | |
| --- | --- | --- | --- | --- | --- |
|  | | IKDC | Größe in cm | Gewicht in kg | BMI |
| IKDC | Korrelation nach Pearson | 1 | ,101 | -,023 | -,086 |
|  | Signifikanz (2-seitig) |  | ,550 | ,890 | ,614 |
|  | N | 37 | 37 | 37 | 37 |
| Größe in cm | Korrelation nach Pearson | ,101 | 1 | ,824 | ,488 |
|  | Signifikanz (2-seitig) | ,550 |  | ,000 | ,002 |
|  | N | 37 | 37 | 37 | 37 |
| Gewicht in kg | Korrelation nach Pearson | -,023 | ,824 | 1 | ,893 |
|  | Signifikanz (2-seitig) | ,890 | ,000 |  | ,000 |
|  | N | 37 | 37 | 37 | 37 |
| BMI | Korrelation nach Pearson | -,086 | ,488 | ,893 | 1 |
|  | Signifikanz (2-seitig) | ,614 | ,002 | ,000 |  |
|  | N | 37 | 37 | 37 | 37 |

**Vorher_Nachher = 1**

Tendentiell schwache Korrelatoin zwischen BMI (.077) und gewicht (.056) und IKDC.....NACHHER, nicht vorher. Daher keine Covariate, weil keil relevanter Einfluss.

| **Korrelationen** | | | | | |
| --- | --- | --- | --- | --- | --- |
|  | | IKDC | Größe in cm | Gewicht in kg | BMI |
| IKDC | Korrelation nach Pearson | 1 | -,165 | -,275 | -,255 |
|  | Signifikanz (2-seitig) |  | ,257 | ,056 | ,077 |
|  | N | 49 | 49 | 49 | 49 |
| Größe in cm | Korrelation nach Pearson | -,165 | 1 | ,835 | ,510 |
|  | Signifikanz (2-seitig) | ,257 |  | ,000 | ,000 |
|  | N | 49 | 49 | 49 | 49 |
| Gewicht in kg | Korrelation nach Pearson | -,275 | ,835 | 1 | ,895 |
|  | Signifikanz (2-seitig) | ,056 | ,000 |  | ,000 |
|  | N | 49 | 49 | 49 | 49 |
| BMI | Korrelation nach Pearson | -,255 | ,510 | ,895 | 1 |
|  | Signifikanz (2-seitig) | ,077 | ,000 | ,000 |  |
|  | N | 49 | 49 | 49 | 49 |

**Nichtparametrische Korrelationen**

**Vorher_Nachher = 1**

Keine Korrelatoin zwischen BMI, Größe, gewicht.....vorher

| **Korrelationen** | | | | |
| --- | --- | --- | --- | --- |
|  | | | IKDC | ZEITPUNKT |
| Spearman-Rho | IKDC | Korrelationskoeffizient | 1,000 | ,162 |
|  |  | Sig. (2-seitig) | . | ,266 |
|  |  | N | 49 | 49 |
|  | ZEITPUNKT | Korrelationskoeffizient | ,162 | 1,000 |
|  |  | Sig. (2-seitig) | ,266 | . |
|  |  | N | 49 | 49 |

**Nichtparametrische Tests**

**Vorher_Nachher = 0**

| **Kolmogorov-Smirnov-Anpassungstest** | | |
| --- | --- | --- |
|  | | IKDC |
| N | | 37 |
| Parameter der Normalverteilung | Mittelwert | 56,038 |
|  | Standardabweichung | 11,9627 |
| Extremste Differenzen | Absolut | ,126 |
|  | Positiv | ,126 |
|  | Negativ | -,084 |
| Kolmogorov-Smirnov-Z | | ,764 |
| Asymptotische Signifikanz (2-seitig) | | ,604 |

Normalverteilt vorher + nachher

**Vorher_Nachher = 1**

| **Kolmogorov-Smirnov-Anpassungstest** | | |
| --- | --- | --- |
|  | | IKDC |
| N | | 49 |
| Parameter der Normalverteilung | Mittelwert | 73,776 |
|  | Standardabweichung | 13,5558 |
| Extremste Differenzen | Absolut | ,112 |
|  | Positiv | ,112 |
|  | Negativ | -,079 |
| Kolmogorov-Smirnov-Z | | ,781 |
| Asymptotische Signifikanz (2-seitig) | | ,576 |

Normalverteilt vorher + nachher

**T-Test: Vorher-Nachher-Vergleich**

| **Gruppenstatistiken** | | | | | |
| --- | --- | --- | --- | --- | --- |
|  | Vorher_Nachher | N | Mittelwert | Standardabweichung | Standardfehler des Mittelwertes |
| IKDC | 0 | 37 | 56,038 | 11,9627 | 1,9667 |
|  | 1 | 49 | 73,776 | 13,5558 | 1,9365 |

| **Test bei unabhängigen Stichproben** | | | | | | | | | | |
| --- | --- | --- | --- | --- | --- | --- | --- | --- | --- | --- |
|  | | Levene-Test der Varianzgleichheit | | T-Test für die Mittelwertgleichheit | | | | | | |
|  |  | F | Signifikanz | T | df | Sig. (2-seitig) | Mittlere Differenz | Standardfehler der Differenz | 95% Konfidenzintervall der Differenz | |
|  |  |  |  |  |  |  |  |  | Untere | Obere |
| IKDC | Varianzen sind gleich | 1,006 | ,319 | **-6,315** | **84** | **,000** | -17,7377 | 2,8090 | -23,3236 | -12,1517 |
|  |  |  |  |  |  |  |  |  |  |  |

- Signifikanter Unterschied, Wert von 56 auf 73,8 gestiegen

Über alle vorher versus alle nachher

Paired: 56 auf 72

...bei manchen gab es auch mehrewre nachher ergbenisse...daher unpaired auch...ident...

**Univariate Varianzanalyse: Vergleich inkl. Geschlecht**

| **Zwischensubjektfaktoren** | | |
| --- | --- | --- |
|  | | N |
| Vorher_Nachher | 0 | 37 |
|  | 1 | 49 |
| Geschlecht | 0 | 32 |
|  | 1 | 54 |

| **Deskriptive Statistiken** | | | | |
| --- | --- | --- | --- | --- |
| Abhängige Variable: IKDC | | | | |
| Vorher_Nachher | Geschlecht | Mittelwert | Standardabweichung | N |
| 0 | 0 | 50,600 | 11,7167 | 14 |
|  | 1 | 59,348 | 11,0807 | 23 |
|  | Gesamt | 56,038 | 11,9627 | 37 |
| 1 | 0 | 73,889 | 12,4139 | 18 |
|  | 1 | 73,710 | 14,3764 | 31 |
|  | Gesamt | 73,776 | 13,5558 | 49 |
| Gesamt | 0 | 63,700 | 16,7290 | 32 |
|  | 1 | 67,593 | 14,8101 | 54 |
|  | Gesamt | 66,144 | 15,5696 | 86 |

| **Levene-Test auf Gleichheit der Fehlervarianzen** | | | |
| --- | --- | --- | --- |
| Abhängige Variable: IKDC | | | |
| F | df1 | df2 | Sig. |
| ,837 | 3 | 82 | ,477 |

| **Tests der Zwischensubjekteffekte** | | | | | |
| --- | --- | --- | --- | --- | --- |
| Abhängige Variable: IKDC | | | | | |
| Quelle | Quadratsumme vom Typ III | df | Mittel der Quadrate | F | Sig. |
| Korrigiertes Modell | 7299,070 | 3 | 2433,023 | 14,994 | ,000 |
| Konstanter Term | 327200,060 | 1 | 327200,060 | 2016,411 | ,000 |
| Vorher_Nachher | 6992,774 | 1 | 6992,774 | 43,094 | ,000 |
| Geschlecht | 362,179 | 1 | 362,179 | 2,232 | ,139 |
| Vorher_Nachher * Geschlecht | 393,113 | 1 | 393,113 | 2,423 | ,123 |
| Fehler | 13306,022 | 82 | 162,269 |  |  |
| Gesamt | 396859,680 | 86 |  |  |  |
| Korrigierte Gesamtvariation | 20605,092 | 85 |  |  |  |

- Vorher-Nachher signifikant, Rest nicht
