# Supplementary material for: Intra-articular injection of bone marrow aspirate concentrate (mesenchymal stem cells) in KL grade III and IV knee osteoarthritis: 4 year results of 37 knees
Source: Sci Rep. 2024 Feb 1;14:2665. doi: 10.1038/s41598-024-51410-2 (PMC10834500; doi:10.1038/s41598-024-51410-2)
Supplement: Supplementary file 5 — Supplementary Information 5. [file 41598_2024_51410_MOESM5_ESM.docx]

**T-Test**

| **Statistik bei gepaarten Stichproben** | | | | | |
| --- | --- | --- | --- | --- | --- |
|  | | Mittelwert | N | Standardabweichung | Standardfehler des Mittelwertes |
| Paaren 1 | t0_SF36_allgemein | 3,000 | 37 | ,6236 | ,1025 |
|  | t1_SF36_allgemein | 2,946 | 37 | ,7050 | ,1159 |

| **Korrelationen bei gepaarten Stichproben** | | | | |
| --- | --- | --- | --- | --- |
|  | | N | Korrelation | Signifikanz |
| Paaren 1 | t0_SF36_allgemein & t1_SF36_allgemein | 37 | ,632 | ,000 |

| **Test bei gepaarten Stichproben** | | | | | | | | | |
| --- | --- | --- | --- | --- | --- | --- | --- | --- | --- |
|  | | Gepaarte Differenzen | | | | | T | df | Sig. (2-seitig) |
|  |  | Mittelwert | Standardabweichung | Standardfehler des Mittelwertes | 95% Konfidenzintervall der Differenz | |  |  |  |
|  |  |  |  |  | Untere | Obere |  |  |  |
| Paaren 1 | t0_SF36_allgemein - t1_SF36_allgemein | ,0541 | ,5747 | ,0945 | -,1376 | ,2457 | ,572 | 36 | ,571 |

- Kein Unterschied zwischen Vorher und Nachher (letzter Zeitpunkt)

**Allgemeines Lineares Modell**

| **Innersubjektfaktoren** | |
| --- | --- |
| Maß: MASS_1 | |
| Faktor1 | Abhängige Variable |
| 1 | t0_SF36_allgemein |
| 2 | t1_SF36_allgemein |

| **Zwischensubjektfaktoren** | | |
| --- | --- | --- |
|  | | N |
| t1_Geschlecht | 0 | 14 |
|  | 1 | 23 |

| **Deskriptive Statistiken** | | | | |
| --- | --- | --- | --- | --- |
|  | t1_Geschlecht | Mittelwert | Standardabweichung | N |
| t0_SF36_allgemein | 0 | 2,786 | ,6993 | 14 |
|  | 1 | 3,130 | ,5481 | 23 |
|  | Gesamt | 3,000 | ,6236 | 37 |
| t1_SF36_allgemein | 0 | 2,929 | ,6157 | 14 |
|  | 1 | 2,957 | ,7674 | 23 |
|  | Gesamt | 2,946 | ,7050 | 37 |

| **Tests der Innersubjektkontraste** | | | | | | |
| --- | --- | --- | --- | --- | --- | --- |
| Maß: MASS_1 | | | | | | |
| Quelle | Faktor1 | Quadratsumme vom Typ III | df | Mittel der Quadrate | F | Sig. |
| Faktor1 | Linear | ,004 | 1 | ,004 | ,027 | ,871 |
| Faktor1 * t1_Geschlecht | Linear | ,437 | 1 | ,437 | 2,774 | ,105 |
| Fehler(Faktor1) | Linear | 5,509 | 35 | ,157 |  |  |

| **Tests der Zwischensubjekteffekte** | | | | | |
| --- | --- | --- | --- | --- | --- |
| Maß: MASS_1  Transformierte Variable: Mittel | | | | | |
| Quelle | Quadratsumme vom Typ III | df | Mittel der Quadrate | F | Sig. |
| Konstanter Term | 606,010 | 1 | 606,010 | 836,977 | ,000 |
| t1_Geschlecht | ,604 | 1 | ,604 | ,835 | ,367 |
| Fehler | 25,342 | 35 | ,724 |  |  |

- Bestätigt t-Test: Kein Vorher-Nachher-Unterschied
- Auch kein Geschlechtsunterschied und keine Wechselwirkung
- T-test alleine reicht…und stabiler als ANOVA, weil diese Variable eigentlich ordinalskaliert ist…

**Allgemeines Lineares Modell: Mit TagepostOP als Kovariate**

| **Innersubjektfaktoren** | |
| --- | --- |
| Maß: MASS_1 | |
| Faktor1 | Abhängige Variable |
| 1 | t0_SF36_allgemein |
| 2 | t1_SF36_allgemein |

| **Zwischensubjektfaktoren** | | |
| --- | --- | --- |
|  | | N |
| t1_Geschlecht | 0 | 14 |
|  | 1 | 23 |

| **Deskriptive Statistiken** | | | | |
| --- | --- | --- | --- | --- |
|  | t1_Geschlecht | Mittelwert | Standardabweichung | N |
| t0_SF36_allgemein | 0 | 2,786 | ,6993 | 14 |
|  | 1 | 3,130 | ,5481 | 23 |
|  | Gesamt | 3,000 | ,6236 | 37 |
| t1_SF36_allgemein | 0 | 2,929 | ,6157 | 14 |
|  | 1 | 2,957 | ,7674 | 23 |
|  | Gesamt | 2,946 | ,7050 | 37 |

| **Tests der Innersubjektkontraste** | | | | | | |
| --- | --- | --- | --- | --- | --- | --- |
| Maß: MASS_1 | | | | | | |
| Quelle | Faktor1 | Quadratsumme vom Typ III | df | Mittel der Quadrate | F | Sig. |
| Faktor1 | Linear | ,068 | 1 | ,068 | ,427 | ,518 |
| Faktor1 * t1_Tagepostop | Linear | ,121 | 1 | ,121 | ,766 | ,388 |
| Faktor1 * t1_Geschlecht | Linear | ,442 | 1 | ,442 | 2,792 | ,104 |
| Fehler(Faktor1) | Linear | 5,388 | 34 | ,158 |  |  |

| **Tests der Zwischensubjekteffekte** | | | | | |
| --- | --- | --- | --- | --- | --- |
| Maß: MASS_1  Transformierte Variable: Mittel | | | | | |
| Quelle | Quadratsumme vom Typ III | df | Mittel der Quadrate | F | Sig. |
| Konstanter Term | 176,880 | 1 | 176,880 | 237,744 | ,000 |
| t1_Tagepostop | ,046 | 1 | ,046 | ,062 | ,805 |
| t1_Geschlecht | ,600 | 1 | ,600 | ,806 | ,375 |
| Fehler | 25,296 | 34 | ,744 |  |  |

- Bestätigt t-Test: Kein Vorher-Nachher-Unterschied
- Auch kein Geschlechtsunterschied und keine Wechselwirkung
- T-test alleine reicht..

**T-Test**

| **Statistik bei gepaarten Stichproben** | | | | | |
| --- | --- | --- | --- | --- | --- |
|  | | Mittelwert | N | Standardabweichung | Standardfehler des Mittelwertes |
| Paaren 1 | t0_SF36_derzeitig_im_Vergleich | 3,027 | 37 | ,6866 | ,1129 |
|  | t1_SF36_derzeitig_im_Vergleich | 3,108 | 37 | ,8751 | ,1439 |

| **Korrelationen bei gepaarten Stichproben** | | | | |
| --- | --- | --- | --- | --- |
|  | | N | Korrelation | Signifikanz |
| Paaren 1 | t0_SF36_derzeitig_im_Vergleich & t1_SF36_derzeitig_im_Vergleich | 37 | ,272 | ,103 |

| **Test bei gepaarten Stichproben** | | | | | | | | | |
| --- | --- | --- | --- | --- | --- | --- | --- | --- | --- |
|  | | Gepaarte Differenzen | | | | | T | df | Sig. (2-seitig) |
|  |  | Mittelwert | Standardabweichung | Standardfehler des Mittelwertes | 95% Konfidenzintervall der Differenz | |  |  |  |
|  |  |  |  |  | Untere | Obere |  |  |  |
| Paaren 1 | t0_SF36_derzeitig_im_Vergleich - t1_SF36_derzeitig_im_Vergleich | -,0811 | ,9539 | ,1568 | -,3991 | ,2370 | -,517 | 36 | ,608 |

- Kein Unterschied zwischen Vorher und Nachher (letzter Zeitpunkt)

**Allgemeines Lineares Modell**

| **Innersubjektfaktoren** | |
| --- | --- |
| Maß: MASS_1 | |
| Faktor1 | Abhängige Variable |
| 1 | t0_SF36_derzeitig_im_Vergleich |
| 2 | t1_SF36_derzeitig_im_Vergleich |

| **Zwischensubjektfaktoren** | | |
| --- | --- | --- |
|  | | N |
| t1_Geschlecht | 0 | 14 |
|  | 1 | 23 |

| **Deskriptive Statistiken** | | | | |
| --- | --- | --- | --- | --- |
|  | t1_Geschlecht | Mittelwert | Standardabweichung | N |
| t0_SF36_derzeitig_im_Vergleich | 0 | 2,786 | ,5789 | 14 |
|  | 1 | 3,174 | ,7168 | 23 |
|  | Gesamt | 3,027 | ,6866 | 37 |
| t1_SF36_derzeitig_im_Vergleich | 0 | 3,000 | ,6794 | 14 |
|  | 1 | 3,174 | ,9841 | 23 |
|  | Gesamt | 3,108 | ,8751 | 37 |

| **Tests der Innersubjektkontraste** | | | | | | |
| --- | --- | --- | --- | --- | --- | --- |
| Maß: MASS_1 | | | | | | |
| Quelle | Faktor1 | Quadratsumme vom Typ III | df | Mittel der Quadrate | F | Sig. |
| Faktor1 | Linear | ,200 | 1 | ,200 | ,432 | ,515 |
| Faktor1 * t1_Geschlecht | Linear | ,200 | 1 | ,200 | ,432 | ,515 |
| Fehler(Faktor1) | Linear | 16,179 | 35 | ,462 |  |  |

| **Tests der Zwischensubjekteffekte** | | | | | |
| --- | --- | --- | --- | --- | --- |
| Maß: MASS_1  Transformierte Variable: Mittel | | | | | |
| Quelle | Quadratsumme vom Typ III | df | Mittel der Quadrate | F | Sig. |
| Konstanter Term | 640,618 | 1 | 640,618 | 837,026 | ,000 |
| t1_Geschlecht | 1,375 | 1 | 1,375 | 1,796 | ,189 |
| Fehler | 26,787 | 35 | ,765 |  |  |

- Bestätigt t-Test: Kein Vorher-Nachher-Unterschied
- Auch kein Geschlechtsunterschied und keine Wechselwirkung
- T-test alleine reicht…und stabiler als ANOVA, weil diese Variable eigentlich ordinalskaliert ist…

**Allgemeines Lineares Modell: Mit TagepostOP als Kovariate**

| **Innersubjektfaktoren** | |
| --- | --- |
| Maß: MASS_1 | |
| Faktor1 | Abhängige Variable |
| 1 | t0_SF36_derzeitig_im_Vergleich |
| 2 | t1_SF36_derzeitig_im_Vergleich |

| **Zwischensubjektfaktoren** | | |
| --- | --- | --- |
|  | | N |
| t1_Geschlecht | 0 | 14 |
|  | 1 | 23 |

| **Deskriptive Statistiken** | | | | |
| --- | --- | --- | --- | --- |
|  | t1_Geschlecht | Mittelwert | Standardabweichung | N |
| t0_SF36_derzeitig_im_Vergleich | 0 | 2,786 | ,5789 | 14 |
|  | 1 | 3,174 | ,7168 | 23 |
|  | Gesamt | 3,027 | ,6866 | 37 |
| t1_SF36_derzeitig_im_Vergleich | 0 | 3,000 | ,6794 | 14 |
|  | 1 | 3,174 | ,9841 | 23 |
|  | Gesamt | 3,108 | ,8751 | 37 |

| **Tests der Innersubjektkontraste** | | | | | | |
| --- | --- | --- | --- | --- | --- | --- |
| Maß: MASS_1 | | | | | | |
| Quelle | Faktor1 | Quadratsumme vom Typ III | df | Mittel der Quadrate | F | Sig. |
| Faktor1 | Linear | 4,286E-005 | 1 | 4,286E-005 | ,000 | ,992 |
| Faktor1 * t1_Tagepostop | Linear | ,084 | 1 | ,084 | ,177 | ,677 |
| Faktor1 * t1_Geschlecht | Linear | ,196 | 1 | ,196 | ,415 | ,524 |
| Fehler(Faktor1) | Linear | 16,095 | 34 | ,473 |  |  |

| **Tests der Zwischensubjekteffekte** | | | | | |
| --- | --- | --- | --- | --- | --- |
| Maß: MASS_1  Transformierte Variable: Mittel | | | | | |
| Quelle | Quadratsumme vom Typ III | df | Mittel der Quadrate | F | Sig. |
| Konstanter Term | 151,596 | 1 | 151,596 | 207,349 | ,000 |
| t1_Tagepostop | 1,929 | 1 | 1,929 | 2,639 | ,114 |
| t1_Geschlecht | 1,417 | 1 | 1,417 | 1,938 | ,173 |
| Fehler | 24,858 | 34 | ,731 |  |  |

- Bestätigt t-Test: Kein Vorher-Nachher-Unterschied
- Auch kein Geschlechtsunterschied und keine Wechselwirkung
- T-test alleine reicht..

**T-Test**

| **Statistik bei gepaarten Stichproben** | | | | | |
| --- | --- | --- | --- | --- | --- |
|  | | Mittelwert | N | Standardabweichung | Standardfehler des Mittelwertes |
| Paaren 1 | t0_Gehleistung | 3,405 | 37 | 1,2124 | ,1993 |
|  | t1_Gehleistung | 4,000 | 37 | 1,1055 | ,1817 |

| **Korrelationen bei gepaarten Stichproben** | | | | |
| --- | --- | --- | --- | --- |
|  | | N | Korrelation | Signifikanz |
| Paaren 1 | t0_Gehleistung & t1_Gehleistung | 37 | ,788 | ,000 |

| **Test bei gepaarten Stichproben** | | | | | | | | | |
| --- | --- | --- | --- | --- | --- | --- | --- | --- | --- |
|  | | Gepaarte Differenzen | | | | | T | df | Sig. (2-seitig) |
|  |  | Mittelwert | Standardabweichung | Standardfehler des Mittelwertes | 95% Konfidenzintervall der Differenz | |  |  |  |
|  |  |  |  |  | Untere | Obere |  |  |  |
| Paaren 1 | t0_Gehleistung - t1_Gehleistung | -,5946 | ,7623 | ,1253 | -,8488 | -,3404 | -4,745 | 36 | ,000 |

- Signikanter Unterschied zwischen Vorher und Nachher (letzter Zeitpunkt): Wert wird höher

**Allgemeines Lineares Modell**

| **Innersubjektfaktoren** | |
| --- | --- |
| Maß: MASS_1 | |
| Faktor1 | Abhängige Variable |
| 1 | t0_Gehleistung |
| 2 | t1_Gehleistung |

| **Zwischensubjektfaktoren** | | |
| --- | --- | --- |
|  | | N |
| t1_Geschlecht | 0 | 14 |
|  | 1 | 23 |

| **Deskriptive Statistiken** | | | | |
| --- | --- | --- | --- | --- |
|  | t1_Geschlecht | Mittelwert | Standardabweichung | N |
| t0_Gehleistung | 0 | 3,429 | 1,2225 | 14 |
|  | 1 | 3,391 | 1,2336 | 23 |
|  | Gesamt | 3,405 | 1,2124 | 37 |
| t1_Gehleistung | 0 | 4,143 | ,8644 | 14 |
|  | 1 | 3,913 | 1,2400 | 23 |
|  | Gesamt | 4,000 | 1,1055 | 37 |

| **Tests der Innersubjektkontraste** | | | | | | |
| --- | --- | --- | --- | --- | --- | --- |
| Maß: MASS_1 | | | | | | |
| Quelle | Faktor1 | Quadratsumme vom Typ III | df | Mittel der Quadrate | F | Sig. |
| Faktor1 | Linear | 6,648 | 1 | 6,648 | 22,594 | ,000 |
| Faktor1 * t1_Geschlecht | Linear | ,161 | 1 | ,161 | ,548 | ,464 |
| Fehler(Faktor1) | Linear | 10,298 | 35 | ,294 |  |  |

| **Tests der Zwischensubjekteffekte** | | | | | |
| --- | --- | --- | --- | --- | --- |
| Maß: MASS_1  Transformierte Variable: Mittel | | | | | |
| Quelle | Quadratsumme vom Typ III | df | Mittel der Quadrate | F | Sig. |
| Konstanter Term | 962,905 | 1 | 962,905 | 391,202 | ,000 |
| t1_Geschlecht | ,310 | 1 | ,310 | ,126 | ,725 |
| Fehler | 86,149 | 35 | 2,461 |  |  |

- Bestätigt t-Test: Nur Vorher-Nachher-Unterschied
- Kein Geschlechtsunterschied und keine Wechselwirkung
- T-test alleine reicht…und stabiler als ANOVA, weil diese Variable eigentlich ordinalskaliert ist…

**Allgemeines Lineares Modell: Mit TagepostOP als Kovariate**

| **Innersubjektfaktoren** | |
| --- | --- |
| Maß: MASS_1 | |
| Faktor1 | Abhängige Variable |
| 1 | t0_Gehleistung |
| 2 | t1_Gehleistung |

| **Zwischensubjektfaktoren** | | |
| --- | --- | --- |
|  | | N |
| t1_Geschlecht | 0 | 14 |
|  | 1 | 23 |

| **Deskriptive Statistiken** | | | | |
| --- | --- | --- | --- | --- |
|  | t1_Geschlecht | Mittelwert | Standardabweichung | N |
| t0_Gehleistung | 0 | 3,429 | 1,2225 | 14 |
|  | 1 | 3,391 | 1,2336 | 23 |
|  | Gesamt | 3,405 | 1,2124 | 37 |
| t1_Gehleistung | 0 | 4,143 | ,8644 | 14 |
|  | 1 | 3,913 | 1,2400 | 23 |
|  | Gesamt | 4,000 | 1,1055 | 37 |

| **Tests der Innersubjektkontraste** | | | | | | |
| --- | --- | --- | --- | --- | --- | --- |
| Maß: MASS_1 | | | | | | |
| Quelle | Faktor1 | Quadratsumme vom Typ III | df | Mittel der Quadrate | F | Sig. |
| Faktor1 | Linear | ,301 | 1 | ,301 | 1,096 | ,303 |
| Faktor1 * t1_Tagepostop | Linear | ,951 | 1 | ,951 | 3,458 | ,072 |
| Faktor1 * t1_Geschlecht | Linear | ,151 | 1 | ,151 | ,551 | ,463 |
| Fehler(Faktor1) | Linear | 9,347 | 34 | ,275 |  |  |

| **Levene-Test auf Gleichheit der Fehlervarianzen** | | | | |
| --- | --- | --- | --- | --- |
|  | F | df1 | df2 | Sig. |
| t0_Gehleistung | ,128 | 1 | 35 | ,722 |
| t1_Gehleistung | ,681 | 1 | 35 | ,415 |

| **Tests der Zwischensubjekteffekte** | | | | | |
| --- | --- | --- | --- | --- | --- |
| Maß: MASS_1  Transformierte Variable: Mittel | | | | | |
| Quelle | Quadratsumme vom Typ III | df | Mittel der Quadrate | F | Sig. |
| Konstanter Term | 174,142 | 1 | 174,142 | 83,899 | ,000 |
| t1_Tagepostop | 15,578 | 1 | 15,578 | 7,505 | ,010 |
| t1_Geschlecht | ,256 | 1 | ,256 | ,124 | ,727 |
| Fehler | 70,571 | 34 | 2,076 |  |  |

- Wenn TagepostOP „neutralisiert“, dann kein Zeitunterschied mehr…

**Ab jetzt Auswertung für Konfidenzintervalle, aber da die drei Variablen eigentlich ordinal skaliert sind, ist das eher nicht zu verwenden…**

**Deskriptive Statistik**

| **Deskriptive Statistik** | | | | | | |
| --- | --- | --- | --- | --- | --- | --- |
|  | N | Minimum | Maximum | Mittelwert | | Standardabweichung |
|  | Statistik | Statistik | Statistik | Statistik | Standardfehler | Statistik |
| t0_SF36_allgemein | 37 | 2,0 | 4,0 | 3,000 | ,1025 | ,6236 |
| t1_SF36_allgemein | 37 | 1,0 | 4,0 | 2,946 | ,1159 | ,7050 |
| t0_SF36_derzeitig_im_Vergleich | 37 | 2,0 | 4,0 | 3,027 | ,1129 | ,6866 |
| t1_SF36_derzeitig_im_Vergleich | 37 | 1,0 | 5,0 | 3,108 | ,1439 | ,8751 |
| t0_Gehleistung | 37 | 1,0 | 5,0 | 3,405 | ,1993 | 1,2124 |
| t1_Gehleistung | 37 | 2,0 | 5,0 | 4,000 | ,1817 | 1,1055 |
| Gültige Werte (Listenweise) | 37 |  |  |  |  |  |

**Deskriptive Statistik**

**t0_Geschlecht = 0**

| **Deskriptive Statistik** | | | | | | |
| --- | --- | --- | --- | --- | --- | --- |
|  | N | Minimum | Maximum | Mittelwert | | Standardabweichung |
|  | Statistik | Statistik | Statistik | Statistik | Standardfehler | Statistik |
| t0_SF36_allgemein | 14 | 2,0 | 4,0 | 2,786 | ,1869 | ,6993 |
| t1_SF36_allgemein | 14 | 2,0 | 4,0 | 2,929 | ,1646 | ,6157 |
| t0_SF36_derzeitig_im_Vergleich | 14 | 2,0 | 4,0 | 2,786 | ,1547 | ,5789 |
| t1_SF36_derzeitig_im_Vergleich | 14 | 2,0 | 5,0 | 3,000 | ,1816 | ,6794 |
| t0_Gehleistung | 14 | 1,0 | 5,0 | 3,429 | ,3267 | 1,2225 |
| t1_Gehleistung | 14 | 2,0 | 5,0 | 4,143 | ,2310 | ,8644 |
| Gültige Werte (Listenweise) | 14 |  |  |  |  |  |

**t0_Geschlecht = 1**

| **Deskriptive Statistik** | | | | | | |
| --- | --- | --- | --- | --- | --- | --- |
|  | N | Minimum | Maximum | Mittelwert | | Standardabweichung |
|  | Statistik | Statistik | Statistik | Statistik | Standardfehler | Statistik |
| t0_SF36_allgemein | 23 | 2,0 | 4,0 | 3,130 | ,1143 | ,5481 |
| t1_SF36_allgemein | 23 | 1,0 | 4,0 | 2,957 | ,1600 | ,7674 |
| t0_SF36_derzeitig_im_Vergleich | 23 | 2,0 | 4,0 | 3,174 | ,1495 | ,7168 |
| t1_SF36_derzeitig_im_Vergleich | 23 | 1,0 | 5,0 | 3,174 | ,2052 | ,9841 |
| t0_Gehleistung | 23 | 1,0 | 5,0 | 3,391 | ,2572 | 1,2336 |
| t1_Gehleistung | 23 | 2,0 | 5,0 | 3,913 | ,2586 | 1,2400 |
| Gültige Werte (Listenweise) | 23 |  |  |  |  |  |
