# Supplementary material for: Intra-articular injection of bone marrow aspirate concentrate (mesenchymal stem cells) in KL grade III and IV knee osteoarthritis: 4 year results of 37 knees
Source: Sci Rep. 2024 Feb 1;14:2665. doi: 10.1038/s41598-024-51410-2 (PMC10834500; doi:10.1038/s41598-024-51410-2)
Supplement: Supplementary file 6 — Supplementary Information 6. [file 41598_2024_51410_MOESM6_ESM.docx]

**Nichtparametrische Korrelationen**

| **Korrelationen** | | | | | | | | | | |
| --- | --- | --- | --- | --- | --- | --- | --- | --- | --- | --- |
|  | | | SF36_allgemein | SF36_derzeitig_im_Vergleich | Gehleistung | Größe in cm | Gewicht in kg | BMI | ZEITPUNKT | IKDC |
| Spearman-Rho | SF36_allgemein | Korrelationskoeffizient | 1,000 | ,221 | ,059 | ,121 | ,067 | ,026 | -,044 | ,129 |
|  |  | Sig. (2-seitig) | . | ,040 | ,588 | ,268 | ,543 | ,810 | ,686 | ,237 |
|  |  | N | 86 | 86 | 86 | 86 | 86 | 86 | 86 | 86 |
|  | SF36_derzeitig_im_Vergleich | Korrelationskoeffizient | ,221 | 1,000 | ,312 | -,023 | -,048 | -,075 | ,166 | ,391 |
|  |  | Sig. (2-seitig) | ,040 | . | ,003 | ,832 | ,659 | ,495 | ,126 | ,000 |
|  |  | N | 86 | 86 | 86 | 86 | 86 | 86 | 86 | 86 |
|  | Gehleistung | Korrelationskoeffizient | ,059 | ,312 | 1,000 | -,007 | -,214 | -,318 | ,404 | ,668 |
|  |  | Sig. (2-seitig) | ,588 | ,003 | . | ,951 | ,048 | ,003 | ,000 | ,000 |
|  |  | N | 86 | 86 | 86 | 86 | 86 | 86 | 86 | 86 |
|  | Größe in cm | Korrelationskoeffizient | ,121 | -,023 | -,007 | 1,000 | ,832 | ,591 | -,012 | -,054 |
|  |  | Sig. (2-seitig) | ,268 | ,832 | ,951 | . | ,000 | ,000 | ,910 | ,621 |
|  |  | N | 86 | 86 | 86 | 86 | 86 | 86 | 86 | 86 |
|  | Gewicht in kg | Korrelationskoeffizient | ,067 | -,048 | -,214 | ,832 | 1,000 | ,923 | -,029 | -,098 |
|  |  | Sig. (2-seitig) | ,543 | ,659 | ,048 | ,000 | . | ,000 | ,789 | ,370 |
|  |  | N | 86 | 86 | 86 | 86 | 86 | 86 | 86 | 86 |
|  | BMI | Korrelationskoeffizient | ,026 | -,075 | -,318 | ,591 | ,923 | 1,000 | -,001 | -,146 |
|  |  | Sig. (2-seitig) | ,810 | ,495 | ,003 | ,000 | ,000 | . | ,992 | ,180 |
|  |  | N | 86 | 86 | 86 | 86 | 86 | 86 | 86 | 86 |
|  | ZEITPUNKT | Korrelationskoeffizient | -,044 | ,166 | ,404 | -,012 | -,029 | -,001 | 1,000 | ,562 |
|  |  | Sig. (2-seitig) | ,686 | ,126 | ,000 | ,910 | ,789 | ,992 | . | ,000 |
|  |  | N | 86 | 86 | 86 | 86 | 86 | 86 | 86 | 86 |
|  | IKDC | Korrelationskoeffizient | ,129 | ,391 | ,668 | -,054 | -,098 | -,146 | ,562 | 1,000 |
|  |  | Sig. (2-seitig) | ,237 | ,000 | ,000 | ,621 | ,370 | ,180 | ,000 | . |
|  |  | N | 86 | 86 | 86 | 86 | 86 | 86 | 86 | 86 |

- Beide SF36-Varaiblen sind unabhängig von diversen anderen Variablen.
- Gehleistung ist korreliert mit Gewicht, BMI, Zeitpunkt und IKDC

**Nichtparametrische Korrelationen**

**Vorher_Nachher = 0**

| **Korrelationen** | | | | | | | | | | |
| --- | --- | --- | --- | --- | --- | --- | --- | --- | --- | --- |
|  | | | SF36_allgemein | SF36_derzeitig_im_Vergleich | Gehleistung | Größe in cm | Gewicht in kg | BMI | ZEITPUNKT | IKDC |
| Spearman-Rho | SF36_allgemein | Korrelationskoeffizient | 1,000 | ,189 | ,051 | ,258 | ,165 | ,086 | . | ,194 |
|  |  | Sig. (2-seitig) | . | ,263 | ,764 | ,123 | ,330 | ,611 | . | ,251 |
|  |  | N | 37 | 37 | 37 | 37 | 37 | 37 | 37 | 37 |
|  | SF36_derzeitig_im_Vergleich | Korrelationskoeffizient | ,189 | 1,000 | ,380 | ,159 | ,042 | -,067 | . | ,541 |
|  |  | Sig. (2-seitig) | ,263 | . | ,020 | ,347 | ,805 | ,694 | . | ,001 |
|  |  | N | 37 | 37 | 37 | 37 | 37 | 37 | 37 | 37 |
|  | Gehleistung | Korrelationskoeffizient | ,051 | ,380 | 1,000 | ,021 | -,223 | -,373 | . | ,679 |
|  |  | Sig. (2-seitig) | ,764 | ,020 | . | ,902 | ,184 | ,023 | . | ,000 |
|  |  | N | 37 | 37 | 37 | 37 | 37 | 37 | 37 | 37 |
|  | Größe in cm | Korrelationskoeffizient | ,258 | ,159 | ,021 | 1,000 | ,820 | ,566 | . | ,191 |
|  |  | Sig. (2-seitig) | ,123 | ,347 | ,902 | . | ,000 | ,000 | . | ,257 |
|  |  | N | 37 | 37 | 37 | 37 | 37 | 37 | 37 | 37 |
|  | Gewicht in kg | Korrelationskoeffizient | ,165 | ,042 | -,223 | ,820 | 1,000 | ,921 | . | ,108 |
|  |  | Sig. (2-seitig) | ,330 | ,805 | ,184 | ,000 | . | ,000 | . | ,523 |
|  |  | N | 37 | 37 | 37 | 37 | 37 | 37 | 37 | 37 |
|  | BMI | Korrelationskoeffizient | ,086 | -,067 | -,373 | ,566 | ,921 | 1,000 | . | -,026 |
|  |  | Sig. (2-seitig) | ,611 | ,694 | ,023 | ,000 | ,000 | . | . | ,877 |
|  |  | N | 37 | 37 | 37 | 37 | 37 | 37 | 37 | 37 |
|  | ZEITPUNKT | Korrelationskoeffizient | . | . | . | . | . | . | . | . |
|  |  | Sig. (2-seitig) | . | . | . | . | . | . | . | . |
|  |  | N | 37 | 37 | 37 | 37 | 37 | 37 | 37 | 37 |
|  | IKDC | Korrelationskoeffizient | ,194 | ,541 | ,679 | ,191 | ,108 | -,026 | . | 1,000 |
|  |  | Sig. (2-seitig) | ,251 | ,001 | ,000 | ,257 | ,523 | ,877 | . | . |
|  |  | N | 37 | 37 | 37 | 37 | 37 | 37 | 37 | 37 |

- Wie „overall“ auch für nur vorher, plus hier SF36-Vergleich signifikant mit IKDC korreliert

**Vorher_Nachher = 1**

| **Korrelationen** | | | | | | | | | | |
| --- | --- | --- | --- | --- | --- | --- | --- | --- | --- | --- |
|  | | | SF36_allgemein | SF36_derzeitig_im_Vergleich | Gehleistung | Größe in cm | Gewicht in kg | BMI | ZEITPUNKT | IKDC |
| Spearman-Rho | SF36_allgemein | Korrelationskoeffizient | 1,000 | ,252 | ,051 | ,012 | -,017 | -,033 | -,080 | ,152 |
|  |  | Sig. (2-seitig) | . | ,081 | ,728 | ,936 | ,907 | ,824 | ,585 | ,296 |
|  |  | N | 49 | 49 | 49 | 49 | 49 | 49 | 49 | 49 |
|  | SF36_derzeitig_im_Vergleich | Korrelationskoeffizient | ,252 | 1,000 | ,219 | -,157 | -,111 | -,084 | ,208 | ,277 |
|  |  | Sig. (2-seitig) | ,081 | . | ,131 | ,281 | ,449 | ,566 | ,151 | ,054 |
|  |  | N | 49 | 49 | 49 | 49 | 49 | 49 | 49 | 49 |
|  | Gehleistung | Korrelationskoeffizient | ,051 | ,219 | 1,000 | ,003 | -,174 | -,265 | ,354 | ,571 |
|  |  | Sig. (2-seitig) | ,728 | ,131 | . | ,986 | ,232 | ,066 | ,012 | ,000 |
|  |  | N | 49 | 49 | 49 | 49 | 49 | 49 | 49 | 49 |
|  | Größe in cm | Korrelationskoeffizient | ,012 | -,157 | ,003 | 1,000 | ,842 | ,609 | -,029 | -,152 |
|  |  | Sig. (2-seitig) | ,936 | ,281 | ,986 | . | ,000 | ,000 | ,844 | ,297 |
|  |  | N | 49 | 49 | 49 | 49 | 49 | 49 | 49 | 49 |
|  | Gewicht in kg | Korrelationskoeffizient | -,017 | -,111 | -,174 | ,842 | 1,000 | ,923 | -,036 | -,175 |
|  |  | Sig. (2-seitig) | ,907 | ,449 | ,232 | ,000 | . | ,000 | ,806 | ,228 |
|  |  | N | 49 | 49 | 49 | 49 | 49 | 49 | 49 | 49 |
|  | BMI | Korrelationskoeffizient | -,033 | -,084 | -,265 | ,609 | ,923 | 1,000 | ,022 | -,216 |
|  |  | Sig. (2-seitig) | ,824 | ,566 | ,066 | ,000 | ,000 | . | ,882 | ,136 |
|  |  | N | 49 | 49 | 49 | 49 | 49 | 49 | 49 | 49 |
|  | ZEITPUNKT | Korrelationskoeffizient | -,080 | ,208 | ,354 | -,029 | -,036 | ,022 | 1,000 | ,162 |
|  |  | Sig. (2-seitig) | ,585 | ,151 | ,012 | ,844 | ,806 | ,882 | . | ,266 |
|  |  | N | 49 | 49 | 49 | 49 | 49 | 49 | 49 | 49 |
|  | IKDC | Korrelationskoeffizient | ,152 | ,277 | ,571 | -,152 | -,175 | -,216 | ,162 | 1,000 |
|  |  | Sig. (2-seitig) | ,296 | ,054 | ,000 | ,297 | ,228 | ,136 | ,266 | . |
|  |  | N | 49 | 49 | 49 | 49 | 49 | 49 | 49 | 49 |

- Nachher nur mehr Gehleistung und Zeitpunkt bzw. Gehleistung und IKDC korreliert, andere nur mehr tendenziell oder nicht mehr…

**Nichtparametrische Tests**

**Vorher_Nachher = 0**

| **Kolmogorov-Smirnov-Anpassungstest** | | | | |
| --- | --- | --- | --- | --- |
|  | | SF36_allgemein | SF36_derzeitig_im_Vergleich | Gehleistung |
| N | | 37 | 37 | 37 |
| Parameter der Normalverteilung | Mittelwert | 3,000 | 3,027 | 3,405 |
|  | Standardabweichung | ,6236 | ,6866 | 1,2124 |
| Extremste Differenzen | Absolut | ,311 | ,272 | ,229 |
|  | Positiv | ,311 | ,272 | ,123 |
|  | Negativ | -,311 | -,268 | -,229 |
| Kolmogorov-Smirnov-Z | | 1,891 | 1,657 | 1,391 |
| Asymptotische Signifikanz (2-seitig) | | ,002 | ,008 | ,042 |

- Alle drei, aber vor allem die beiden SF36 etwas problematisch wegen Normalverteilung.
- Klar, sie sind ja eigentlich ordinalskaliert und daher sowieso kritisch für t-Test und ANOVA

**Vorher_Nachher = 1**

| **Kolmogorov-Smirnov-Anpassungstest** | | | | |
| --- | --- | --- | --- | --- |
|  | | SF36_allgemein | SF36_derzeitig_im_Vergleich | Gehleistung |
| N | | 49 | 49 | 49 |
| Parameter der Normalverteilung | Mittelwert | 2,959 | 3,245 | 4,122 |
|  | Standardabweichung | ,6110 | ,9021 | 1,0131 |
| Extremste Differenzen | Absolut | ,363 | ,321 | ,289 |
|  | Positiv | ,331 | ,321 | ,193 |
|  | Negativ | -,363 | -,250 | -,289 |
| Kolmogorov-Smirnov-Z | | 2,544 | 2,249 | 2,020 |
| Asymptotische Signifikanz (2-seitig) | | ,000 | ,000 | ,001 |

- Detto…

**T-Test**

| **Gruppenstatistiken** | | | | | |
| --- | --- | --- | --- | --- | --- |
|  | Vorher_Nachher | N | Mittelwert | Standardabweichung | Standardfehler des Mittelwertes |
| SF36_allgemein | 0 | 37 | 3,000 | ,6236 | ,1025 |
|  | 1 | 49 | 2,959 | ,6110 | ,0873 |
| SF36_derzeitig_im_Vergleich | 0 | 37 | 3,027 | ,6866 | ,1129 |
|  | 1 | 49 | 3,245 | ,9021 | ,1289 |
| Gehleistung | 0 | 37 | 3,405 | 1,2124 | ,1993 |
|  | 1 | 49 | 4,122 | 1,0131 | ,1447 |

| **Test bei unabhängigen Stichproben** | | | | | | | | | | |
| --- | --- | --- | --- | --- | --- | --- | --- | --- | --- | --- |
|  | | Levene-Test der Varianzgleichheit | | T-Test für die Mittelwertgleichheit | | | | | | |
|  |  | F | Signifikanz | T | df | Sig. (2-seitig) | Mittlere Differenz | Standardfehler der Differenz | 95% Konfidenzintervall der Differenz | |
|  |  |  |  |  |  |  |  |  | Untere | Obere |
| SF36_allgemein | Varianzen sind gleich | ,051 | ,821 | ,304 | 84 | ,762 | ,0408 | ,1343 | -,2262 | ,3078 |
|  |  |  |  |  |  |  |  |  |  |  |
| SF36_derzeitig_im_Vergleich | Varianzen sind gleich | 2,883 | ,093 | -1,225 | 84 | ,224 | -,2179 | ,1779 | -,5716 | ,1359 |
|  |  |  |  |  |  |  |  |  |  |  |
| Gehleistung | Varianzen sind gleich | 3,572 | ,062 | -2,985 | 84 | ,004 | -,7170 | ,2402 | -1,1947 | -,2393 |
|  |  |  |  |  |  |  |  |  |  |  |

- Keine Unterschied bei SF36, signifikanter Unterschied bei Gehleistung

**Ab jetzt: Erstens kritisch wegen ordinalen Daten, zweitens bestätigend für t-Test und nicht wirklich relevantes dazu. DH eher weglassen.**

**Univariate Varianzanalyse**

| **Zwischensubjektfaktoren** | | |
| --- | --- | --- |
|  | | N |
| Vorher_Nachher | 0 | 37 |
|  | 1 | 49 |
| Geschlecht | 0 | 32 |
|  | 1 | 54 |

| **Deskriptive Statistiken** | | | | |
| --- | --- | --- | --- | --- |
| Abhängige Variable: SF36_allgemein | | | | |
| Vorher_Nachher | Geschlecht | Mittelwert | Standardabweichung | N |
| 0 | 0 | 2,786 | ,6993 | 14 |
|  | 1 | 3,130 | ,5481 | 23 |
|  | Gesamt | 3,000 | ,6236 | 37 |
| 1 | 0 | 2,944 | ,5393 | 18 |
|  | 1 | 2,968 | ,6575 | 31 |
|  | Gesamt | 2,959 | ,6110 | 49 |
| Gesamt | 0 | 2,875 | ,6091 | 32 |
|  | 1 | 3,037 | ,6132 | 54 |
|  | Gesamt | 2,977 | ,6131 | 86 |

| **Tests der Zwischensubjekteffekte** | | | | | |
| --- | --- | --- | --- | --- | --- |
| Abhängige Variable: SF36_allgemein | | | | | |
| Quelle | Quadratsumme vom Typ III | df | Mittel der Quadrate | F | Sig. |
| Korrigiertes Modell | 1,075 | 3 | ,358 | ,952 | ,420 |
| Konstanter Term | 690,160 | 1 | 690,160 | 1832,796 | ,000 |
| Vorher_Nachher | 7,746E-005 | 1 | 7,746E-005 | ,000 | ,989 |
| Geschlecht | ,668 | 1 | ,668 | 1,774 | ,187 |
| Vorher_Nachher * Geschlecht | ,510 | 1 | ,510 | 1,353 | ,248 |
| Fehler | 30,878 | 82 | ,377 |  |  |
| Gesamt | 794,000 | 86 |  |  |  |
| Korrigierte Gesamtvariation | 31,953 | 85 |  |  |  |

**Univariate Varianzanalyse**

| **Zwischensubjektfaktoren** | | |
| --- | --- | --- |
|  | | N |
| Vorher_Nachher | 0 | 37 |
|  | 1 | 49 |
| Geschlecht | 0 | 32 |
|  | 1 | 54 |

| **Deskriptive Statistiken** | | | | |
| --- | --- | --- | --- | --- |
| Abhängige Variable: SF36_allgemein | | | | |
| Vorher_Nachher | Geschlecht | Mittelwert | Standardabweichung | N |
| 0 | 0 | 2,786 | ,6993 | 14 |
|  | 1 | 3,130 | ,5481 | 23 |
|  | Gesamt | 3,000 | ,6236 | 37 |
| 1 | 0 | 2,944 | ,5393 | 18 |
|  | 1 | 2,968 | ,6575 | 31 |
|  | Gesamt | 2,959 | ,6110 | 49 |
| Gesamt | 0 | 2,875 | ,6091 | 32 |
|  | 1 | 3,037 | ,6132 | 54 |
|  | Gesamt | 2,977 | ,6131 | 86 |

| **Tests der Zwischensubjekteffekte** | | | | | |
| --- | --- | --- | --- | --- | --- |
| Abhängige Variable: SF36_allgemein | | | | | |
| Quelle | Quadratsumme vom Typ III | df | Mittel der Quadrate | F | Sig. |
| Korrigiertes Modell | 1,607 | 5 | ,321 | ,847 | ,520 |
| Konstanter Term | 19,840 | 1 | 19,840 | 52,302 | ,000 |
| BMI | ,278 | 1 | ,278 | ,733 | ,395 |
| ZEITPUNKT | ,251 | 1 | ,251 | ,662 | ,418 |
| Vorher_Nachher | ,157 | 1 | ,157 | ,413 | ,522 |
| Geschlecht | ,914 | 1 | ,914 | 2,410 | ,124 |
| Vorher_Nachher * Geschlecht | ,543 | 1 | ,543 | 1,430 | ,235 |
| Fehler | 30,347 | 80 | ,379 |  |  |
| Gesamt | 794,000 | 86 |  |  |  |
| Korrigierte Gesamtvariation | 31,953 | 85 |  |  |  |

**Univariate Varianzanalyse**

| **Zwischensubjektfaktoren** | | |
| --- | --- | --- |
|  | | N |
| Vorher_Nachher | 0 | 37 |
|  | 1 | 49 |
| Geschlecht | 0 | 32 |
|  | 1 | 54 |

| **Deskriptive Statistiken** | | | | |
| --- | --- | --- | --- | --- |
| Abhängige Variable: SF36_derzeitig_im_Vergleich | | | | |
| Vorher_Nachher | Geschlecht | Mittelwert | Standardabweichung | N |
| 0 | 0 | 2,786 | ,5789 | 14 |
|  | 1 | 3,174 | ,7168 | 23 |
|  | Gesamt | 3,027 | ,6866 | 37 |
| 1 | 0 | 3,278 | ,8948 | 18 |
|  | 1 | 3,226 | ,9205 | 31 |
|  | Gesamt | 3,245 | ,9021 | 49 |
| Gesamt | 0 | 3,063 | ,8007 | 32 |
|  | 1 | 3,204 | ,8328 | 54 |
|  | Gesamt | 3,151 | ,8191 | 86 |

| **Tests der Zwischensubjekteffekte** | | | | | |
| --- | --- | --- | --- | --- | --- |
| Abhängige Variable: SF36_derzeitig_im_Vergleich | | | | | |
| Quelle | Quadratsumme vom Typ III | df | Mittel der Quadrate | F | Sig. |
| Korrigiertes Modell | 2,343 | 3 | ,781 | 1,171 | ,326 |
| Konstanter Term | 766,236 | 1 | 766,236 | 1148,822 | ,000 |
| Vorher_Nachher | 1,460 | 1 | 1,460 | 2,188 | ,143 |
| Geschlecht | ,558 | 1 | ,558 | ,836 | ,363 |
| Vorher_Nachher * Geschlecht | ,956 | 1 | ,956 | 1,433 | ,235 |
| Fehler | 54,692 | 82 | ,667 |  |  |
| Gesamt | 911,000 | 86 |  |  |  |
| Korrigierte Gesamtvariation | 57,035 | 85 |  |  |  |

**Univariate Varianzanalyse**

| **Zwischensubjektfaktoren** | | |
| --- | --- | --- |
|  | | N |
| Vorher_Nachher | 0 | 37 |
|  | 1 | 49 |
| Geschlecht | 0 | 32 |
|  | 1 | 54 |

| **Deskriptive Statistiken** | | | | |
| --- | --- | --- | --- | --- |
| Abhängige Variable: SF36_derzeitig_im_Vergleich | | | | |
| Vorher_Nachher | Geschlecht | Mittelwert | Standardabweichung | N |
| 0 | 0 | 2,786 | ,5789 | 14 |
|  | 1 | 3,174 | ,7168 | 23 |
|  | Gesamt | 3,027 | ,6866 | 37 |
| 1 | 0 | 3,278 | ,8948 | 18 |
|  | 1 | 3,226 | ,9205 | 31 |
|  | Gesamt | 3,245 | ,9021 | 49 |
| Gesamt | 0 | 3,063 | ,8007 | 32 |
|  | 1 | 3,204 | ,8328 | 54 |
|  | Gesamt | 3,151 | ,8191 | 86 |

| **Tests der Zwischensubjekteffekte** | | | | | |
| --- | --- | --- | --- | --- | --- |
| Abhängige Variable: SF36_derzeitig_im_Vergleich | | | | | |
| Quelle | Quadratsumme vom Typ III | df | Mittel der Quadrate | F | Sig. |
| Korrigiertes Modell | 4,001 | 5 | ,800 | 1,207 | ,314 |
| Konstanter Term | 18,947 | 1 | 18,947 | 28,580 | ,000 |
| BMI | ,310 | 1 | ,310 | ,467 | ,496 |
| ZEITPUNKT | 1,353 | 1 | 1,353 | 2,041 | ,157 |
| Vorher_Nachher | ,057 | 1 | ,057 | ,086 | ,770 |
| Geschlecht | ,932 | 1 | ,932 | 1,407 | ,239 |
| Vorher_Nachher * Geschlecht | ,879 | 1 | ,879 | 1,326 | ,253 |
| Fehler | 53,034 | 80 | ,663 |  |  |
| Gesamt | 911,000 | 86 |  |  |  |
| Korrigierte Gesamtvariation | 57,035 | 85 |  |  |  |

**Univariate Varianzanalyse**

| **Zwischensubjektfaktoren** | | |
| --- | --- | --- |
|  | | N |
| Vorher_Nachher | 0 | 37 |
|  | 1 | 49 |
| Geschlecht | 0 | 32 |
|  | 1 | 54 |

| **Deskriptive Statistiken** | | | | |
| --- | --- | --- | --- | --- |
| Abhängige Variable: Gehleistung | | | | |
| Vorher_Nachher | Geschlecht | Mittelwert | Standardabweichung | N |
| 0 | 0 | 3,429 | 1,2225 | 14 |
|  | 1 | 3,391 | 1,2336 | 23 |
|  | Gesamt | 3,405 | 1,2124 | 37 |
| 1 | 0 | 4,222 | ,8085 | 18 |
|  | 1 | 4,065 | 1,1236 | 31 |
|  | Gesamt | 4,122 | 1,0131 | 49 |
| Gesamt | 0 | 3,875 | 1,0701 | 32 |
|  | 1 | 3,778 | 1,2079 | 54 |
|  | Gesamt | 3,814 | 1,1531 | 86 |

| **Tests der Zwischensubjekteffekte** | | | | | |
| --- | --- | --- | --- | --- | --- |
| Abhängige Variable: Gehleistung | | | | | |
| Quelle | Quadratsumme vom Typ III | df | Mittel der Quadrate | F | Sig. |
| Korrigiertes Modell | 11,134 | 3 | 3,711 | 2,987 | ,036 |
| Konstanter Term | 1125,736 | 1 | 1125,736 | 905,990 | ,000 |
| Vorher_Nachher | 10,614 | 1 | 10,614 | 8,542 | ,004 |
| Geschlecht | ,188 | 1 | ,188 | ,151 | ,699 |
| Vorher_Nachher * Geschlecht | ,072 | 1 | ,072 | ,058 | ,811 |
| Fehler | 101,889 | 82 | 1,243 |  |  |
| Gesamt | 1364,000 | 86 |  |  |  |
| Korrigierte Gesamtvariation | 113,023 | 85 |  |  |  |

**Univariate Varianzanalyse**

| **Zwischensubjektfaktoren** | | |
| --- | --- | --- |
|  | | N |
| Vorher_Nachher | 0 | 37 |
|  | 1 | 49 |
| Geschlecht | 0 | 32 |
|  | 1 | 54 |

| **Deskriptive Statistiken** | | | | |
| --- | --- | --- | --- | --- |
| Abhängige Variable: Gehleistung | | | | |
| Vorher_Nachher | Geschlecht | Mittelwert | Standardabweichung | N |
| 0 | 0 | 3,429 | 1,2225 | 14 |
|  | 1 | 3,391 | 1,2336 | 23 |
|  | Gesamt | 3,405 | 1,2124 | 37 |
| 1 | 0 | 4,222 | ,8085 | 18 |
|  | 1 | 4,065 | 1,1236 | 31 |
|  | Gesamt | 4,122 | 1,0131 | 49 |
| Gesamt | 0 | 3,875 | 1,0701 | 32 |
|  | 1 | 3,778 | 1,2079 | 54 |
|  | Gesamt | 3,814 | 1,1531 | 86 |

| **Tests der Zwischensubjekteffekte** | | | | | |
| --- | --- | --- | --- | --- | --- |
| Abhängige Variable: Gehleistung | | | | | |
| Quelle | Quadratsumme vom Typ III | df | Mittel der Quadrate | F | Sig. |
| Korrigiertes Modell | 38,419 | 5 | 7,684 | 8,240 | ,000 |
| Konstanter Term | 74,960 | 1 | 74,960 | 80,381 | ,000 |
| BMI | 19,763 | 1 | 19,763 | 21,192 | ,000 |
| ZEITPUNKT | 7,622 | 1 | 7,622 | 8,174 | ,005 |
| Vorher_Nachher | ,134 | 1 | ,134 | ,143 | ,706 |
| Geschlecht | 4,517 | 1 | 4,517 | 4,844 | ,031 |
| Vorher_Nachher * Geschlecht | ,040 | 1 | ,040 | ,043 | ,837 |
| Fehler | 74,604 | 80 | ,933 |  |  |
| Gesamt | 1364,000 | 86 |  |  |  |
| Korrigierte Gesamtvariation | 113,023 | 85 |  |  |  |

Ab hier für Konfidenzintervalle, aber wegen ordinalskalierten Daten eh nicht wirklich relevant.

**Deskriptive Statistik**

| **Deskriptive Statistik** | | | | | | |
| --- | --- | --- | --- | --- | --- | --- |
|  | N | Minimum | Maximum | Mittelwert | | Standardabweichung |
|  | Statistik | Statistik | Statistik | Statistik | Standardfehler | Statistik |
| SF36_allgemein | 86 | 1,0 | 4,0 | 2,977 | ,0661 | ,6131 |
| SF36_derzeitig_im_Vergleich | 86 | 1,0 | 5,0 | 3,151 | ,0883 | ,8191 |
| Gehleistung | 86 | 1,0 | 5,0 | 3,814 | ,1243 | 1,1531 |
| Gültige Werte (Listenweise) | 86 |  |  |  |  |  |

**Deskriptive Statistik**

**Geschlecht = 0**

| **Deskriptive Statistik** | | | | | | |
| --- | --- | --- | --- | --- | --- | --- |
|  | N | Minimum | Maximum | Mittelwert | | Standardabweichung |
|  | Statistik | Statistik | Statistik | Statistik | Standardfehler | Statistik |
| SF36_allgemein | 32 | 2,0 | 4,0 | 2,875 | ,1077 | ,6091 |
| SF36_derzeitig_im_Vergleich | 32 | 2,0 | 5,0 | 3,063 | ,1415 | ,8007 |
| Gehleistung | 32 | 1,0 | 5,0 | 3,875 | ,1892 | 1,0701 |
| Gültige Werte (Listenweise) | 32 |  |  |  |  |  |

**Geschlecht = 1**

| **Deskriptive Statistik** | | | | | | |
| --- | --- | --- | --- | --- | --- | --- |
|  | N | Minimum | Maximum | Mittelwert | | Standardabweichung |
|  | Statistik | Statistik | Statistik | Statistik | Standardfehler | Statistik |
| SF36_allgemein | 54 | 1,0 | 4,0 | 3,037 | ,0834 | ,6132 |
| SF36_derzeitig_im_Vergleich | 54 | 1,0 | 5,0 | 3,204 | ,1133 | ,8328 |
| Gehleistung | 54 | 1,0 | 5,0 | 3,778 | ,1644 | 1,2079 |
| Gültige Werte (Listenweise) | 54 |  |  |  |  |  |

**Deskriptive Statistik**

**Vorher_Nachher = 0**

| **Deskriptive Statistik** | | | | | | |
| --- | --- | --- | --- | --- | --- | --- |
|  | N | Minimum | Maximum | Mittelwert | | Standardabweichung |
|  | Statistik | Statistik | Statistik | Statistik | Standardfehler | Statistik |
| SF36_allgemein | 37 | 2,0 | 4,0 | 3,000 | ,1025 | ,6236 |
| SF36_derzeitig_im_Vergleich | 37 | 2,0 | 4,0 | 3,027 | ,1129 | ,6866 |
| Gehleistung | 37 | 1,0 | 5,0 | 3,405 | ,1993 | 1,2124 |
| Gültige Werte (Listenweise) | 37 |  |  |  |  |  |

**Vorher_Nachher = 1**

| **Deskriptive Statistik** | | | | | | |
| --- | --- | --- | --- | --- | --- | --- |
|  | N | Minimum | Maximum | Mittelwert | | Standardabweichung |
|  | Statistik | Statistik | Statistik | Statistik | Standardfehler | Statistik |
| SF36_allgemein | 49 | 1,0 | 4,0 | 2,959 | ,0873 | ,6110 |
| SF36_derzeitig_im_Vergleich | 49 | 1,0 | 5,0 | 3,245 | ,1289 | ,9021 |
| Gehleistung | 49 | 2,0 | 5,0 | 4,122 | ,1447 | 1,0131 |
| Gültige Werte (Listenweise) | 49 |  |  |  |  |  |

**Deskriptive Statistik**

**ZEITPUNKT = 0**

| **Deskriptive Statistik** | | | | | | |
| --- | --- | --- | --- | --- | --- | --- |
|  | N | Minimum | Maximum | Mittelwert | | Standardabweichung |
|  | Statistik | Statistik | Statistik | Statistik | Standardfehler | Statistik |
| SF36_allgemein | 37 | 2,0 | 4,0 | 3,000 | ,1025 | ,6236 |
| SF36_derzeitig_im_Vergleich | 37 | 2,0 | 4,0 | 3,027 | ,1129 | ,6866 |
| Gehleistung | 37 | 1,0 | 5,0 | 3,405 | ,1993 | 1,2124 |
| Gültige Werte (Listenweise) | 37 |  |  |  |  |  |

**ZEITPUNKT = 1**

| **Deskriptive Statistik** | | | | | | |
| --- | --- | --- | --- | --- | --- | --- |
|  | N | Minimum | Maximum | Mittelwert | | Standardabweichung |
|  | Statistik | Statistik | Statistik | Statistik | Standardfehler | Statistik |
| SF36_allgemein | 13 | 2,0 | 4,0 | 2,846 | ,1910 | ,6887 |
| SF36_derzeitig_im_Vergleich | 13 | 2,0 | 4,0 | 2,769 | ,1662 | ,5991 |
| Gehleistung | 13 | 2,0 | 5,0 | 3,462 | ,3511 | 1,2659 |
| Gültige Werte (Listenweise) | 13 |  |  |  |  |  |

**ZEITPUNKT = 2**

| **Deskriptive Statistik** | | | | | | |
| --- | --- | --- | --- | --- | --- | --- |
|  | N | Minimum | Maximum | Mittelwert | | Standardabweichung |
|  | Statistik | Statistik | Statistik | Statistik | Standardfehler | Statistik |
| SF36_allgemein | 13 | 3,0 | 4,0 | 3,308 | ,1332 | ,4804 |
| SF36_derzeitig_im_Vergleich | 13 | 3,0 | 5,0 | 3,538 | ,2153 | ,7763 |
| Gehleistung | 13 | 2,0 | 5,0 | 4,231 | ,2571 | ,9268 |
| Gültige Werte (Listenweise) | 13 |  |  |  |  |  |

**ZEITPUNKT = 3**

| **Deskriptive Statistik** | | | | | | |
| --- | --- | --- | --- | --- | --- | --- |
|  | N | Minimum | Maximum | Mittelwert | | Standardabweichung |
|  | Statistik | Statistik | Statistik | Statistik | Standardfehler | Statistik |
| SF36_allgemein | 10 | 2,0 | 3,0 | 2,900 | ,1000 | ,3162 |
| SF36_derzeitig_im_Vergleich | 10 | 2,0 | 5,0 | 3,400 | ,3055 | ,9661 |
| Gehleistung | 10 | 2,0 | 5,0 | 4,200 | ,2906 | ,9189 |
| Gültige Werte (Listenweise) | 10 |  |  |  |  |  |

**ZEITPUNKT = 4**

| **Deskriptive Statistik** | | | | | | |
| --- | --- | --- | --- | --- | --- | --- |
|  | N | Minimum | Maximum | Mittelwert | | Standardabweichung |
|  | Statistik | Statistik | Statistik | Statistik | Standardfehler | Statistik |
| SF36_allgemein | 13 | 1,0 | 4,0 | 2,769 | ,2011 | ,7250 |
| SF36_derzeitig_im_Vergleich | 13 | 1,0 | 5,0 | 3,308 | ,3077 | 1,1094 |
| Gehleistung | 13 | 4,0 | 5,0 | 4,615 | ,1404 | ,5064 |
| Gültige Werte (Listenweise) | 13 |  |  |  |  |  |

**Deskriptive Statistik**

**Geschlecht = 0, Vorher_Nachher = 0**

| **Deskriptive Statistik** | | | | | | |
| --- | --- | --- | --- | --- | --- | --- |
|  | N | Minimum | Maximum | Mittelwert | | Standardabweichung |
|  | Statistik | Statistik | Statistik | Statistik | Standardfehler | Statistik |
| SF36_allgemein | 14 | 2,0 | 4,0 | 2,786 | ,1869 | ,6993 |
| SF36_derzeitig_im_Vergleich | 14 | 2,0 | 4,0 | 2,786 | ,1547 | ,5789 |
| Gehleistung | 14 | 1,0 | 5,0 | 3,429 | ,3267 | 1,2225 |
| Gültige Werte (Listenweise) | 14 |  |  |  |  |  |

**Geschlecht = 0, Vorher_Nachher = 1**

| **Deskriptive Statistik** | | | | | | |
| --- | --- | --- | --- | --- | --- | --- |
|  | N | Minimum | Maximum | Mittelwert | | Standardabweichung |
|  | Statistik | Statistik | Statistik | Statistik | Standardfehler | Statistik |
| SF36_allgemein | 18 | 2,0 | 4,0 | 2,944 | ,1271 | ,5393 |
| SF36_derzeitig_im_Vergleich | 18 | 2,0 | 5,0 | 3,278 | ,2109 | ,8948 |
| Gehleistung | 18 | 2,0 | 5,0 | 4,222 | ,1906 | ,8085 |
| Gültige Werte (Listenweise) | 18 |  |  |  |  |  |

**Geschlecht = 1, Vorher_Nachher = 0**

| **Deskriptive Statistik** | | | | | | |
| --- | --- | --- | --- | --- | --- | --- |
|  | N | Minimum | Maximum | Mittelwert | | Standardabweichung |
|  | Statistik | Statistik | Statistik | Statistik | Standardfehler | Statistik |
| SF36_allgemein | 23 | 2,0 | 4,0 | 3,130 | ,1143 | ,5481 |
| SF36_derzeitig_im_Vergleich | 23 | 2,0 | 4,0 | 3,174 | ,1495 | ,7168 |
| Gehleistung | 23 | 1,0 | 5,0 | 3,391 | ,2572 | 1,2336 |
| Gültige Werte (Listenweise) | 23 |  |  |  |  |  |

**Geschlecht = 1, Vorher_Nachher = 1**

| **Deskriptive Statistik** | | | | | | |
| --- | --- | --- | --- | --- | --- | --- |
|  | N | Minimum | Maximum | Mittelwert | | Standardabweichung |
|  | Statistik | Statistik | Statistik | Statistik | Standardfehler | Statistik |
| SF36_allgemein | 31 | 1,0 | 4,0 | 2,968 | ,1181 | ,6575 |
| SF36_derzeitig_im_Vergleich | 31 | 1,0 | 5,0 | 3,226 | ,1653 | ,9205 |
| Gehleistung | 31 | 2,0 | 5,0 | 4,065 | ,2018 | 1,1236 |
| Gültige Werte (Listenweise) | 31 |  |  |  |  |  |

**Deskriptive Statistik**

**Geschlecht = 0, ZEITPUNKT = 0**

| **Deskriptive Statistik** | | | | | | |
| --- | --- | --- | --- | --- | --- | --- |
|  | N | Minimum | Maximum | Mittelwert | | Standardabweichung |
|  | Statistik | Statistik | Statistik | Statistik | Standardfehler | Statistik |
| SF36_allgemein | 14 | 2,0 | 4,0 | 2,786 | ,1869 | ,6993 |
| SF36_derzeitig_im_Vergleich | 14 | 2,0 | 4,0 | 2,786 | ,1547 | ,5789 |
| Gehleistung | 14 | 1,0 | 5,0 | 3,429 | ,3267 | 1,2225 |
| Gültige Werte (Listenweise) | 14 |  |  |  |  |  |

**Geschlecht = 0, ZEITPUNKT = 1**

| **Deskriptive Statistik** | | | | | | |
| --- | --- | --- | --- | --- | --- | --- |
|  | N | Minimum | Maximum | Mittelwert | | Standardabweichung |
|  | Statistik | Statistik | Statistik | Statistik | Standardfehler | Statistik |
| SF36_allgemein | 4 | 2,0 | 3,0 | 2,500 | ,2887 | ,5774 |
| SF36_derzeitig_im_Vergleich | 4 | 2,0 | 3,0 | 2,500 | ,2887 | ,5774 |
| Gehleistung | 4 | 4,0 | 4,0 | 4,000 | ,0000 | ,0000 |
| Gültige Werte (Listenweise) | 4 |  |  |  |  |  |

**Geschlecht = 0, ZEITPUNKT = 2**

| **Deskriptive Statistik** | | | | | | |
| --- | --- | --- | --- | --- | --- | --- |
|  | N | Minimum | Maximum | Mittelwert | | Standardabweichung |
|  | Statistik | Statistik | Statistik | Statistik | Standardfehler | Statistik |
| SF36_allgemein | 6 | 3,0 | 4,0 | 3,333 | ,2108 | ,5164 |
| SF36_derzeitig_im_Vergleich | 6 | 3,0 | 5,0 | 3,667 | ,4216 | 1,0328 |
| Gehleistung | 6 | 2,0 | 5,0 | 4,167 | ,5426 | 1,3292 |
| Gültige Werte (Listenweise) | 6 |  |  |  |  |  |

**Geschlecht = 0, ZEITPUNKT = 3**

| **Deskriptive Statistik** | | | | | | |
| --- | --- | --- | --- | --- | --- | --- |
|  | N | Minimum | Maximum | Mittelwert | | Standardabweichung |
|  | Statistik | Statistik | Statistik | Statistik | Standardfehler | Statistik |
| SF36_allgemein | 2 | 3,0 | 3,0 | 3,000 | ,0000 | ,0000 |
| SF36_derzeitig_im_Vergleich | 2 | 4,0 | 5,0 | 4,500 | ,5000 | ,7071 |
| Gehleistung | 2 | 4,0 | 4,0 | 4,000 | ,0000 | ,0000 |
| Gültige Werte (Listenweise) | 2 |  |  |  |  |  |

**Geschlecht = 0, ZEITPUNKT = 4**

| **Deskriptive Statistik** | | | | | | |
| --- | --- | --- | --- | --- | --- | --- |
|  | N | Minimum | Maximum | Mittelwert | | Standardabweichung |
|  | Statistik | Statistik | Statistik | Statistik | Standardfehler | Statistik |
| SF36_allgemein | 6 | 2,0 | 3,0 | 2,833 | ,1667 | ,4082 |
| SF36_derzeitig_im_Vergleich | 6 | 3,0 | 3,0 | 3,000 | ,0000 | ,0000 |
| Gehleistung | 6 | 4,0 | 5,0 | 4,500 | ,2236 | ,5477 |
| Gültige Werte (Listenweise) | 6 |  |  |  |  |  |

**Geschlecht = 1, ZEITPUNKT = 0**

| **Deskriptive Statistik** | | | | | | |
| --- | --- | --- | --- | --- | --- | --- |
|  | N | Minimum | Maximum | Mittelwert | | Standardabweichung |
|  | Statistik | Statistik | Statistik | Statistik | Standardfehler | Statistik |
| SF36_allgemein | 23 | 2,0 | 4,0 | 3,130 | ,1143 | ,5481 |
| SF36_derzeitig_im_Vergleich | 23 | 2,0 | 4,0 | 3,174 | ,1495 | ,7168 |
| Gehleistung | 23 | 1,0 | 5,0 | 3,391 | ,2572 | 1,2336 |
| Gültige Werte (Listenweise) | 23 |  |  |  |  |  |

**Geschlecht = 1, ZEITPUNKT = 1**

| **Deskriptive Statistik** | | | | | | |
| --- | --- | --- | --- | --- | --- | --- |
|  | N | Minimum | Maximum | Mittelwert | | Standardabweichung |
|  | Statistik | Statistik | Statistik | Statistik | Standardfehler | Statistik |
| SF36_allgemein | 9 | 2,0 | 4,0 | 3,000 | ,2357 | ,7071 |
| SF36_derzeitig_im_Vergleich | 9 | 2,0 | 4,0 | 2,889 | ,2003 | ,6009 |
| Gehleistung | 9 | 2,0 | 5,0 | 3,222 | ,4938 | 1,4814 |
| Gültige Werte (Listenweise) | 9 |  |  |  |  |  |

**Geschlecht = 1, ZEITPUNKT = 2**

| **Deskriptive Statistik** | | | | | | |
| --- | --- | --- | --- | --- | --- | --- |
|  | N | Minimum | Maximum | Mittelwert | | Standardabweichung |
|  | Statistik | Statistik | Statistik | Statistik | Standardfehler | Statistik |
| SF36_allgemein | 7 | 3,0 | 4,0 | 3,286 | ,1844 | ,4880 |
| SF36_derzeitig_im_Vergleich | 7 | 3,0 | 4,0 | 3,429 | ,2020 | ,5345 |
| Gehleistung | 7 | 4,0 | 5,0 | 4,286 | ,1844 | ,4880 |
| Gültige Werte (Listenweise) | 7 |  |  |  |  |  |

**Geschlecht = 1, ZEITPUNKT = 3**

| **Deskriptive Statistik** | | | | | | |
| --- | --- | --- | --- | --- | --- | --- |
|  | N | Minimum | Maximum | Mittelwert | | Standardabweichung |
|  | Statistik | Statistik | Statistik | Statistik | Standardfehler | Statistik |
| SF36_allgemein | 8 | 2,0 | 3,0 | 2,875 | ,1250 | ,3536 |
| SF36_derzeitig_im_Vergleich | 8 | 2,0 | 4,0 | 3,125 | ,2950 | ,8345 |
| Gehleistung | 8 | 2,0 | 5,0 | 4,250 | ,3660 | 1,0351 |
| Gültige Werte (Listenweise) | 8 |  |  |  |  |  |

**Geschlecht = 1, ZEITPUNKT = 4**

| **Deskriptive Statistik** | | | | | | |
| --- | --- | --- | --- | --- | --- | --- |
|  | N | Minimum | Maximum | Mittelwert | | Standardabweichung |
|  | Statistik | Statistik | Statistik | Statistik | Standardfehler | Statistik |
| SF36_allgemein | 7 | 1,0 | 4,0 | 2,714 | ,3595 | ,9512 |
| SF36_derzeitig_im_Vergleich | 7 | 1,0 | 5,0 | 3,571 | ,5714 | 1,5119 |
| Gehleistung | 7 | 4,0 | 5,0 | 4,714 | ,1844 | ,4880 |
| Gültige Werte (Listenweise) | 7 |  |  |  |  |  |

**Univariate Varianzanalyse: Vergleich der Zeitpunkten**

**Ergebnis: Keine Unterschiede im SF36_allgemein**

**Ich würde es weglassen, weil Auswertung problematisch wegen ordinalskalierter Variable plus weil Ergebnis uninteressant…**

| **Zwischensubjektfaktoren** | | |
| --- | --- | --- |
|  | | N |
| ZEITPUNKT | 0 | 37 |
|  | 1 | 13 |
|  | 2 | 13 |
|  | 3 | 10 |
|  | 4 | 13 |
| Geschlecht | 0 | 32 |
|  | 1 | 54 |

| **Deskriptive Statistiken** | | | | |
| --- | --- | --- | --- | --- |
| Abhängige Variable: SF36_allgemein | | | | |
| ZEITPUNKT | Geschlecht | Mittelwert | Standardabweichung | N |
| 0 | 0 | 2,786 | ,6993 | 14 |
|  | 1 | 3,130 | ,5481 | 23 |
|  | Gesamt | 3,000 | ,6236 | 37 |
| 1 | 0 | 2,500 | ,5774 | 4 |
|  | 1 | 3,000 | ,7071 | 9 |
|  | Gesamt | 2,846 | ,6887 | 13 |
| 2 | 0 | 3,333 | ,5164 | 6 |
|  | 1 | 3,286 | ,4880 | 7 |
|  | Gesamt | 3,308 | ,4804 | 13 |
| 3 | 0 | 3,000 | ,0000 | 2 |
|  | 1 | 2,875 | ,3536 | 8 |
|  | Gesamt | 2,900 | ,3162 | 10 |
| 4 | 0 | 2,833 | ,4082 | 6 |
|  | 1 | 2,714 | ,9512 | 7 |
|  | Gesamt | 2,769 | ,7250 | 13 |
| Gesamt | 0 | 2,875 | ,6091 | 32 |
|  | 1 | 3,037 | ,6132 | 54 |
|  | Gesamt | 2,977 | ,6131 | 86 |

| **Tests der Zwischensubjekteffekte** | | | | | |
| --- | --- | --- | --- | --- | --- |
| Abhängige Variable: SF36_allgemein | | | | | |
| Quelle | Quadratsumme vom Typ III | df | Mittel der Quadrate | F | Sig. |
| Korrigiertes Modell | 4,089 | 9 | ,454 | 1,239 | ,284 |
| Konstanter Term | 504,494 | 1 | 504,494 | 1375,993 | ,000 |
| ZEITPUNKT | 2,516 | 4 | ,629 | 1,716 | ,155 |
| Geschlecht | ,178 | 1 | ,178 | ,485 | ,488 |
| ZEITPUNKT * Geschlecht | 1,124 | 4 | ,281 | ,766 | ,550 |
| Fehler | 27,865 | 76 | ,367 |  |  |
| Gesamt | 794,000 | 86 |  |  |  |
| Korrigierte Gesamtvariation | 31,953 | 85 |  |  |  |

**Univariate Varianzanalyse**

**Ergebnis: Nur Zeitpunkt 1 vs 3 unterscheiden sich im SF36-Vergleich und das eigentlich auch nur bei Frauen…**

**Ich würde es weglassen, weil Auswertung problematisch wegen ordinalskalierter Variable plus weil Ergebnis fraglich…**

| **Zwischensubjektfaktoren** | | |
| --- | --- | --- |
|  | | N |
| ZEITPUNKT | 0 | 37 |
|  | 1 | 13 |
|  | 2 | 13 |
|  | 3 | 10 |
|  | 4 | 13 |
| Geschlecht | 0 | 32 |
|  | 1 | 54 |

| **Deskriptive Statistiken** | | | | |
| --- | --- | --- | --- | --- |
| Abhängige Variable: SF36_derzeitig_im_Vergleich | | | | |
| ZEITPUNKT | Geschlecht | Mittelwert | Standardabweichung | N |
| 0 | 0 | 2,786 | ,5789 | 14 |
|  | 1 | 3,174 | ,7168 | 23 |
|  | Gesamt | 3,027 | ,6866 | 37 |
| 1 | 0 | 2,500 | ,5774 | 4 |
|  | 1 | 2,889 | ,6009 | 9 |
|  | Gesamt | 2,769 | ,5991 | 13 |
| 2 | 0 | 3,667 | 1,0328 | 6 |
|  | 1 | 3,429 | ,5345 | 7 |
|  | Gesamt | 3,538 | ,7763 | 13 |
| 3 | 0 | 4,500 | ,7071 | 2 |
|  | 1 | 3,125 | ,8345 | 8 |
|  | Gesamt | 3,400 | ,9661 | 10 |
| 4 | 0 | 3,000 | ,0000 | 6 |
|  | 1 | 3,571 | 1,5119 | 7 |
|  | Gesamt | 3,308 | 1,1094 | 13 |
| Gesamt | 0 | 3,063 | ,8007 | 32 |
|  | 1 | 3,204 | ,8328 | 54 |
|  | Gesamt | 3,151 | ,8191 | 86 |

| **Tests der Zwischensubjekteffekte** | | | | | |
| --- | --- | --- | --- | --- | --- |
| Abhängige Variable: SF36_derzeitig_im_Vergleich | | | | | |
| Quelle | Quadratsumme vom Typ III | df | Mittel der Quadrate | F | Sig. |
| Korrigiertes Modell | 11,348 | 9 | 1,261 | 2,097 | ,040 |
| Konstanter Term | 619,384 | 1 | 619,384 | 1030,335 | ,000 |
| ZEITPUNKT | 8,397 | 4 | 2,099 | 3,492 | ,011 |
| Geschlecht | ,041 | 1 | ,041 | ,068 | ,795 |
| ZEITPUNKT * Geschlecht | 5,425 | 4 | 1,356 | 2,256 | ,071 |
| Fehler | 45,687 | 76 | ,601 |  |  |
| Gesamt | 911,000 | 86 |  |  |  |
| Korrigierte Gesamtvariation | 57,035 | 85 |  |  |  |

**Geschätzte Randmittel**

**1. ZEITPUNKT**

| **Paarweise Vergleiche** | | | | | | |
| --- | --- | --- | --- | --- | --- | --- |
| Abhängige Variable: SF36_derzeitig_im_Vergleich | | | | | | |
| (I)ZEITPUNKT | (J)ZEITPUNKT | Mittlere Differenz (I-J) | Standardfehler | Sig. | 95% Konfidenzintervall für die Differenz | |
|  |  |  |  |  | Untergrenze | Obergrenze |
| 0 | 1 | ,285 | ,267 | 1,000 | -,488 | 1,059 |
|  | 2 | -,568 | ,253 | ,275 | -1,298 | ,162 |
|  | 3 | -,833 | ,333 | ,147 | -1,797 | ,131 |
|  | 4 | -,306 | ,253 | 1,000 | -1,036 | ,424 |
| 1 | 0 | -,285 | ,267 | 1,000 | -1,059 | ,488 |
|  | 2 | -,853 | ,317 | ,088 | -1,771 | ,065 |
|  | 3 | -1,118 | ,385 | ,048 | -2,231 | -,005 |
|  | 4 | -,591 | ,317 | ,664 | -1,509 | ,327 |
| 2 | 0 | ,568 | ,253 | ,275 | -,162 | 1,298 |
|  | 1 | ,853 | ,317 | ,088 | -,065 | 1,771 |
|  | 3 | -,265 | ,375 | 1,000 | -1,348 | ,819 |
|  | 4 | ,262 | ,305 | 1,000 | -,620 | 1,144 |
| 3 | 0 | ,833 | ,333 | ,147 | -,131 | 1,797 |
|  | 1 | 1,118 | ,385 | ,048 | ,005 | 2,231 |
|  | 2 | ,265 | ,375 | 1,000 | -,819 | 1,348 |
|  | 4 | ,527 | ,375 | 1,000 | -,557 | 1,610 |
| 4 | 0 | ,306 | ,253 | 1,000 | -,424 | 1,036 |
|  | 1 | ,591 | ,317 | ,664 | -,327 | 1,509 |
|  | 2 | -,262 | ,305 | 1,000 | -1,144 | ,620 |
|  | 3 | -,527 | ,375 | 1,000 | -1,610 | ,557 |

**2. ZEITPUNKT * Geschlecht**

| **Paarweise Vergleiche** | | | | | | | |
| --- | --- | --- | --- | --- | --- | --- | --- |
| Abhängige Variable: SF36_derzeitig_im_Vergleich | | | | | | | |
| Geschlecht | (I)ZEITPUNKT | (J)ZEITPUNKT | Mittlere Differenz (I-J) | Standardfehler | Sig. | 95% Konfidenzintervall für die Differenz | |
|  |  |  |  |  |  | Untergrenze | Obergrenze |
| 0 | 0 | 1 | ,286 | ,440 | 1,000 | -,985 | 1,557 |
|  |  | 2 | -,881 | ,378 | ,225 | -1,975 | ,213 |
|  |  | 3 | -1,714 | ,586 | ,045 | -3,409 | -,020 |
|  |  | 4 | -,214 | ,378 | 1,000 | -1,308 | ,880 |
|  | 1 | 0 | -,286 | ,440 | 1,000 | -1,557 | ,985 |
|  |  | 2 | -1,167 | ,500 | ,224 | -2,614 | ,280 |
|  |  | 3 | -2,000 | ,671 | ,039 | -3,941 | -,059 |
|  |  | 4 | -,500 | ,500 | 1,000 | -1,947 | ,947 |
|  | 2 | 0 | ,881 | ,378 | ,225 | -,213 | 1,975 |
|  |  | 1 | 1,167 | ,500 | ,224 | -,280 | 2,614 |
|  |  | 3 | -,833 | ,633 | 1,000 | -2,664 | ,997 |
|  |  | 4 | ,667 | ,448 | 1,000 | -,628 | 1,961 |
|  | 3 | 0 | 1,714 | ,586 | ,045 | ,020 | 3,409 |
|  |  | 1 | 2,000 | ,671 | ,039 | ,059 | 3,941 |
|  |  | 2 | ,833 | ,633 | 1,000 | -,997 | 2,664 |
|  |  | 4 | 1,500 | ,633 | ,204 | -,330 | 3,330 |
|  | 4 | 0 | ,214 | ,378 | 1,000 | -,880 | 1,308 |
|  |  | 1 | ,500 | ,500 | 1,000 | -,947 | 1,947 |
|  |  | 2 | -,667 | ,448 | 1,000 | -1,961 | ,628 |
|  |  | 3 | -1,500 | ,633 | ,204 | -3,330 | ,330 |
| 1 | 0 | 1 | ,285 | ,305 | 1,000 | -,596 | 1,166 |
|  |  | 2 | -,255 | ,335 | 1,000 | -1,222 | ,713 |
|  |  | 3 | ,049 | ,318 | 1,000 | -,871 | ,969 |
|  |  | 4 | -,398 | ,335 | 1,000 | -1,365 | ,570 |
|  | 1 | 0 | -,285 | ,305 | 1,000 | -1,166 | ,596 |
|  |  | 2 | -,540 | ,391 | 1,000 | -1,669 | ,590 |
|  |  | 3 | -,236 | ,377 | 1,000 | -1,325 | ,853 |
|  |  | 4 | -,683 | ,391 | ,847 | -1,812 | ,447 |
|  | 2 | 0 | ,255 | ,335 | 1,000 | -,713 | 1,222 |
|  |  | 1 | ,540 | ,391 | 1,000 | -,590 | 1,669 |
|  |  | 3 | ,304 | ,401 | 1,000 | -,857 | 1,464 |
|  |  | 4 | -,143 | ,414 | 1,000 | -1,341 | 1,055 |
|  | 3 | 0 | -,049 | ,318 | 1,000 | -,969 | ,871 |
|  |  | 1 | ,236 | ,377 | 1,000 | -,853 | 1,325 |
|  |  | 2 | -,304 | ,401 | 1,000 | -1,464 | ,857 |
|  |  | 4 | -,446 | ,401 | 1,000 | -1,607 | ,714 |
|  | 4 | 0 | ,398 | ,335 | 1,000 | -,570 | 1,365 |
|  |  | 1 | ,683 | ,391 | ,847 | -,447 | 1,812 |
|  |  | 2 | ,143 | ,414 | 1,000 | -1,055 | 1,341 |
|  |  | 3 | ,446 | ,401 | 1,000 | -,714 | 1,607 |

**3. ZEITPUNKT * Geschlecht**

| **Paarweise Vergleiche** | | | | | | | |
| --- | --- | --- | --- | --- | --- | --- | --- |
| Abhängige Variable: SF36_derzeitig_im_Vergleich | | | | | | | |
| ZEITPUNKT | (I)Geschlecht | (J)Geschlecht | Mittlere Differenz (I-J) | Standardfehler | Sig. | 95% Konfidenzintervall für die Differenz | |
|  |  |  |  |  |  | Untergrenze | Obergrenze |
| 0 | 0 | 1 | -,388 | ,263 | ,144 | -,912 | ,135 |
|  | 1 | 0 | ,388 | ,263 | ,144 | -,135 | ,912 |
| 1 | 0 | 1 | -,389 | ,466 | ,407 | -1,317 | ,539 |
|  | 1 | 0 | ,389 | ,466 | ,407 | -,539 | 1,317 |
| 2 | 0 | 1 | ,238 | ,431 | ,583 | -,621 | 1,097 |
|  | 1 | 0 | -,238 | ,431 | ,583 | -1,097 | ,621 |
| 3 | 0 | 1 | 1,375 | ,613 | ,028 | ,154 | 2,596 |
|  | 1 | 0 | -1,375 | ,613 | ,028 | -2,596 | -,154 |
| 4 | 0 | 1 | -,571 | ,431 | ,189 | -1,431 | ,288 |
|  | 1 | 0 | ,571 | ,431 | ,189 | -,288 | 1,431 |

**Univariate Varianzanalyse**

Bei Gehleistung Unterschied Zeitpunkte 0 vs 4, sonst keine…aber auch hier problematisch…

| **Zwischensubjektfaktoren** | | |
| --- | --- | --- |
|  | | N |
| ZEITPUNKT | 0 | 37 |
|  | 1 | 13 |
|  | 2 | 13 |
|  | 3 | 10 |
|  | 4 | 13 |
| Geschlecht | 0 | 32 |
|  | 1 | 54 |

| **Deskriptive Statistiken** | | | | |
| --- | --- | --- | --- | --- |
| Abhängige Variable: Gehleistung | | | | |
| ZEITPUNKT | Geschlecht | Mittelwert | Standardabweichung | N |
| 0 | 0 | 3,429 | 1,2225 | 14 |
|  | 1 | 3,391 | 1,2336 | 23 |
|  | Gesamt | 3,405 | 1,2124 | 37 |
| 1 | 0 | 4,000 | ,0000 | 4 |
|  | 1 | 3,222 | 1,4814 | 9 |
|  | Gesamt | 3,462 | 1,2659 | 13 |
| 2 | 0 | 4,167 | 1,3292 | 6 |
|  | 1 | 4,286 | ,4880 | 7 |
|  | Gesamt | 4,231 | ,9268 | 13 |
| 3 | 0 | 4,000 | ,0000 | 2 |
|  | 1 | 4,250 | 1,0351 | 8 |
|  | Gesamt | 4,200 | ,9189 | 10 |
| 4 | 0 | 4,500 | ,5477 | 6 |
|  | 1 | 4,714 | ,4880 | 7 |
|  | Gesamt | 4,615 | ,5064 | 13 |
| Gesamt | 0 | 3,875 | 1,0701 | 32 |
|  | 1 | 3,778 | 1,2079 | 54 |
|  | Gesamt | 3,814 | 1,1531 | 86 |

| **Tests der Zwischensubjekteffekte** | | | | | |
| --- | --- | --- | --- | --- | --- |
| Abhängige Variable: Gehleistung | | | | | |
| Quelle | Quadratsumme vom Typ III | df | Mittel der Quadrate | F | Sig. |
| Korrigiertes Modell | 21,870 | 9 | 2,430 | 2,026 | ,048 |
| Konstanter Term | 928,280 | 1 | 928,280 | 773,967 | ,000 |
| ZEITPUNKT | 17,064 | 4 | 4,266 | 3,557 | ,010 |
| Geschlecht | ,031 | 1 | ,031 | ,026 | ,872 |
| ZEITPUNKT * Geschlecht | 1,930 | 4 | ,483 | ,402 | ,806 |
| Fehler | 91,153 | 76 | 1,199 |  |  |
| Gesamt | 1364,000 | 86 |  |  |  |
| Korrigierte Gesamtvariation | 113,023 | 85 |  |  |  |

**Geschätzte Randmittel**

**1. ZEITPUNKT**

| **Paarweise Vergleiche** | | | | | | |
| --- | --- | --- | --- | --- | --- | --- |
| Abhängige Variable: Gehleistung | | | | | | |
| (I)ZEITPUNKT | (J)ZEITPUNKT | Mittlere Differenz (I-J) | Standardfehler | Sig. | 95% Konfidenzintervall für die Differenz | |
|  |  |  |  |  | Untergrenze | Obergrenze |
| 0 | 1 | -,201 | ,378 | 1,000 | -1,293 | ,891 |
|  | 2 | -,816 | ,357 | ,249 | -1,848 | ,215 |
|  | 3 | -,715 | ,471 | 1,000 | -2,077 | ,647 |
|  | 4 | -1,197 | ,357 | ,012 | -2,229 | -,166 |
| 1 | 0 | ,201 | ,378 | 1,000 | -,891 | 1,293 |
|  | 2 | -,615 | ,448 | 1,000 | -1,912 | ,681 |
|  | 3 | -,514 | ,544 | 1,000 | -2,086 | 1,058 |
|  | 4 | -,996 | ,448 | ,293 | -2,293 | ,301 |
| 2 | 0 | ,816 | ,357 | ,249 | -,215 | 1,848 |
|  | 1 | ,615 | ,448 | 1,000 | -,681 | 1,912 |
|  | 3 | ,101 | ,529 | 1,000 | -1,429 | 1,632 |
|  | 4 | -,381 | ,431 | 1,000 | -1,627 | ,865 |
| 3 | 0 | ,715 | ,471 | 1,000 | -,647 | 2,077 |
|  | 1 | ,514 | ,544 | 1,000 | -1,058 | 2,086 |
|  | 2 | -,101 | ,529 | 1,000 | -1,632 | 1,429 |
|  | 4 | -,482 | ,529 | 1,000 | -2,013 | 1,048 |
| 4 | 0 | 1,197 | ,357 | ,012 | ,166 | 2,229 |
|  | 1 | ,996 | ,448 | ,293 | -,301 | 2,293 |
|  | 2 | ,381 | ,431 | 1,000 | -,865 | 1,627 |
|  | 3 | ,482 | ,529 | 1,000 | -1,048 | 2,013 |
