# Supplementary material for: Intra-articular injection of bone marrow aspirate concentrate (mesenchymal stem cells) in KL grade III and IV knee osteoarthritis: 4 year results of 37 knees
Source: Sci Rep. 2024 Feb 1;14:2665. doi: 10.1038/s41598-024-51410-2 (PMC10834500; doi:10.1038/s41598-024-51410-2)
Supplement: Supplementary file 7 — Supplementary Information 7. [file 41598_2024_51410_MOESM7_ESM.docx]

95%iges Konfidenzintervall: Immer MITTELWERT – STANDARDFEHLER für Untergrenze und MITTELWERT + STANDARDFEHLER für Obergrenze

**WOMAC – paired: Gesamt**

| **Deskriptive Statistik** | | | | | | |
| --- | --- | --- | --- | --- | --- | --- |
|  | N | Minimum | Maximum | Mittelwert | | Standardabweichung |
|  | Statistik | Statistik | Statistik | Statistik | Standardfehler | Statistik |
| t0_WOMAC-SCORE | 37 | 6 | 96 | 39,73 | 3,744 | 22,773 |
| t1_WOMAC-SCORE | 37 | 0 | 67 | 18,51 | 2,990 | 18,188 |
| Gültige Werte (Listenweise) | 37 |  |  |  |  |  |

- zB Konfidenzintervall für t0 ist 35,986 bis 43,474 um den Mittelwert von 39,73

**WOMAC – paired: Getrennt nach Geschlecht**

**t0_Geschlecht = 0**

| **Deskriptive Statistik** | | | | | | |
| --- | --- | --- | --- | --- | --- | --- |
|  | N | Minimum | Maximum | Mittelwert | | Standardabweichung |
|  | Statistik | Statistik | Statistik | Statistik | Standardfehler | Statistik |
| t0_WOMAC-SCORE | 14 | 6 | 96 | 45,43 | 8,061 | 30,160 |
| t1_WOMAC-SCORE | 14 | 1 | 50 | 17,57 | 4,377 | 16,379 |
| Gültige Werte (Listenweise) | 14 |  |  |  |  |  |

**t0_Geschlecht = 1**

| **Deskriptive Statistik** | | | | | | |
| --- | --- | --- | --- | --- | --- | --- |
|  | N | Minimum | Maximum | Mittelwert | | Standardabweichung |
|  | Statistik | Statistik | Statistik | Statistik | Standardfehler | Statistik |
| t0_WOMAC-SCORE | 23 | 10 | 79 | 36,26 | 3,476 | 16,668 |
| t1_WOMAC-SCORE | 23 | 0 | 67 | 19,09 | 4,075 | 19,542 |
| Gültige Werte (Listenweise) | 23 |  |  |  |  |  |

**WOMAC – unpaired: Gesamt**

| **Deskriptive Statistik** | | | | | | |
| --- | --- | --- | --- | --- | --- | --- |
|  | N | Minimum | Maximum | Mittelwert | | Standardabweichung |
|  | Statistik | Statistik | Statistik | Statistik | Standardfehler | Statistik |
| WOMAC-SCORE | 87 | 0 | 96 | 27,24 | 2,418 | 22,556 |
| Gültige Werte (Listenweise) | 87 |  |  |  |  |  |

**WOMAC – unpaired: Getrennt nach Geschlecht**

**Geschlecht = 0**

| **Deskriptive Statistik** | | | | | | |
| --- | --- | --- | --- | --- | --- | --- |
|  | N | Minimum | Maximum | Mittelwert | | Standardabweichung |
|  | Statistik | Statistik | Statistik | Statistik | Standardfehler | Statistik |
| WOMAC-SCORE | 37 | 0 | 96 | 27,08 | 4,261 | 25,918 |
| Gültige Werte (Listenweise) | 37 |  |  |  |  |  |

**Geschlecht = 1**

| **Deskriptive Statistik** | | | | | | |
| --- | --- | --- | --- | --- | --- | --- |
|  | N | Minimum | Maximum | Mittelwert | | Standardabweichung |
|  | Statistik | Statistik | Statistik | Statistik | Standardfehler | Statistik |
| WOMAC-SCORE | 50 | 0 | 79 | 27,36 | 2,826 | 19,984 |
| Gültige Werte (Listenweise) | 50 |  |  |  |  |  |

**WOMAC – unpaired: Getrennt nach Vorher_Nachher**

**Vorher_Nachher = 0**

| **Deskriptive Statistik** | | | | | | |
| --- | --- | --- | --- | --- | --- | --- |
|  | N | Minimum | Maximum | Mittelwert | | Standardabweichung |
|  | Statistik | Statistik | Statistik | Statistik | Standardfehler | Statistik |
| WOMAC-SCORE | 37 | 6 | 96 | 39,73 | 3,744 | 22,773 |
| Gültige Werte (Listenweise) | 37 |  |  |  |  |  |

**Vorher_Nachher = 1**

| **Deskriptive Statistik** | | | | | | |
| --- | --- | --- | --- | --- | --- | --- |
|  | N | Minimum | Maximum | Mittelwert | | Standardabweichung |
|  | Statistik | Statistik | Statistik | Statistik | Standardfehler | Statistik |
| WOMAC-SCORE | 50 | 0 | 67 | 18,00 | 2,478 | 17,522 |
| Gültige Werte (Listenweise) | 50 |  |  |  |  |  |

**WOMAC – unpaired: Getrennt nach Zeitpunkt**

**Zeitpunkt = 0**

| **Deskriptive Statistik** | | | | | | |
| --- | --- | --- | --- | --- | --- | --- |
|  | N | Minimum | Maximum | Mittelwert | | Standardabweichung |
|  | Statistik | Statistik | Statistik | Statistik | Standardfehler | Statistik |
| WOMAC-SCORE | 37 | 6 | 96 | 39,73 | 3,744 | 22,773 |
| Gültige Werte (Listenweise) | 37 |  |  |  |  |  |

**Zeitpunkt = 1**

| **Deskriptive Statistik** | | | | | | |
| --- | --- | --- | --- | --- | --- | --- |
|  | N | Minimum | Maximum | Mittelwert | | Standardabweichung |
|  | Statistik | Statistik | Statistik | Statistik | Standardfehler | Statistik |
| WOMAC-SCORE | 20 | 0 | 67 | 25,05 | 4,487 | 20,067 |
| Gültige Werte (Listenweise) | 20 |  |  |  |  |  |

**Zeitpunkt = 2**

| **Deskriptive Statistik** | | | | | | |
| --- | --- | --- | --- | --- | --- | --- |
|  | N | Minimum | Maximum | Mittelwert | | Standardabweichung |
|  | Statistik | Statistik | Statistik | Statistik | Standardfehler | Statistik |
| WOMAC-SCORE | 10 | 0 | 25 | 14,20 | 3,108 | 9,830 |
| Gültige Werte (Listenweise) | 10 |  |  |  |  |  |

**Zeitpunkt = 3**

| **Deskriptive Statistik** | | | | | | |
| --- | --- | --- | --- | --- | --- | --- |
|  | N | Minimum | Maximum | Mittelwert | | Standardabweichung |
|  | Statistik | Statistik | Statistik | Statistik | Standardfehler | Statistik |
| WOMAC-SCORE | 8 | 2 | 58 | 12,38 | 6,697 | 18,943 |
| Gültige Werte (Listenweise) | 8 |  |  |  |  |  |

**Zeitpunkt = 4**

| **Deskriptive Statistik** | | | | | | |
| --- | --- | --- | --- | --- | --- | --- |
|  | N | Minimum | Maximum | Mittelwert | | Standardabweichung |
|  | Statistik | Statistik | Statistik | Statistik | Standardfehler | Statistik |
| WOMAC-SCORE | 12 | 0 | 53 | 13,17 | 4,210 | 14,584 |
| Gültige Werte (Listenweise) | 12 |  |  |  |  |  |

**WOMAC – unpaired: Getrennt nach Geschlecht und Vorher_Nachher**

**Geschlecht = 0, Vorher_Nachher = 0**

| **Deskriptive Statistik** | | | | | | |
| --- | --- | --- | --- | --- | --- | --- |
|  | N | Minimum | Maximum | Mittelwert | | Standardabweichung |
|  | Statistik | Statistik | Statistik | Statistik | Standardfehler | Statistik |
| WOMAC-SCORE | 14 | 6 | 96 | 45,43 | 8,061 | 30,160 |
| Gültige Werte (Listenweise) | 14 |  |  |  |  |  |

**Geschlecht = 0, Vorher_Nachher = 1**

| **Deskriptive Statistik** | | | | | | |
| --- | --- | --- | --- | --- | --- | --- |
|  | N | Minimum | Maximum | Mittelwert | | Standardabweichung |
|  | Statistik | Statistik | Statistik | Statistik | Standardfehler | Statistik |
| WOMAC-SCORE | 23 | 0 | 50 | 15,91 | 3,072 | 14,734 |
| Gültige Werte (Listenweise) | 23 |  |  |  |  |  |

**Geschlecht = 1, Vorher_Nachher = 0**

| **Deskriptive Statistik** | | | | | | |
| --- | --- | --- | --- | --- | --- | --- |
|  | N | Minimum | Maximum | Mittelwert | | Standardabweichung |
|  | Statistik | Statistik | Statistik | Statistik | Standardfehler | Statistik |
| WOMAC-SCORE | 23 | 10 | 79 | 36,26 | 3,476 | 16,668 |
| Gültige Werte (Listenweise) | 23 |  |  |  |  |  |

**Geschlecht = 1, Vorher_Nachher = 1**

| **Deskriptive Statistik** | | | | | | |
| --- | --- | --- | --- | --- | --- | --- |
|  | N | Minimum | Maximum | Mittelwert | | Standardabweichung |
|  | Statistik | Statistik | Statistik | Statistik | Standardfehler | Statistik |
| WOMAC-SCORE | 27 | 0 | 67 | 19,78 | 3,790 | 19,693 |
| Gültige Werte (Listenweise) | 27 |  |  |  |  |  |

**WOMAC – unpaired: Getrennt nach Geschlecht und Zeitpunkt**

**Geschlecht = 0, Zeitpunkt = 0**

| **Deskriptive Statistik** | | | | | | |
| --- | --- | --- | --- | --- | --- | --- |
|  | N | Minimum | Maximum | Mittelwert | | Standardabweichung |
|  | Statistik | Statistik | Statistik | Statistik | Standardfehler | Statistik |
| WOMAC-SCORE | 14 | 6 | 96 | 45,43 | 8,061 | 30,160 |
| Gültige Werte (Listenweise) | 14 |  |  |  |  |  |

**Geschlecht = 0, Zeitpunkt = 1**

| **Deskriptive Statistik** | | | | | | |
| --- | --- | --- | --- | --- | --- | --- |
|  | N | Minimum | Maximum | Mittelwert | | Standardabweichung |
|  | Statistik | Statistik | Statistik | Statistik | Standardfehler | Statistik |
| WOMAC-SCORE | 12 | 0 | 50 | 19,08 | 5,138 | 17,799 |
| Gültige Werte (Listenweise) | 12 |  |  |  |  |  |

**Geschlecht = 0, Zeitpunkt = 2**

| **Deskriptive Statistik** | | | | | | |
| --- | --- | --- | --- | --- | --- | --- |
|  | N | Minimum | Maximum | Mittelwert | | Standardabweichung |
|  | Statistik | Statistik | Statistik | Statistik | Standardfehler | Statistik |
| WOMAC-SCORE | 4 | 0 | 25 | 18,25 | 6,102 | 12,203 |
| Gültige Werte (Listenweise) | 4 |  |  |  |  |  |

**Geschlecht = 0, Zeitpunkt = 3**

| **Deskriptive Statistik** | | | | | | |
| --- | --- | --- | --- | --- | --- | --- |
|  | N | Minimum | Maximum | Mittelwert | | Standardabweichung |
|  | Statistik | Statistik | Statistik | Statistik | Standardfehler | Statistik |
| WOMAC-SCORE | 1 | 3 | 3 | 3,00 | . | . |
| Gültige Werte (Listenweise) | 1 |  |  |  |  |  |

**Geschlecht = 0, Zeitpunkt = 4**

| **Deskriptive Statistik** | | | | | | |
| --- | --- | --- | --- | --- | --- | --- |
|  | N | Minimum | Maximum | Mittelwert | | Standardabweichung |
|  | Statistik | Statistik | Statistik | Statistik | Standardfehler | Statistik |
| WOMAC-SCORE | 6 | 2 | 23 | 10,17 | 3,351 | 8,208 |
| Gültige Werte (Listenweise) | 6 |  |  |  |  |  |

**Geschlecht = 1, Zeitpunkt = 0**

| **Deskriptive Statistik** | | | | | | |
| --- | --- | --- | --- | --- | --- | --- |
|  | N | Minimum | Maximum | Mittelwert | | Standardabweichung |
|  | Statistik | Statistik | Statistik | Statistik | Standardfehler | Statistik |
| WOMAC-SCORE | 23 | 10 | 79 | 36,26 | 3,476 | 16,668 |
| Gültige Werte (Listenweise) | 23 |  |  |  |  |  |

**Geschlecht = 1, Zeitpunkt = 1**

| **Deskriptive Statistik** | | | | | | |
| --- | --- | --- | --- | --- | --- | --- |
|  | N | Minimum | Maximum | Mittelwert | | Standardabweichung |
|  | Statistik | Statistik | Statistik | Statistik | Standardfehler | Statistik |
| WOMAC-SCORE | 8 | 7 | 67 | 34,00 | 7,438 | 21,037 |
| Gültige Werte (Listenweise) | 8 |  |  |  |  |  |

**Geschlecht = 1, Zeitpunkt = 2**

| **Deskriptive Statistik** | | | | | | |
| --- | --- | --- | --- | --- | --- | --- |
|  | N | Minimum | Maximum | Mittelwert | | Standardabweichung |
|  | Statistik | Statistik | Statistik | Statistik | Standardfehler | Statistik |
| WOMAC-SCORE | 6 | 0 | 23 | 11,50 | 3,233 | 7,918 |
| Gültige Werte (Listenweise) | 6 |  |  |  |  |  |

**Geschlecht = 1, Zeitpunkt = 3**

| **Deskriptive Statistik** | | | | | | |
| --- | --- | --- | --- | --- | --- | --- |
|  | N | Minimum | Maximum | Mittelwert | | Standardabweichung |
|  | Statistik | Statistik | Statistik | Statistik | Standardfehler | Statistik |
| WOMAC-SCORE | 7 | 2 | 58 | 13,71 | 7,577 | 20,048 |
| Gültige Werte (Listenweise) | 7 |  |  |  |  |  |

**Geschlecht = 1, Zeitpunkt = 4**

| **Deskriptive Statistik** | | | | | | |
| --- | --- | --- | --- | --- | --- | --- |
|  | N | Minimum | Maximum | Mittelwert | | Standardabweichung |
|  | Statistik | Statistik | Statistik | Statistik | Standardfehler | Statistik |
| WOMAC-SCORE | 6 | 0 | 53 | 16,17 | 7,947 | 19,467 |
| Gültige Werte (Listenweise) | 6 |  |  |  |  |  |

**Univariate Varianzanalyse: Vergleich zwischen Zeitpunkten und Geschlechter (unpaired)**

| **Zwischensubjektfaktoren** | | |
| --- | --- | --- |
|  | | N |
| Zeitpunkt | 0 | 37 |
|  | 1 | 20 |
|  | 2 | 10 |
|  | 3 | 8 |
|  | 4 | 12 |
| Geschlecht | 0 | 37 |
|  | 1 | 50 |

| **Deskriptive Statistiken** | | | | |
| --- | --- | --- | --- | --- |
| Abhängige Variable: WOMAC-SCORE | | | | |
| Zeitpunkt | Geschlecht | Mittelwert | Standardabweichung | N |
| 0 | 0 | 45,43 | 30,160 | 14 |
|  | 1 | 36,26 | 16,668 | 23 |
|  | Gesamt | 39,73 | 22,773 | 37 |
| 1 | 0 | 19,08 | 17,799 | 12 |
|  | 1 | 34,00 | 21,037 | 8 |
|  | Gesamt | 25,05 | 20,067 | 20 |
| 2 | 0 | 18,25 | 12,203 | 4 |
|  | 1 | 11,50 | 7,918 | 6 |
|  | Gesamt | 14,20 | 9,830 | 10 |
| 3 | 0 | 3,00 | . | 1 |
|  | 1 | 13,71 | 20,048 | 7 |
|  | Gesamt | 12,38 | 18,943 | 8 |
| 4 | 0 | 10,17 | 8,208 | 6 |
|  | 1 | 16,17 | 19,467 | 6 |
|  | Gesamt | 13,17 | 14,584 | 12 |
| Gesamt | 0 | 27,08 | 25,918 | 37 |
|  | 1 | 27,36 | 19,984 | 50 |
|  | Gesamt | 27,24 | 22,556 | 87 |

| **Tests der Zwischensubjekteffekte** | | | | | |
| --- | --- | --- | --- | --- | --- |
| Abhängige Variable: WOMAC-SCORE | | | | | |
| Quelle | Quadratsumme vom Typ III | df | Mittel der Quadrate | F | Sig. |
| Korrigiertes Modell | 13829,806 | 9 | 1536,645 | 3,954 | ,000 |
| Konstanter Term | 19442,047 | 1 | 19442,047 | 50,028 | ,000 |
| Zeitpunkt | 11401,492 | 4 | 2850,373 | 7,335 | ,000 |
| Geschlecht | 111,415 | 1 | 111,415 | ,287 | ,594 |
| Zeitpunkt * Geschlecht | 2116,811 | 4 | 529,203 | 1,362 | ,255 |
| Fehler | 29924,125 | 77 | 388,625 |  |  |
| Gesamt | 108316,000 | 87 |  |  |  |
| Korrigierte Gesamtvariation | 43753,931 | 86 |  |  |  |

**Geschätzte Randmittel**

| **Paarweise Vergleiche** | | | | | | |
| --- | --- | --- | --- | --- | --- | --- |
| Abhängige Variable: WOMAC-SCORE | | | | | | |
| (I)Zeitpunkt | (J)Zeitpunkt | Mittlere Differenz (I-J) | Standardfehler | Sig. | 95% Konfidenzintervall für die Differenz | |
|  |  |  |  |  | Untergrenze | Obergrenze |
| 0 | 1 | 14,303 | 5,604 | ,127 | -1,893 | 30,500 |
|  | 2 | 25,970 | 7,186 | ,005 | 5,200 | 46,740 |
|  | 3 | 32,488 | 11,054 | ,043 | ,538 | 64,437 |
|  | 4 | 27,678 | 6,599 | ,001 | 8,605 | 46,751 |
| 1 | 0 | -14,303 | 5,604 | ,127 | -30,500 | 1,893 |
|  | 2 | 11,667 | 7,792 | 1,000 | -10,855 | 34,188 |
|  | 3 | 18,185 | 11,458 | 1,000 | -14,930 | 51,299 |
|  | 4 | 13,375 | 7,254 | ,691 | -7,591 | 34,341 |
| 2 | 0 | -25,970 | 7,186 | ,005 | -46,740 | -5,200 |
|  | 1 | -11,667 | 7,792 | 1,000 | -34,188 | 10,855 |
|  | 3 | 6,518 | 12,309 | 1,000 | -29,058 | 42,094 |
|  | 4 | 1,708 | 8,536 | 1,000 | -22,963 | 26,379 |
| 3 | 0 | -32,488 | 11,054 | ,043 | -64,437 | -,538 |
|  | 1 | -18,185 | 11,458 | 1,000 | -51,299 | 14,930 |
|  | 2 | -6,518 | 12,309 | 1,000 | -42,094 | 29,058 |
|  | 4 | -4,810 | 11,976 | 1,000 | -39,422 | 29,803 |
| 4 | 0 | -27,678 | 6,599 | ,001 | -46,751 | -8,605 |
|  | 1 | -13,375 | 7,254 | ,691 | -34,341 | 7,591 |
|  | 2 | -1,708 | 8,536 | 1,000 | -26,379 | 22,963 |
|  | 3 | 4,810 | 11,976 | 1,000 | -29,803 | 39,422 |
