# Supplementary material for: Intra-articular injection of bone marrow aspirate concentrate (mesenchymal stem cells) in KL grade III and IV knee osteoarthritis: 4 year results of 37 knees
Source: Sci Rep. 2024 Feb 1;14:2665. doi: 10.1038/s41598-024-51410-2 (PMC10834500; doi:10.1038/s41598-024-51410-2)
Supplement: Supplementary file 8 — Supplementary Information 8. [file 41598_2024_51410_MOESM8_ESM.docx]

WOMAC-Score_unpaired: Vergleich zwischen vorher und nachher (alle)

**Korrelationen**

Zur Kontrolle der „Interessensvariablen“…

Über alle Zeitpunkte: WOMAC und Alter sind korreliert (weglassen, verwirrt nur...verändert nichts an Aussage, weil, Der Unterschied vorher / nachher nicht mit Alter korrreliert)

WOMAC mit Größe, Gewicht und BMI nicht

| **Korrelationen** | | | | | | |
| --- | --- | --- | --- | --- | --- | --- |
|  | | WOMAC-SCORE | Größe | Gewicht | BMI | Alter |
| WOMAC-SCORE | Korrelation nach Pearson | 1 | -,030 | 7,064 | ,099 | ,297 |
|  | Signifikanz (2-seitig) |  | ,780 | ,559 | ,361 | ,006 |
|  | N | 87 | 87 | 87 | 87 | 86 |
| Größe | Korrelation nach Pearson | -,030 | 1 | ,821 | ,475 | -,262 |
|  | Signifikanz (2-seitig) | ,780 |  | ,000 | ,000 | ,015 |
|  | N | 87 | 87 | 87 | 87 | 86 |
| Gewicht | Korrelation nach Pearson | ,064 | ,821 | 1 | ,889 | -,154 |
|  | Signifikanz (2-seitig) | ,559 | ,000 |  | ,000 | ,158 |
|  | N | 87 | 87 | 87 | 87 | 86 |
| BMI | Korrelation nach Pearson | ,099 | ,475 | ,889 | 1 | -,017 |
|  | Signifikanz (2-seitig) | ,361 | ,000 | ,000 |  | ,874 |
|  | N | 87 | 87 | 87 | 87 | 86 |
| Alter | Korrelation nach Pearson | ,297 | -,262 | -,154 | -,017 | 1 |
|  | Signifikanz (2-seitig) | ,006 | ,015 | ,158 | ,874 |  |
|  | N | 86 | 86 | 86 | 86 | 86 |

**Nichtparametrische Korrelationen**

| **Korrelationen** | | | | |
| --- | --- | --- | --- | --- |
|  | | | WOMAC-SCORE | Zeitpunkt |
| Spearman-Rho | WOMAC-SCORE | Korrelationskoeffizient | 1,000 | -,553 |
|  |  | Sig. (2-seitig) | . | ,000 |
|  |  | N | 87 | 87 |
|  | Zeitpunkt | Korrelationskoeffizient | -,553 | 1,000 |
|  |  | Sig. (2-seitig) | ,000 | . |
|  |  | N | 87 | 87 |

WOMAC und Zeitpunkt: Je höher Zeitpunkt/Jahre, desto geringer ist WOMAC

**Korrelationen**

**Vorher_Nachher = 0 – dh nur Zeitpunkt „Vorher“**

| **Korrelationen** | | | | | | |
| --- | --- | --- | --- | --- | --- | --- |
|  | | WOMAC-SCORE | Größe | Gewicht | BMI | Alter |
| WOMAC-SCORE | Korrelation nach Pearson | 1 | -,254 | -,087 | ,049 | ,392 |
|  | Signifikanz (2-seitig) |  | ,130 | ,609 | ,774 | ,018 |
|  | N | 37 | 37 | 37 | 37 | 36 |
| Größe | Korrelation nach Pearson | -,254 | 1 | ,841 | ,510 | -,288 |
|  | Signifikanz (2-seitig) | ,130 |  | ,000 | ,001 | ,088 |
|  | N | 37 | 37 | 37 | 37 | 36 |
| Gewicht | Korrelation nach Pearson | -,087 | ,841 | 1 | ,891 | -,207 |
|  | Signifikanz (2-seitig) | ,609 | ,000 |  | ,000 | ,226 |
|  | N | 37 | 37 | 37 | 37 | 36 |
| BMI | Korrelation nach Pearson | ,049 | ,510 | ,891 | 1 | -,068 |
|  | Signifikanz (2-seitig) | ,774 | ,001 | ,000 |  | ,692 |
|  | N | 37 | 37 | 37 | 37 | 36 |
| Alter | Korrelation nach Pearson | ,392 | -,288 | -,207 | -,068 | 1 |
|  | Signifikanz (2-seitig) | ,018 | ,088 | ,226 | ,692 |  |
|  | N | 36 | 36 | 36 | 36 | 36 |

**Vorher_Nachher = 1 – dh nur Nachher-Zeitpunkte (alle)**

| **Korrelationen** | | | | | | |
| --- | --- | --- | --- | --- | --- | --- |
|  | | WOMAC-SCORE | Größe | Gewicht | BMI | Alter |
| WOMAC-SCORE | Korrelation nach Pearson | 1 | ,128 | ,166 | ,129 | ,286 |
|  | Signifikanz (2-seitig) |  | ,375 | ,249 | ,374 | ,044 |
|  | N | 50 | 50 | 50 | 50 | 50 |
| Größe | Korrelation nach Pearson | ,128 | 1 | ,804 | ,447 | -,241 |
|  | Signifikanz (2-seitig) | ,375 |  | ,000 | ,001 | ,092 |
|  | N | 50 | 50 | 50 | 50 | 50 |
| Gewicht | Korrelation nach Pearson | ,166 | ,804 | 1 | ,888 | -,115 |
|  | Signifikanz (2-seitig) | ,249 | ,000 |  | ,000 | ,428 |
|  | N | 50 | 50 | 50 | 50 | 50 |
| BMI | Korrelation nach Pearson | ,129 | ,447 | ,888 | 1 | ,018 |
|  | Signifikanz (2-seitig) | ,374 | ,001 | ,000 |  | ,901 |
|  | N | 50 | 50 | 50 | 50 | 50 |
| Alter | Korrelation nach Pearson | ,286 | -,241 | -,115 | ,018 | 1 |
|  | Signifikanz (2-seitig) | ,044 | ,092 | ,428 | ,901 |  |
|  | N | 50 | 50 | 50 | 50 | 50 |

**Nichtparametrische Korrelationen**

**Vorher_Nachher = 1**

| **Korrelationen** | | | | |
| --- | --- | --- | --- | --- |
|  | | | WOMAC-SCORE | Zeitpunkt |
| Spearman-Rho | WOMAC-SCORE | Korrelationskoeffizient | 1,000 | -,283 |
|  |  | Sig. (2-seitig) | . | ,046 |
|  |  | N | 50 | 50 |
|  | Zeitpunkt | Korrelationskoeffizient | -,283 | 1,000 |
|  |  | Sig. (2-seitig) | ,046 | . |
|  |  | N | 50 | 50 |

Auch getrennt nach Vorher bzw. Nachher die gleichartigen Korrelationen.

**Nichtparametrische Tests: Kontrolle der Verteilungsform für folgende t-Tests/Varianzanalysen**

**Vorher_Nachher = 0**

| **Kolmogorov-Smirnov-Anpassungstest** | | |
| --- | --- | --- |
|  | | WOMAC-SCORE |
| N | | 37 |
| Parameter der Normalverteilung | Mittelwert | 39,73 |
|  | Standardabweichung | 22,773 |
| Extremste Differenzen | Absolut | ,159 |
|  | Positiv | ,159 |
|  | Negativ | -,105 |
| Kolmogorov-Smirnov-Z | | ,964 |
| Asymptotische Signifikanz (2-seitig) | | ,310 |

- WOMAC ist Vorher normalverteilt (weil KS-Test nicht signifikant), passt also für die Analysen

**Vorher_Nachher = 1**

| **Kolmogorov-Smirnov-Anpassungstest** | | |
| --- | --- | --- |
|  | | WOMAC-SCORE |
| N | | 50 |
| Parameter der Normalverteilung | Mittelwert | 18,00 |
|  | Standardabweichung | 17,522 |
| Extremste Differenzen | Absolut | ,168 |
|  | Positiv | ,168 |
|  | Negativ | -,152 |
| Kolmogorov-Smirnov-Z | | 1,188 |
| Asymptotische Signifikanz (2-seitig) | | ,119 |

- WOMAC ist Nachher normalverteilt (weil KS-Test nicht signifikant), passt also für die Analysen

**T-Test: Vergleich Vorher vs. Nachher**

| **Gruppenstatistiken** | | | | | |
| --- | --- | --- | --- | --- | --- |
|  | Vorher_Nachher | N | Mittelwert | Standardabweichung | Standardfehler des Mittelwertes |
| WOMAC-SCORE | 0 | 37 | 39,73 | 22,773 | 3,744 |
|  | 1 | 50 | 18,00 | 17,522 | 2,478 |

| **Test bei unabhängigen Stichproben** | | | | | | | | | | |
| --- | --- | --- | --- | --- | --- | --- | --- | --- | --- | --- |
|  | | Levene-Test der Varianzgleichheit | | T-Test für die Mittelwertgleichheit | | | | | | |
|  |  | F | Signifikanz | **T** | **df** | **Sig. (2-seitig)** | Mittlere Differenz | Standardfehler der Differenz | 95% Konfidenzintervall der Differenz | |
|  |  |  |  |  |  |  |  |  | Untere | Obere |
| WOMAC-SCORE | Varianzen sind gleich | 1,113 | ,294 | **5,031** | **85** | **,000** | 21,730 | 4,319 | 13,143 | 30,317 |
|  |  |  |  |  |  |  |  |  |  |  |

- Signifikanter Unterschied zwischen Vorher und Nachher (alle Zeitpunkte zusammen)
- Mittelwert von 39,7 auf 18,0 gesunken

**Univariate Varianzanalyse: Vergleich inkl. Geschlecht**

| **Zwischensubjektfaktoren** | | |
| --- | --- | --- |
|  | | N |
| Vorher_Nachher | 0 | 37 |
|  | 1 | 50 |
| Geschlecht | 0 | 37 |
|  | 1 | 50 |

Geschlecht 0 = weiblich, 1=männlihc

Unpaired IKDC auch kein Unterschied.

| **Deskriptive Statistiken** | | | | |
| --- | --- | --- | --- | --- |
| Abhängige Variable: WOMAC-SCORE | | | | |
| Vorher_Nachher | Geschlecht | Mittelwert | Standardabweichung | N |
| 0 | 0 | 45,43 | 30,160 | 14 |
|  | 1 | 36,26 | 16,668 | 23 |
|  | Gesamt | 39,73 | 22,773 | 37 |
| 1 | 0 | 15,91 | 14,734 | 23 |
|  | 1 | 19,78 | 19,693 | 27 |
|  | Gesamt | 18,00 | 17,522 | 50 |
| Gesamt | 0 | 27,08 | 25,918 | 37 |
|  | 1 | 27,36 | 19,984 | 50 |
|  | Gesamt | 27,24 | 22,556 | 87 |

| **Tests der Zwischensubjekteffekte** | | | | | |
| --- | --- | --- | --- | --- | --- |
| Abhängige Variable: WOMAC-SCORE | | | | | |
| Quelle | Quadratsumme vom Typ III | df | Mittel der Quadrate | F | Sig. |
| Korrigiertes Modell | 10957,575 | 3 | 3652,525 | 9,244 | ,000 |
| Konstanter Term | 70504,430 | 1 | 70504,430 | 178,430 | ,000 |
| Vorher_Nachher | 10827,192 | 1 | 10827,192 | 27,401 | ,000 |
| Geschlecht | 143,901 | 1 | 143,901 | ,364 | ,548 |
| Vorher_Nachher * Geschlecht | 869,115 | 1 | 869,115 | 2,200 | ,142 |
| Fehler | 32796,356 | 83 | 395,137 |  |  |
| Gesamt | 108316,000 | 87 |  |  |  |
| Korrigierte Gesamtvariation | 43753,931 | 86 |  |  |  |

- Vorher-Nachher-Vergleich signifikant
- Geschlechtsunterschied nicht signifikant
- Wechselwirkung nicht signifikant, dh Vorher-Nachher gilt für beide Geschlechter gleichartig bzw. dass es keine Geschlechtsunterschiede gibt, gilt für beide Zeitpunkte gleichartig

**Univariate Varianzanalyse: Wie oben plus Kontrollvariablen**

| **Zwischensubjektfaktoren** | | |
| --- | --- | --- |
|  | | N |
| Vorher_Nachher | 0 | 36 |
|  | 1 | 50 |
| Geschlecht | 0 | 37 |
|  | 1 | 49 |

| **Deskriptive Statistiken** | | | | |
| --- | --- | --- | --- | --- |
| Abhängige Variable: WOMAC-SCORE | | | | |
| Vorher_Nachher | Geschlecht | Mittelwert | Standardabweichung | N |
| 0 | 0 | 45,43 | 30,160 | 14 |
|  | 1 | 34,32 | 14,147 | 22 |
|  | Gesamt | 38,64 | 22,094 | 36 |
| 1 | 0 | 15,91 | 14,734 | 23 |
|  | 1 | 19,78 | 19,693 | 27 |
|  | Gesamt | 18,00 | 17,522 | 50 |
| Gesamt | 0 | 27,08 | 25,918 | 37 |
|  | 1 | 26,31 | 18,735 | 49 |
|  | Gesamt | 26,64 | 21,974 | 86 |

| **Tests der Zwischensubjekteffekte** | | | | | |
| --- | --- | --- | --- | --- | --- |
| Abhängige Variable: WOMAC-SCORE | | | | | |
| Quelle | Quadratsumme vom Typ III | df | Mittel der Quadrate | F | Sig. |
| Korrigiertes Modell | 14706,106 | 6 | 2451,018 | 7,352 | ,000 |
| Konstanter Term | 14,017 | 1 | 14,017 | ,042 | ,838 |
| BMI | 68,329 | 1 | 68,329 | ,205 | ,652 |
| Alter | 3014,500 | 1 | 3014,500 | 9,042 | ,004 |
| Zeitpunkt | 925,609 | 1 | 925,609 | 2,776 | ,100 |
| Vorher_Nachher | 1604,409 | 1 | 1604,409 | 4,812 | ,031 |
| Geschlecht | 99,586 | 1 | 99,586 | ,299 | ,586 |
| Vorher_Nachher * Geschlecht | 1265,559 | 1 | 1265,559 | 3,796 | ,055 |
| Fehler | 26337,720 | 79 | 333,389 |  |  |
| Gesamt | 102075,000 | 86 |  |  |  |
| Korrigierte Gesamtvariation | 41043,826 | 85 |  |  |  |

- Alter relevant (passt zu Korrelationen ganz oben), BMI und Zeitpunkt nicht als Kontrollvariablen (Kovariate)
- Vorher-Nachher signifikant, Geschlecht nicht: Wie oben
- Wechselwirkung knapp nicht signifikant…siehe unten.

**Geschätzte Randmittel**

**1. Vorher_Nachher * Geschlecht**

| **Schätzer** | | | | | |
| --- | --- | --- | --- | --- | --- |
| Abhängige Variable: WOMAC-SCORE | | | | | |
| Vorher_Nachher | Geschlecht | Mittelwert | Standardfehler | 95%-Konfidenzintervall | |
|  |  |  |  | Untergrenze | Obergrenze |
| 0 | 0 | 40,350 | 5,855 | 28,696 | 52,004 |
|  | 1 | 29,812 | 4,878 | 20,101 | 39,522 |
| 1 | 0 | 18,514 | 4,322 | 9,912 | 27,116 |
|  | 1 | 23,867 | 4,446 | 15,019 | 32,716 |

| **Paarweise Vergleiche** | | | | | | | |
| --- | --- | --- | --- | --- | --- | --- | --- |
| Abhängige Variable: WOMAC-SCORE | | | | | | | |
| Geschlecht | (I)Vorher_Nachher | (J)Vorher_Nachher | Mittlere Differenz (I-J) | Standardfehler | Sig. | 95% Konfidenzintervall für die Differenz | |
|  |  |  |  |  |  | Untergrenze | Obergrenze |
| 0 | 0 | 1 | 21,836 | 7,624 | ,005 | 6,661 | 37,010 |
|  | 1 | 0 | -21,836 | 7,624 | ,005 | -37,010 | -6,661 |
| 1 | 0 | 1 | 5,945 | 7,438 | ,427 | -8,861 | 20,750 |
|  | 1 | 0 | -5,945 | 7,438 | ,427 | -20,750 | 8,861 |

| **Tests auf Univariate** | | | | | | |
| --- | --- | --- | --- | --- | --- | --- |
| Abhängige Variable: WOMAC-SCORE | | | | | | |
| Geschlecht | | Quadratsumme | df | Mittel der Quadrate | F | Sig. |
| 0 | Kontrast | 2735,061 | 1 | 2735,061 | 8,204 | ,005 |
|  | Fehler | 26337,720 | 79 | 333,389 |  |  |
| 1 | Kontrast | 212,937 | 1 | 212,937 | ,639 | ,427 |
|  | Fehler | 26337,720 | 79 | 333,389 |  |  |

- Im Detail nur bei Frauen ein signifikanter Unterschied Vorher-Nachher (MW von 45 auf 16 heruntergegangen, bei Männern nicht (aber auch hier von 34 auf 20 herunter, dh diese Wechselwirkung ist nicht interessant)…
- Also mit p=,055 als nicht signifikant betrachten.

**2. Vorher_Nachher * Geschlecht**

| **Schätzer** | | | | | |
| --- | --- | --- | --- | --- | --- |
| Abhängige Variable: WOMAC-SCORE | | | | | |
| Vorher_Nachher | Geschlecht | Mittelwert | Standardfehler | 95%-Konfidenzintervall | |
|  |  |  |  | Untergrenze | Obergrenze |
| 0 | 0 | 40,350 | 5,855 | 28,696 | 52,004 |
|  | 1 | 29,812 | 4,878 | 20,101 | 39,522 |
| 1 | 0 | 18,514 | 4,322 | 9,912 | 27,116 |
|  | 1 | 23,867 | 4,446 | 15,019 | 32,716 |

| **Paarweise Vergleiche** | | | | | | | |
| --- | --- | --- | --- | --- | --- | --- | --- |
| Abhängige Variable: WOMAC-SCORE | | | | | | | |
| Vorher_Nachher | (I)Geschlecht | (J)Geschlecht | Mittlere Differenz (I-J) | Standardfehler | Sig. | 95% Konfidenzintervall für die Differenz | |
|  |  |  |  |  |  | Untergrenze | Obergrenze |
| 0 | 0 | 1 | 10,538 | 6,663 | ,118 | -2,724 | 23,801 |
|  | 1 | 0 | -10,538 | 6,663 | ,118 | -23,801 | 2,724 |
| 1 | 0 | 1 | -5,353 | 5,820 | ,361 | -16,938 | 6,232 |
|  | 1 | 0 | 5,353 | 5,820 | ,361 | -6,232 | 16,938 |

| **Tests auf Univariate** | | | | | | |
| --- | --- | --- | --- | --- | --- | --- |
| Abhängige Variable: WOMAC-SCORE | | | | | | |
| Vorher_Nachher | | Quadratsumme | df | Mittel der Quadrate | F | Sig. |
| 0 | Kontrast | 834,024 | 1 | 834,024 | 2,502 | ,118 |
|  | Fehler | 26337,720 | 79 | 333,389 |  |  |
| 1 | Kontrast | 281,995 | 1 | 281,995 | ,846 | ,361 |
|  | Fehler | 26337,720 | 79 | 333,389 |  |  |

- Keine Geschlechtsunterschiede zu Vorher und zu Nachher gleichartig, dh das wie immer
- Die Kovarianzanalyse bringt keinen Mehrwert im Vergleich zur normalen Varianzanalyse
- Und die normale nur mit Geschlecht zusätzlich zu Vorher-Nachher bringt auch keinen Mehrwert im Vergleich zum t-Test, aber das wäre dennoch wohl xy
