# Supplementary material for: Intra-articular injection of bone marrow aspirate concentrate (mesenchymal stem cells) in KL grade III and IV knee osteoarthritis: 4 year results of 37 knees
Source: Sci Rep. 2024 Feb 1;14:2665. doi: 10.1038/s41598-024-51410-2 (PMC10834500; doi:10.1038/s41598-024-51410-2)
Supplement: Supplementary file 9 — Supplementary Information 9. [file 41598_2024_51410_MOESM9_ESM.docx]

**Univariat: Vergleich der fünf Zeitpunkte (0=vorher, 1-4 = Jahre nachher)**

| **ONEWAY deskriptive Statistiken** | | | | | | | | |
| --- | --- | --- | --- | --- | --- | --- | --- | --- |
| WOMAC-SCORE | | | | | | | | |
|  | N | Mittelwert | Standardabweichung | Standardfehler | 95%-Konfidenzintervall für den Mittelwert | | Minimum | Maximum |
|  |  |  |  |  | Untergrenze | Obergrenze |  |  |
| 0 | 37 | 39,73 | 22,773 | 3,744 | 32,14 | 47,32 | 6 | 96 |
| 1 | 20 | 25,05 | 20,067 | 4,487 | 15,66 | 34,44 | 0 | 67 |
| 2 | 10 | 14,20 | 9,830 | 3,108 | 7,17 | 21,23 | 0 | 25 |
| 3 | 8 | 12,38 | 18,943 | 6,697 | -3,46 | 28,21 | 2 | 58 |
| 4 | 12 | 13,17 | 14,584 | 4,210 | 3,90 | 22,43 | 0 | 53 |
| Gesamt | 87 | 27,24 | 22,556 | 2,418 | 22,43 | 32,05 | 0 | 96 |

| **Einfaktorielle ANOVA** | | | | | |
| --- | --- | --- | --- | --- | --- |
| WOMAC-SCORE | | | | | |
|  | Quadratsumme | df | Mittel der Quadrate | F | Signifikanz |
| Zwischen den Gruppen | 11712,542 | 4 | 2928,136 | 7,494 | ,000 |
| Innerhalb der Gruppen | 32041,389 | 82 | 390,749 |  |  |
| Gesamt | 43753,931 | 86 |  |  |  |

- Signifikanter Unterschied zwischen den fünf Zeitpunkten…wie und wo genau, siehe unten

**Post-Hoc-Tests**

| **Mehrfachvergleiche** | | | | | | |
| --- | --- | --- | --- | --- | --- | --- |
| Abhängige Variable: WOMAC-SCORE  Scheffé-Prozedur | | | | | | |
| (I) Zeitpunkt | (J) Zeitpunkt | Mittlere Differenz (I-J) | Standardfehler | Signifikanz | 95%-Konfidenzintervall | |
|  |  |  |  |  | Untergrenze | Obergrenze |
| 0 | 1 | 14,680 | 5,486 | ,139 | -2,61 | 31,97 |
|  | 2 | 25,530 | 7,045 | ,015 | 3,33 | 47,73 |
|  | 3 | 27,355 | 7,707 | ,018 | 3,06 | 51,64 |
|  | 4 | 26,563 | 6,567 | ,005 | 5,87 | 47,26 |
| 1 | 0 | -14,680 | 5,486 | ,139 | -31,97 | 2,61 |
|  | 2 | 10,850 | 7,656 | ,734 | -13,28 | 34,98 |
|  | 3 | 12,675 | 8,269 | ,673 | -13,39 | 38,74 |
|  | 4 | 11,883 | 7,218 | ,609 | -10,86 | 34,63 |
| 2 | 0 | -25,530 | 7,045 | ,015 | -47,73 | -3,33 |
|  | 1 | -10,850 | 7,656 | ,734 | -34,98 | 13,28 |
|  | 3 | 1,825 | 9,376 | 1,000 | -27,73 | 31,38 |
|  | 4 | 1,033 | 8,464 | 1,000 | -25,64 | 27,71 |
| 3 | 0 | -27,355 | 7,707 | ,018 | -51,64 | -3,06 |
|  | 1 | -12,675 | 8,269 | ,673 | -38,74 | 13,39 |
|  | 2 | -1,825 | 9,376 | 1,000 | -31,38 | 27,73 |
|  | 4 | -,792 | 9,023 | 1,000 | -29,23 | 27,64 |
| 4 | 0 | -26,563 | 6,567 | ,005 | -47,26 | -5,87 |
|  | 1 | -11,883 | 7,218 | ,609 | -34,63 | 10,86 |
|  | 2 | -1,033 | 8,464 | 1,000 | -27,71 | 25,64 |
|  | 3 | ,792 | 9,023 | 1,000 | -27,64 | 29,23 |

- Vorher und 1 Jahr unterscheiden sich nicht (p=,139). Es geht zwar Mittelwert von 39,7 auf 25,1 herunter, aber weil die Streuung zu diesen Zeitpunkten hoch ist, gibt es hier keinen signifikanten Unterschied
- Vorher vs. 2-4 Jahre unterscheiden sich signifikant (p=,015/,018/,005). Hier geht dann Mittelwert auf 12 bis 14 herunter…

WOW!
